# SET and HAT/PCET acid‐mediated oxidation processes in helical shaped fused bis‐phenothiazines

**DOI:** 10.1002/cphc.202100387

**Published:** 2021-06-17

**Authors:** Riccardo Amorati, Luca Valgimigli, Andrea Baschieri, Yafang Guo, Fabio Mollica, Stefano Menichetti, Michela Lupi, Caterina Viglianisi

**Affiliations:** ^1^ Department of Chemistry “G. Ciamician” University of Bologna Via S. Giacomo 11 40126 Bologna Italy; ^2^ Department of Chemistry “U. Schiff” University of Florence Via Della Lastruccia 3–13, Sesto Fiorentino 50019 Firenze Italy

**Keywords:** helicene radical, helicene radical cation, HAT, PCET, SET

## Abstract

Helical shaped fused *bis*‐phenothiazines **1**–**9** have been prepared and their red‐ox behaviour quantitatively studied. Helicene radical cations (Hel^.+^) can be obtained either by UV‐irradiation in the presence of PhCl or by chemical oxidation. The latter process is extremely sensitive to the presence of acids in the medium with molecular oxygen becoming a good single electron transfer (SET) oxidant. The reaction of hydroxy substituted helicenes **5**–**9** with peroxyl radicals (ROO^.^) occurs with a ‘classical’ HAT process giving HelO^.^ radicals with kinetics depending upon the substitution pattern of the aromatic rings. In the presence of acetic acid, a fast medium‐promoted proton‐coupled electron transfer (PCET) process takes place with formation of HelO^.^ radicals possibly also via a helicene radical cation intermediate. Remarkably, also helicenes **1**–**4**, lacking phenoxyl groups, in the presence of acetic acid react with peroxyl radicals through a medium‐promoted PCET mechanism with formation of the radical cations Hel^.+^. Along with the synthesis, EPR studies of radicals and radical cations, BDE of Hel‐OH group (BDE_OH_), and kinetic constants (*k_inh_*) of the reactions with ROO^.^ species of helicenes **1**–**9** have been measured and calculated to afford a complete rationalization of the redox behaviour of these appealing chiral compounds.

## Graphical abstract

**Oxidation of differently substituted thia[4]helicenes**: Single electron transfer and/or hydrogen atom transfer/proton‐coupled electron transfer processes operative on helical shaped fused bis‐phenothiazines are measured and studied rationalizing the role of substitution pattern and medium pH on the formation of Hel•^+^ radical cations and/or HelO• phenoxyl radicals.

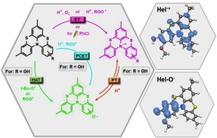

## 1 Introduction

**Figure 1:**
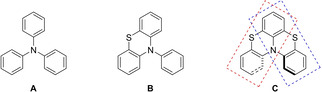
General structural skeletons of triaryl amines (**A**), *N*‐aryl phenothiazines (**B**) and thia‐bridged triarylamine hetero[4]helicenes (**C**).

Triaryl amines and structurally related *N*‐aryl phenothiazines (general skeletons **A** and **B** Figure 1) are well‐known for their ability to undergo a one electron oxidation to the corresponding radical cations *via* electrochemical or chemical oxidation. This peculiar redox behaviour makes them suitable to the development of easily oxidizable hole‐transporting materials.^[1]^ Indeed, these systems have found application as one‐electron donors in organic photo‐redox systems and electronic smart materials,^[2]^ devices such as dye‐sensitized solar cells (DSSCs),^[3]^ organic field‐effect transistors (OFET),^[4]^ organic light emission diodes (OLED)^[5]^ as well as two photon devices.^[6]^ In addition, phenothiazine skeleton is present in several drugs including antipsychotics or neuroleptics, such as chlorpromazine, thioridazine, and prochlorperazine.^[7]^

In this scenario, thia‐bridged triaryl amine hetero helicenes (*i. e*. the class of compounds with the general skeleton **C**, Figure 1) appear particularly appealing being *bis*‐phenothiazines with an aryl ring and a nitrogen atom in common, forced into a helical shaped structure by the long four carbon‐sulfur bonds.^[8]^ Indeed, compounds possessing skeleton **C** are among the rare examples of geometrically stable [4]helicenes with racemization energy barriers higher than those measured for all carbon [5]helicenes.[^8^, ^9^] Helical shaped derivatives possessing skeleton **C**, showed a very good one‐electron donor ability and can easily, and reversibly, be chemically oxidized to the corresponding stable, crystalline radical cations.^[10]^

This has paved the way to valuable applications, such as the preparation of redox active pH‐sensitive polymers.^[11]^ Additionally, organic radicals have been proposed as building blocks for several multifunctional devices,[^12^, ^13^] including spin filters for molecular spintronic devices,[^14^, ^15^] because of their relatively long spin coherence length. Thus, the possibility of tailoring the spin filtering exploiting the chiral induced spin selectivity (*CISS*) effect of the exceptionally stable radical cations obtained from compounds possessing skeleton **C**, is under development.^[16]^ This wide spectrum of applications required to study in detail the one‐electron oxidation behaviour of compounds possessing skeleton **C**, also by preparing hydroxy substituted derivatives capable to participate in proton‐coupled electron transfer (PCET) processes. In this contribution we report a detailed study of SET and HAT/PCET mediated oxidations of differently substituted thia[4]helicenes. Additionally, we rationalised the remarkable effect of medium pH on the oxidation processes, leading to radicals and/or radical cations of helical shaped bis‐phenothiazines, paving the way to the exploitation of their peculiar characteristics.

## 2 Results and Discussion

### 2.1 Synthesis

**Figure 2:**
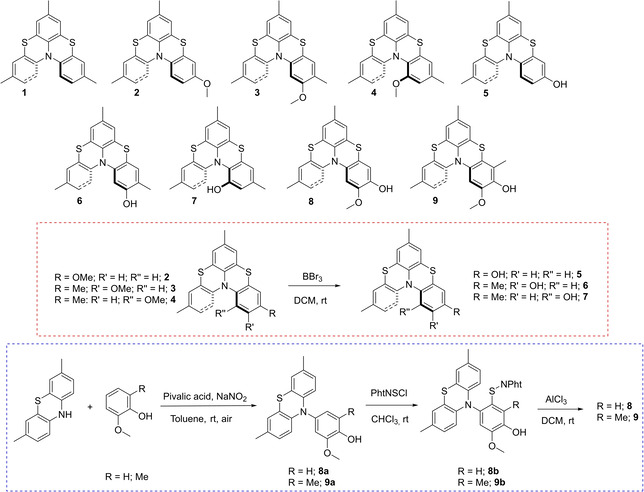
Structure of helical shaped *bis*‐phenothiazines **1**–**9** designed and prepared for this work.

Parent thia‐bridged triarylamine heterohelicene was prepared many years ago by means of two intramolecular Buchwald‐Hartwig processes.^[17]^ We have settled a new and more feasible procedure for the preparation of these systems based on the regioselective sulfenylation with phthalimidesulfenyl chloride PhtNSCl (Pht=Phthaloyl) of triarylamines or *N*‐aryl phenothiazines followed by a second Lewis acid promoted internal electrophilic sulfenylation.[^8^, ^10^] Recently, this procedure was further optimized for the preparation of asymmetric (not dissymmetric) derivatives.^[18]^ For this study we selected thia[4]heterohelicenes **1**–**9** (Figure 2, top) that were designed to have an identical phenothiazine sub‐unit (the red‐boxed left segment in Figure 1) and a differently substituted sub‐unit (the blue‐boxed right segment in Figure 1). Compounds **1**–**4** were prepared as previously described.[^8^, ^10^, ^18^] Hydroxy substituted derivatives **5**, **6** and **7**, are the result of BBr_3_ demethylation of the corresponding methoxy substituted helicenes **2**, **3** and **4** (Figure 2 middle and experimental section). New hydroxy substituted bis‐phenothiazines **8** and **9** were prepared, as previously mentioned, from the corresponding properly designed N‐aryl phenothiazines (Figure 2 bottom, experimental and Supplementary Information sections).

### 2.2 Formation and properties of the radical species derived from 1–9

**Figure 3:**
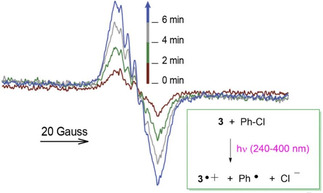
Increase of the EPR signal of the radical cation **3** ^.+^ upon UV irradiating (240‐400 nm) in the EPR cavity a nitrogen fluxed solution of **3** in benzene (4.5 mM) containing 10 % (v/v) PhCl.

The spin distribution in the radical cations of the title helicenes (HelO^.+^) were studied by EPR spectroscopy. It was reported that phenothiazines are transformed into the corresponding radical cations under hard acid conditions.^[19]^ In our systems, the radical cations could be generated simply by mixing a dilute benzene solution (10^−4^ M) of the helicene with CF_3_COOH (1.2 M) in the presence of air. A deep colour rapidly developed and EPR spectra showed the typical signals of phenothiazine‐like radical cations (see Table 1). In the case of the helicene **1**, the EPR spectrum obtained by acidification was identical to that generated by reaction with tris(4‐bromophenyl)ammoniumyl hexachloroantimonate (TBPN^.+^, a commercially available stable radical cation known as ‘magic blue’), and with that of authentic radical cation **1** ^.+^ synthetized by reaction of **1** with Ag(SbF_6_),^[10a]^ indicating that all three methods provide the same radical species. In the case of the *bis*‐phenothiazines **5**–**9** bearing an OH group, radical cations could be generated only by acidification with CF_3_COOH, while the reaction with TBPN^.+^ was unsuccessful, reasonably because, under non‐acid conditions, the helicene radical cations quickly deprotonate to form short‐living neutral phenoxyl radicals. Interestingly, radical cations were formed also in the absence of oxygen, by irradiating at 240–400 nm helicene solutions in the presence of chlorobenzene (see for instance Figure 3). Formation of radical cations when irradiating *N*‐methylphenothiazine (MPT) in the presence of halogenated compounds (R−X) was previously attributed to electron donation from the excited triplet state of MPT to RX, to form X^−^ and alkyl radicals (R^.^) that were identified thanks to their typical reactivity.^[20]^ We have imagined a similar behaviour operative in our systems with formation of transient phenyl radical from chlorobenzene (Figure 3).

**Table 1:** EPR parameters (hyperfine spitting constants, *hfsc*, and *g*‐factors) and BDE_OH_ for helicenes **1**–**9**.

Compound	Radical	*hfsc* (gauss)^[a]^	*g* ^[a]^	BDEO−H kcal/mol^[b]^
**1**	**1^.+^**	1 N:7.86; 2H: 1.06; 9H:2.26; 4H: 0.49; 2H:0.31^[c]^	2.0042	
**2**	**2^.+^**	1 N: 7.83; 3H: 1.88; 3H: 0.99; 3H: 2.22; 1H: 1.48; 1H: 1.61	2.0060	
**3**	**3^.+^**	1 N: 6.94, 3H: 2.04; 3H: 2.23; 3H: 1.81; 1H: 1.01; 1H: 0.51; 3H: 1.34	2.0063	
**4**	**4^.+^**	1 N: 7.07; 3H: 3.43; 3H: 2.80; 3H: 2.20	2.0050	
**5**	**5^.+^**	1 N: 7.76; 3H: 1.62; 3H: 0.94; 1H: 2.33; 1H: 2.17; 1H: 2.02	2.0044	
	**5(‐H)^.^**	1 N: 3.18; 1H: 0.93; 1H: 0.83; 1H: 0.52	2.0042	79.0±0.2 (80.0)
**6**	**6^.+^**	1 N: 6.80; 3H: 3.20; 3H: 1.26; 3H: 2.27; 1H: 2.74; 1H: 2.84; 1H: 1.60^[d]^	2.0060	
	**6(‐H)^.^**	N: 1.52, 3H: 3.70, 1H: 2.96; 1H: 2.44; 6H: 0.66	2.0042	78.6±0.5 (81.4)
**7**	**7^.+^**	1 N: 7.44; 3H: 2.17; 3H: 2.30; 3H: 2.17, 1H: 0.36	2.0045	
	**7(‐H)^.^**	1 N: 1.25; 1H: 1.22; 1H: 3.39	2.0044	80.3±0.5 (82.2)
**8** ^[e]^	**8^.+^**	N: 5.81, 3H: 0.52; 3H: 1.23; 1H: 0.90; 1H: 1.03; 1H: 1.99	2.0057	
	**8(‐H)^.^**	N: 2.17; 1H: 2.81; 3H: 1.36; 1H: 0.78	2.0048	79.6±0.2 (81.1)
**9**	**9^.+^**	1 N: 6.84; 1H: 2.25; 3H: 1.86; 3H: 1.46; 1H: 0.98; 1H: 0.81	2.0051	
	**9(‐H)^.^**	1 N: 1.96; 3H: 4.61; 3H: 1.33; 1H: 0.76	2.0044	77.8±0.4 (80.1)

[a] In benzene, 25 °C. [b] Experimental BDE_OH_ in benzene, in round brackets calculated BDE. [c] In MeCN, 1 N: 7.77; 2H: 1.04; 9H: 2.25. [d] Recorded in the presence of p‐TSOH; the use of CF_3_COOH instead provided the following *hfsc*: 3H: 4.29, 1H: 2.10, 1 N: 0.81. [e] The reaction **8** with TBPN^.+^ in MeCN did not afford any EPR new signal.

Neutral phenoxyl radicals (HelO^.^) were instead generated photolytically, upon irradiating **5**–**9** in the presence di‐*tert*‐butyl peroxide (TBP) as the source of alkoxyl radicals ^t^BuO^.^, in deoxygenated benzene solutions (see Equation (1) for helicene **5**). This procedure did not afford any detectable radical for helicenes **1**–**4** lacking the OH group.

(1) $${}^{t}\mathrm{BuOO}{}^{t}\mathrm{Bu}\to 2\;{}^{t}\mathrm{BuO}{}^{•}+5\to {}^{t}\mathrm{BuOH}+5\left(\text{-}H\right){}^{•}$$

**Figure 4:**
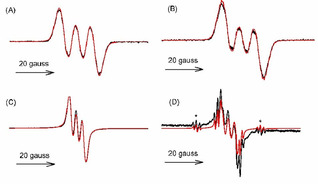
Experimental (black) and simulated (red) EPR spectra in benzene of: A) **5** ^.+^ ; B) **2** ^.+^ ; C) **5**(**‐**H)^.^ ; D) **5**(**‐**H)^.^ and BHT(‐H)^.^, asterisks indicate the outer triplets of BHT(‐H)^.^.

The *g* factors and the hyperfine splitting constants, *hfsc* (a), obtained by numerical fitting of the EPR spectra (see Figure 4 and Table 1) allowed the identification of all the radical species. Radical cations are characterized by a significant coupling of the unpaired electron with the N‐atom (a_N_=6‐8 gauss) in line with previous reports and with DFT calculations, and by small coupling with all methyls (a_H_ ≈2 gauss) or hydrogens (a_H_≤2 Gauss) linked to the aromatic systems, indicating delocalization of the unpaired electron on all the three benzene rings (see Table 1 and Figure 4 traces A and B). In the case of **6**, a_N_ was unusually small (0.81 Gauss), conceivably because the radical cation underwent partial deprotonation at the equilibrium, forming two rapidly exchanging species. In fact, the typical a_N_ of a radical cation was observed by adding an acid stronger than CF_3_COOH like *p*‐toluenesulfonic acid (p‐TsOH, see Table 1).

Neutral phenoxyl radicals are much less persistent than radical cations, and their spectrum can be recorded only by continuous in‐cavity irradiation of the solution in the presence of di‐*tert*‐butyl peroxide. They are characterized by a smaller nitrogen hyperfine splitting constant (1 < a_N_ <3) in qualitative agreement with calculations and by bigger constants with the methyl groups *ortho* to the OH (a_H_ ≈4 Gauss), compared to the corresponding radical cations (Table1 and Figure 4 trace C). These values, however, are smaller than those calculated by DFT methods (see supporting information) or expected from literature data.^[21]^ We tentatively explain this result as an effect of the formation of a mixture of neutral and cation radicals in fast equilibrium. The *g‐*factors of the radical cations of helicenes are slightly larger than those of the phenoxyl radicals (see Table 1), indicating delocalization of the unpaired electron on S atoms.^[22]^

### 2.3 O−H Bond Dissociation Enthalpy (BDE_OH_)

The bond dissociation enthalpy of the phenolic OH bond in helicenes **5**–**9** was determined experimentally by using the EPR‐equilibration technique,[^21^, ^22^] and by theoretical DFT calculations. The EPR method consists of measuring the equilibrium constant, *K*_eq_, for the hydrogen‐atom transfer between a reference phenol (ArOH), in this case 2,6‐di‐*tert*‐butyl‐4‐methylphenol (BHT, BDE_OH_=79.9 kcal mol^−1^)^[23]^ and the helicene phenoxyl radicals (HelO^.^), as shown in equation (2). The phenoxyl radicals are generated under continuous photolysis in deoxygenated benzene containing di‐*tert*‐butyl peroxide, at controlled temperature, as shown in Figure 4D. In equation (2), the initial concentrations of HelOH and ArOH were used, and the relative radical concentrations were determined by means of numerical fitting of the EPR spectra showing the superimposition of the two radicals (see Figure 4D).

(2) $$\mathrm{HelOH}+\mathrm{ArO}{}^{•}\vec{\leftarrow }\mathrm{HelO}{}^{•}+\mathrm{ArOH}$$

(3) $$\mathrm{BDE}\left(\mathrm{HelO}-H\right)=\mathrm{BDE}\left(\mathrm{ArO}-H\right)-\mathrm{RTln}\left(K{}_{\mathrm{eq}}\right)$$

The BDE for HelOH **5**–**9** was obtained, under the assumption that the entropic term can be neglected,^[24]^ by means of equation (3) from *K*_eq_ and the known BDE_OH_ value of ArOH. The experiments were repeated at least three times at different HelOH/ArOH ratios.

**Figure 5:**
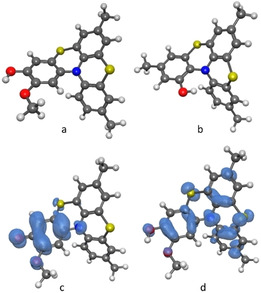
Optimized structures and spin densities of **8** (a), **7** (b), phenoxyl radical **8**(‐H)^.^ (c) and radical cation **8** ^.+^ (d).

The BDE_OH_ was also calculated by means of DFT theoretical methods by using the isodesmic approach that consists of calculating the BDE_OH_ difference between helicenes (HelOH) and phenol (PhOH), and adding it to the experimental BDE_OH_ of phenol, which is known with high accuracy.^[25]^ The structure of the helicenes and those of the corresponding phenoxyl radicals HelO^.^ were optimized at the B3LYP/6‐311+g(d,p) level (Figure 5). The BDE_OH_ of helicenes was obtained by equations (4) and (5) by using the reference value for BDE_OH_ of phenol in benzene as 86.7 kcal/mol.^[25]^

(4) $$\Delta \mathrm{BDE}=\left[\Delta H\left(\mathrm{HelO}{}^{•}\right)-\Delta H\left(\mathrm{HelOH}\right)\right]-\left[\Delta H\left(\mathrm{PhO}{}^{•}\right)-\Delta H\left(\mathrm{PhOH}\right)\right]$$

(5) BDE(HelO-H)=BDE(PhO-H)+ΔBDE

The calculated BDE_OH_ values were in good agreement with measured ones (see Table 1). Results can be rationalized on the basis of the additive rules to account for the effect of ring substituents on phenolic BDE_OH_, developed by Pedulli and co‐workers.^[23]^ These rules allow the comparison of the results obtained for the different helicenes, and with the previously reported BDE_OH_ of other phenols. The BDE_OH_ of **5** is 79.0 kcal/mol, that is 7.7 kcal/mol smaller than that of parent phenol (86.7 kcal/mol).^[23]^ Considering that substituents in *meta* position with respect to the OH group are only marginally influent (electron‐donating substituents typically lower the BDE by ∼0.5 kcal/mol),^[23]^ the low BDE of **5** can be mostly ascribed to the stabilizing effect of the *para*‐nitrogen atom on the phenoxyl radical. This stabilization, however, is smaller than that observed with aliphatic amines (‐10 kcal/mol),^[23]^ due to the delocalization of the nitrogen lone pair on the other two aromatic rings.

Helicene **8** has a BDE_OH_ (79.6 kcal/mol) nearly identical to that of helicene **5**, because the radical stabilizing effect of the OMe group is counterbalanced by the formation of an OH–OMe intramolecular H‐bond that stabilizes the phenol (see Figure 5a).^[26]^

The methyl group *ortho* to the OH further lowers the BDE_OH_ of helicene **9** by 1.2 kcal/mol (overall 77.8 kcal/mol), in line with the expected value based on the additive effect of Me groups (−1.7 kcal/mol).^[23]^ The low BDE_OH_ value of **6** (78.6 kcal/mol) is due to the radical stabilizing effect of the methyl group (−1.2 kcal/mol) and of the *para*‐sulfur and *meta‐*nitrogen atoms (overall contribution −6.9 kcal/mol). The relatively large BDE_OH_ measured in compound **7** (80.3 kcal/mol) can instead be attributed to the occurrence of intramolecular interaction between the OH group and the *ortho*‐nitrogen atom,^[26]^ whose formation is confirmed also by DFT calculations (see Figure 5b).

### 2.4 Kinetics of Reaction with Peroxyl Radicals

**Figure 6:**
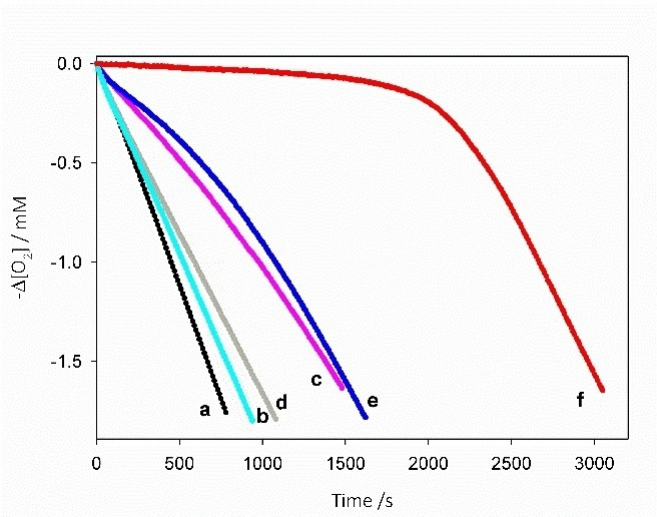
Oxygen consumption during the autoxidation of styrene (4.3 M) initiated by AIBN (0.05 M) in acetonitrile at 30 °C without inhibitors (**a**) or in the presence of **1** (**b**), **5** (**c**), acetic acid 0.5 % v/v (**d**), **1** and acetic acid 0.5 % v/v (**e**), **5** and acetic acid 0.5 % v/v (**f**). [**1**]**=**[**5**]**=**4.5×10^−6^ M.

The rate constants for the reaction of helicenes **1**–**9** with alkylperoxyl radicals, *k*_inh_ (see Figure 6 and Table 2) were measured in chlorobenzene and acetonitrile by studying the autoxidation of styrene inhibited by varying amounts of the helicenes.[^27^, ^28^, ^29^] In the absence of inhibitors, the O_2_ consumption observed during the autoxidation of styrene initiated by AIBN at 30 °C is fast (see black line in Figure 3), while, in the presence of molecules able to trap ROO^.^ radicals, O_2_ uptake is slowed down. The rate constant of the reaction between inhibitors and ROO^.^ can be obtained from the slopes of O_2_ *vs* time plots (see experimental section). In chlorobenzene, helicenes **5**–**9**, having a hydroxyl group, showed a high *k*_inh_ value (>10^5^ M^−1^ s^−1^), while, not surprisingly, those lacking the hydroxyl group did not retard styrene autoxidation, indicating *k*_inh_<10^3^ M^−1^ s^−1^.

**Table 2:** Rate constant of reaction of helicenes **1**–**9** with ROO^.^ and stoichiometry of radical trapping (*n*).

Compound	PhCl		ACN		ACN+CH_3_COOH 0.5 %	
	*k*_inh_×10^5^ M^−1^ s^−1^	n	*k*_inh_×10^5^ M^−1^ s^−1^	*n*	*k*_inh_×10^5^ M^−1^ s^−1^	*n*
**1**	–	–	–	–	1.0±0.2	0.9±0.1
**2**	–	–	–	–	1.9±0.2	0.9±0.1
**3**	–	–	–	–	1.4±0.2	1.2±0.1
**4**	–	–	–	–	2.3±0.3	2.0±0.2
**5**	9.5±1.1	2.2±0.1	1.5±0.1	1.3±0.1	29±1.6	2.3±0.3
**6**	3.4±0.2	1.3±0.1	1.9±0.2	0.9±0.1	8.5±0.9	0.9±0.1
**7**	1.7±0.3	1.8±0.2	1.8±0.2	0.9±0.1	2.1±0.1	1.7±0.4
**8**	6.0±0.9	1.2±0.1	1.8±0.3	1.6±0.0	21±0.8	2.0±0.1
**9**	6.4±0.2	1.2±0.2	2.0±0.3	1.0±0.2	54±0.8	0.9±0.1

In acetonitrile, H‐bond formation between the solvent and the reactive OH caused a decrease of *k*_inh_ values, as observed for helicene **5**. On the other hand, the magnitude of this kinetic solvent effect, which is well‐known for phenolic compounds,^[26]^ depends upon the substituents in *ortho* to the OH group. Methyl and *ortho*‐methoxyl groups protect the hydroxyl group from H‐bond formation with solvent. Accordingly, the solvent effect is less pronounced for helicenes **6**, **8** and **9**, while it is completely abolished for hindered compound **7**, having indistinguishable *k*_inh_ in chlorobenzene or in acetonitrile. Stoichiometry of peroxyl radical trapping (*n*) was close to unit for most OH bearing helicenes (Table 2) at variance with the typical value *n*=2 of simpler phenols. This is possibly due to the limited attitude of Hel−O^.^ phenoxyl radicals to add peroxyl radicals due to steric hindrance in the positions of highest spin density, and to preserve the planar conjugated structure.

Since EPR studies have shown that acids affect the ease of radical formation from helicenes, we next set to investigate the role of added acids on their reactivity with peroxyl radicals. Addition of acetic acid (0.5 % vol/vol) to helicenes in acetonitrile caused a marked increase of the inhibition of styrene autoxidation. For instance, helicene **5** was only a moderate inhibitor (line **c** in Figure 6), but after the addition of acetic acid a very strong inhibition of the autoxidation was observed (line **f** in Figure 6). Interestingly, this effect was visible also for helicenes **1**–**4** lacking the OH substituent. Indeed, by lowering medium pH the rate of ROO^.^ radicals trapping of compounds **1**–**4** greatly increases with a consequent slowing down of O_2_ uptake, see for instance trace **b** vs trace **e** in Figure 6.

We interpret this result by considering that helicenes are good electron donors, therefore, in the presence of a proton donor they can react with peroxyl radicals via a proton‐coupled electron transfer (PCET) mechanism assisted by the reaction medium.

**Figure 7:**
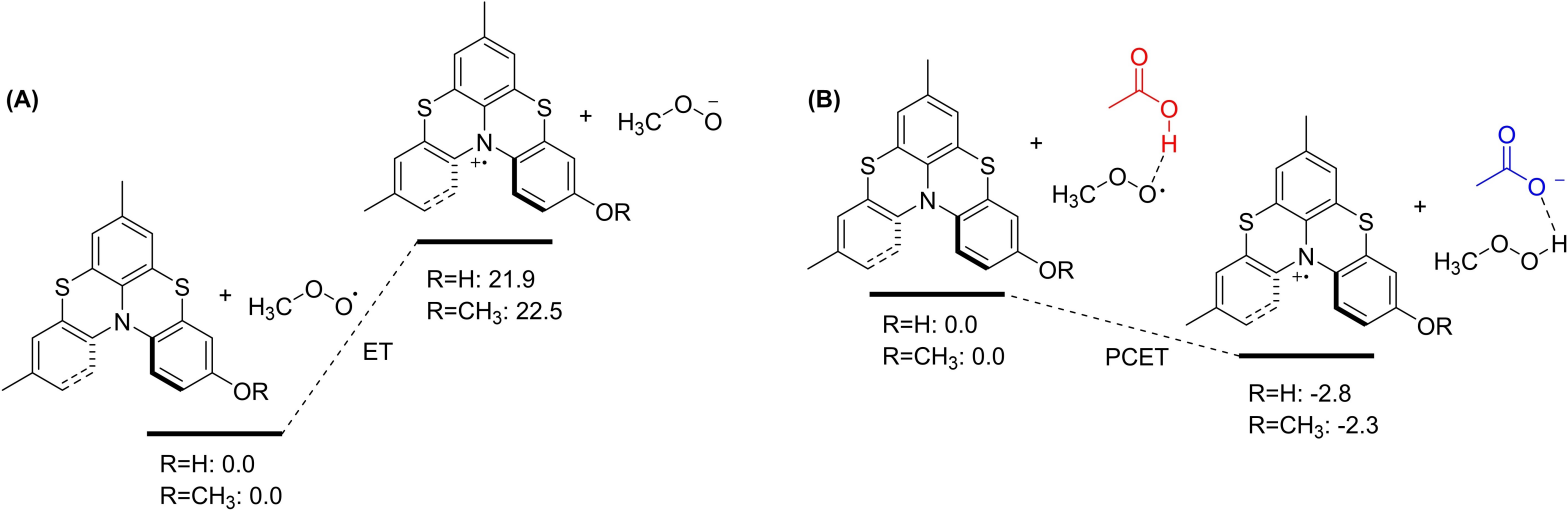
DFT‐computed free energy for the electron transfer from helicenes **2** (R=CH_3_) and **5** (R=H) toward CH_3_OO^.^ radical in the absence **(A)** or in the presence **(B)** of acetic acid. Proton transfer from CH_3_COOH to CH_3_OO^−^ is barrierless and occurs during the geometry optimization. Calculations performed at the B3LYP/6‐311+g(d,p) level with implicit MeCN solvent and one explicit MeCN molecule when R=H.

To investigate in deeper detail the effect of acetic acid on the reaction of helicenes with peroxyl radicals, we calculated the free energy change of the electron transfer (ΔG_ET_) step of equations (6) and (7) in MeCN as the solvent, for helicenes **5** and **2**, taken respectively as models of compounds bearing or not the OH function, using CH_3_OO^.^ as the alkylperoxyl radical model. In the absence of added acid, the reaction is highly endergonic for both helicenes, with calculated ΔG_ET_ around +22 kcal/mol, see Figure 7A, while, in the presence of acetic acid this would be H‐bonded to the strong acceptor CH_3_OO^.^, transforming it in a much stronger oxidizing species. Indeed, the ET reaction becomes mildly exergonic with calculated ΔG_ET_ of −2.8 and −2.3 kcal/mol respectively for helicenes **5** and **2**, Figure 7B. This huge stabilization along the reaction path is due to a barrierless (*i. e*. occurring during the geometry optimization) proton transfer from CH_3_COOH to CH_3_OO^−^, confirming that in the presence of acid the reaction can better be described as a PCET process.

(6) $$\mathrm{Hel}+\mathrm{ROO}{}^{•}+\mathrm{AcOH}\to \mathrm{Hel}{}^{•}{}^{+}+\mathrm{AcO}{}^{-}+\mathrm{ROOH}$$

(7) $$\mathrm{HelOH}+\mathrm{ROO}{}^{•}+\mathrm{AcOH}\to \mathrm{HelOH}{}^{•}{}^{+}+\mathrm{AcO}{}^{-}+\mathrm{ROOH}\to \mathrm{HelO}{}^{•}+\mathrm{AcOH}+\mathrm{ROOH}$$

**Figure 8:**
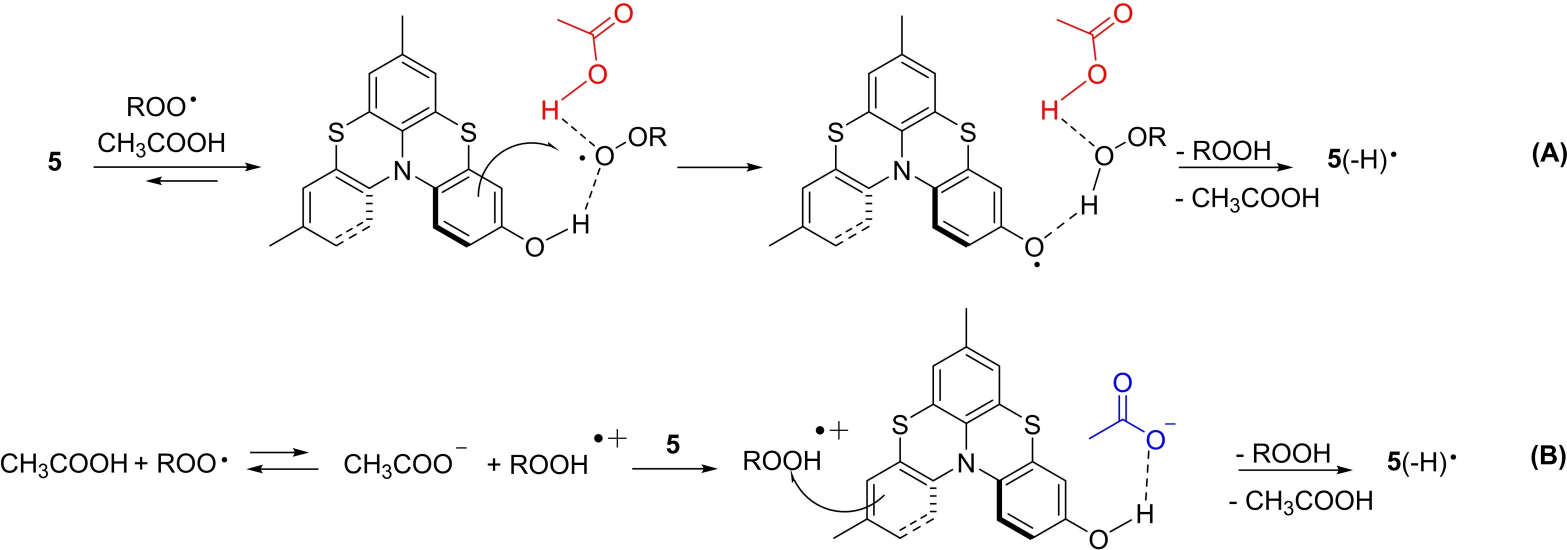
PCET mechanisms, PCET **(A)** or ET plus PT **(B)**, explaining the enhanced reactivity of phenolic helicenes **5**–**9** with alkylperoxyl radicals in the presence of acetic acid.

While the PCET mechanism illustrated in Figure 7B nicely accounts for the efficient reaction of helicene **2** with ROO^.^, despite the absence of transferable hydrogens, the 15‐fold faster reaction of **5** compared to **2** in the presence of 0.5 % of acetic acid, cannot be justified solely on the basis of the marginally higher calculated exergonicity (ΔΔG_ET_=‐0.5 kcal/mol, see Figure 7B). We suggest that all helicenes bearing the phenolic function undergo the acid assisted mechanism described above with additional assistance from H‐bonding to ROO^.^ (in turn H‐bonded to CH_3_COOH), which will allow the proton transfer from the phenolic OH concerted with the ET to the H‐bonded peroxyl radical, Figure 8A. For simpler phenols, this PCET was previously demonstrated by some of us to have lower barrier than uncatalyzed reactions.^[27]^ An alternative (or competitive) mechanism would be a separated PCET process, consisting of ET to the protonated peroxyl radicals and PT to the medium (acetate), as depicted in Figure 8B. Despite the unfavourable acid‐base equilibrium to afford the protonated peroxyl radical, this reaction was calculated to be barrierless, hence dominating in the case of simpler phenols in the presence of carboxylic acids.^[27]^

## 3 Conclusions

Helical shaped *bis*‐phenothiazines, like derivatives **1**–**9** prepared for this study, have found interesting opportunities in material science[^11^, ^16^] and a detailed quantitative investigation of their red‐ox one‐electron properties appeared a mandatory step to underpin their peculiar characteristics.

We have demonstrated and measured SET and/or HAT/PCET processes operative on these systems depending upon the substitution pattern and the medium. Indeed, under acidic condition, molecular oxygen can be used as an efficient oxidant to generate the radical cations of **1**–**9** (Hel^.+^). Radical cations can be obtained also by UV irradiation (240‐400 nm) in the presence of chlorobenzene. By studying the reaction of **1**–**9** with peroxyl radical we demonstrated that under acidic conditions a proton‐coupled electron transfer mechanism becomes operative, leading, initially, to a radical cation. Radical cations and phenoxyl radicals of helicenes **5**–**9** are indeed in rapid equilibrium by deprotonation / protonation. On the other hand, phenoxyl radicals of **5**–**9** can be obtained by reaction with alkoxy or peroxyl radicals under neutral conditions and their stability (in terms of *k_inh_* and Hel‐OH BDE) can be predicted using the additive rules typically used for phenols. Quantitative knowledge of medium effects on the redox behaviour of helicenes allows its rational manipulation and better design of applications, *e. g*. for their use as chiral spin filters, which is currently under development in our group.^[16]^ We believe that the distinctive properties of these molecules will attract many researchers, in the light of the synthetic accessibility in continuing improvement in our laboratories.

## Experimental Section

### Materials

^1^H and ^13^C NMR spectra were recorded with Varian Mercury Plus 400, Varian Inova 400 and Varian Gemini 200, using CDCl_3_, CD_2_Cl_2_ and (CD_3_)_2_CO), as solvents. Residual CHCl_3_ at *δ*=7.26 ppm, Residual CHDCl_2_ at *δ*=5.32 ppm and residual (CHD_2_)_2_CO at *δ*=2.05 ppm were used as the reference of ^1^H‐NMR spectra. Central lines of: CDCl_3_ at *δ*=77.00 ppm, (CD_3_)_2_CO at *δ*=29.84, were used as the reference of ^13^C‐NMR spectra. FT‐IR spectra were recorded with Spectrum Two FT‐IR Spectrometer. ESI‐MS spectra were recorded with a *J*EOL MStation *J*MS700. Melting points were measured with Stuart SMP50 Automatic Melting Point Apparatus. All the reactions were monitored by TLC on commercially avail‐ able precoated plates (silica gel 60 F 254) and the products were visualized with acidic vanillin solution. Silica gel 60 (230–400 mesh) was used for column chromatography. Dry solvents were obtained by The PureSolv Micro Solvent Purification System. Chloroform was washed with water several times and stored over calcium chloride. Pyridine and TEA were freshly distilled from KOH. CF_3_COOH, acetic acid, tert‐butylperoxide (^t^BuOO^t^Bu), were of the highest purity available and used as received. Acetonitrile, benzene, and chlorobenzene were of HPLC‐grade. Styrene was percolated twice on alumina, AIBN was recrystallized from MeOH.

Phthalimide sulfenyl chloride was prepared from the corresponding disulfide as reported elsewhere.^[8]^ Helicenes **1**, **2**, **3** and **4** were described elsewhere.[^8^, ^18^] Preparation of the starting materials for the synthesis of helicenes **8** and **9** is available as Supplementary Information.

### EPR Experiments

The X‐band EPR spectra were collected in quartz tubes with Elexsys 500 (Bruker) and a MiniScope MS 5000 (Magnettech), both equipped with temperature control. UV irradiation in cavity was provided by an optical fiber from a mercury‐xenon lamp (Hamamatsu Lightingcure LC8, 240–400 nm). Solutions were deoxygenated by prolonged N_2_ bubbling in the tube. Radical cations and neutral radicals were generated by adding 10 % CF_3_COOH or 10 % ^t^BuOO^t^Bu, respectively, to a 3–10 mM sample solution in benzene. EPR equilibration experiments were performed by mixing the concentrated solutions of the investigated compounds and of the reference phenol (2,6‐di‐*tert*‐butyl‐4‐methylphenol) with the addition of 10 % ^t^BuOO^t^Bu inside a quartz tube, followed by N_2_ bubbling.[^21^, ^22a^, ^28^] Spectra were analysed by the WinESR program. Measured *g*‐factors were corrected with respect of 2,2,6,6‐tetramethylpiperidine‐N‐oxyl (TEMPO) radical, *g*=2.0062,^[30]^ and that of 2,2‐diphenyl‐1‐picrylhydrazyl (DPPH) radical in benzene, *g*=2.00364.^[26b]^

### Autoxidation Experiments

Autoxidation were performed in a two‐channel oxygen uptake apparatus, based on a Validyne DP 15 differential pressure transducer built in our laboratory.[^26^, ^27^, ^28^, ^29^, ^30^] The peroxyl radical‐trapping activity was evaluated by studying the inhibition of the thermally initiated autoxidation of styrene in chlorobenzene or acetonitrile. In a typical experiment, an air‐saturated mixture of the oxidizable substrate and the solvent, 1 : 1 (v/v), containing AIBN (0.05 M) as an initiator was equilibrated with an identical reference solution containing an excess of 2,2,5,7,8‐pentamethyl‐6‐chromanol (PMHC). After equilibration, and when a constant O_2_ consumption was reached, a concentrated solution of the antioxidant (final concentration=2÷10 μM) was injected in the sample flask. The oxygen consumption in the sample was measured after calibration of the apparatus from the differential pressure recorded with time between the two channels. Initiation rates, R_i_, were determined by the inhibitor method, by using PMHC as a reference antioxidant: R_i_=2[PMHC]/τ, in which τ is the length of the induction period. Inhibition constants *k*_inh_ were determined by equation (8) which relates the rates of the inhibited and non‐inhibited autoxidation (R_in_ and R_0_, respectively) to the rate constant *k*_inh_, the initiation rate R_i_, the concentration of the antioxidant [AH] and the stoichiometry of radical trapping (*n*). Typical R_i_ was 3.1×10^−9^ Ms^−1^, while the rate constant for chain termination of styrene at 30 °C is 2*k*_t_=4.2×10^7^ M^−1^ s^−1^.^[30]^

(8) $$\left(R{}_{0}/R{}_{\mathrm{in}}\right)-\left(R{}_{\mathrm{in}}/R{}_{0}\right)=n\;k{}_{\mathrm{inh}}\;\left[\mathrm{AH}\right]/\left(2k{}_{t}R{}_{i}\right){}^{1/2}$$

### DFT Calculations

Geometry optimization and frequencies were computed at the B3LYP/6‐311+g(d,p) level by using Gaussian 09. Stationary points were confirmed by checking the absence of imaginary frequencies. For the calculation of electron transfer enthalpies, the solvent was modelled by the standard self‐consistent reaction field procedure as implemented in the Gaussian 09 set of programs, and in the case of **5** an explicit MeCN molecule hydrogen bonded to the OH group was used.

### Synthesis

General Procedure for the Synthesis of hydroxy‐substituted helicenes **5**, **6** and **7** by demethylation with BBr_3_ of the corresponding methoxy helicenes **2**, **3** and **4**. To a solution of the helicene in dry DCM (roughly 0.1 M), under a nitrogen atmosphere, BBr_3_ (1÷3 eq.) were added at 0 °C and the sparkly coloured solution stirred at room temperature till the complete disappearance of methoxy derivative monitored by TLC (3÷24 h). The reaction mixture was diluted with DCM, washed twice with a saturated solution of NaHCO_3_ and with H_2_O. The organic layer was dried over Na_2_SO_4_, filtered, and evaporated under reduced pressure. The crude product was purified by flash chromatography on silica gel.

**3‐hydroxy‐7,11‐dimethyl[1,4]benzothiazino[2,3,4‐kl]phenothiazine (5)**: Following the general procedure from **2** (70 mg, 0.19 mmol) and 1 eq. of BBr_3_ kept 10 min at 0 °C and 24 h at rt. The crude was purified by flash chromatography on silica gel (Petroleum Ether/DCM: 1/3) to obtain helicene **5** (30 mg, 45 % yield) as a grey solid. M.p. 235 °C (dec.). ^1^H NMR* (400 MHz, CDCl_3_) δ: 2.20 (s, 3H), 2.26 (s, 3H), 6.56 (bd, *J*=4 Hz, 1H), 6.64 (bs, 1H), 6.76−6.77 (m, 2H), 6.89 (bd, *J*=8.3 Hz, 1H), 6.98−7.02 (m, 3H) ppm. ^13^C NMR (100 MHz, CDCl_3_)* δ: 20.5, 20.7, 114.6, 115.2, 119.8, 121.6, 125.0, 125.6, 126.0, 126.1, 126.2, 128.1(2 C), 133.8, 134.3, 134.9, 137.7, 140.9, 153.9 ppm (19 signals for 20 different carbons). IR (ATR solid) 1/λ: 1194, 1313, 1448, 1486, 1582, 3343 cm^−1^. ESI‐MS negative mode, *m/z*=348 [M−1]^−^, 697 [2M−1]^−^. Elem. Anal. for C_20_H_15_NOS_2_: Calcd. C 68.74, H 4.33, N 4.01; found C 68.72, H 4.31, N 4.00. *Et_3_N was added to neutralize CHCl_3_ acidity.

**2‐hydroxy‐3,7,11‐trimethyl[1,4]benzothiazino[2,3,4‐kl]phenothiazine (6)**: Following the general procedure from **3** (40 mg, 0.11 mmol) and 3 eq. of BBr_3_ kept for 10 min at 0 °C and 3 h at rt. The crude was purified by flash chromatography on silica gel (Petroleum Ether/DCM: 1/2) to obtain helicene **6** (31 mg, 78 % yield) as a white solid. M. p. 128 °C. ^1^H NMR (200 MHz, CDCl_3_) δ: 2.17 (bs, 3H), 2.20 (s, 3H), 2.28 (s, 3H), 4.65 (s, 1H), 6.61 (bs, 1H), 6.77 (bs, 2H), 6.88‐6.92 (m, 2H), 6.99 (bs, 1H), 7.08 (bd, 1H, *J*=8.2 Hz) ppm. ^13^C NMR (100 MHz, CDCl_3_) δ: 15.3, 20.5, 20.7, 107.4, 117.2, 120.68, 120.74, 124.6, 125.3, 126.0, 126.1, 126.9, 128.15, 128.20, 129.5, 134.3, 134.5, 137.3, 139.9, 142.1, 153.6 ppm. IR (ATR solid) 1/λ: 3389, 1488, 1447, 1411 cm^−1^. Elem. Anal. for C_21_H_17_NOS_2_: Calcd. C 69.39, H 4.71, N 3.85; found C 69.48, H 4.61, N 3.86.

**1‐hydroxy‐3,7,11‐trimethyl[1,4]benzothiazino[2,3,4‐kl]phenothiazine (7)**: Following the general procedure from **4** (80 mg, 0.21 mmol) and 3 eq. of BBr_3_ kept for 10 min at 0 °C and 4 h at rt. The crude was purified by flash chromatography on silica gel (Petroleum Ether/DCM: 1/3) to afford helicene **7** (59 mg, 78 % yield) as a white solid. M.p. 172.9‐175.9 °C. ^1^H NMR (400 MHz, CDCl_3_) δ: 2.23 (s, 3H), 2.277 (s, 3H), 2.281 (s, 3H), 6.64 (bs, 1H), 6.65 (bs, 1H), 6.81‐6.83 (m, 2H), 6.88 (bs, 1H), 6.94 (dd, 1H, *J*=8.2 Hz, *J*=1.3 Hz), 7.10 (d, 1H, *J*=1.4 Hz), ppm. ^13^C NMR (100 MHz, CDCl_3_) δ: 20.5, 20.6, 20.8, 116.5, 117.2, 120.2, 123.8, 125.8, 125.9, 126.1, 126.7, 127.2, 128.4, 128.5, 130.2, 134.5, 134.8, 136.7, 138.3, 140.2, 147.6, ppm. IR (ATR solid) 1/λ: 1299, 1449, 1485, 2851, 2918, 3018, 3414, 3538 cm^−1^. ESI‐MS negative mode, *m/z*=362 [M−H]^−^. Elem. Anal. for C_21_H_17_NOS_2_: Calcd. C 69.39, H 4.71, N 3.85; found C 69.27, H 4.55, N 3.79.

### Synthesis of Helicenes 8 and 9 by AlCl_3_ Mediated Cyclization of the Corresponding N‐thiophthalimide Derivatives (see the SI section for the preparation of the precursors)

**3‐hydroxy‐2‐methoxy‐7,11‐dimethyl[1,4]benzothiazino[2,3,4‐kl]phenothiazine** (**8**): To a solution of 10‐(4‐hydroxy‐5‐methoxy‐2‐N‐thiophthalimide‐phenyl)‐3,7‐dimethyl‐phenothiazine (40 mg, 0.076 mmol) in dry DCM (2.5 mL), AlCl_3_ (15 mg, 0.11 mmol) was added. The purple reaction mixture was stirred under a nitrogen atmosphere for 2 hours at room temperature. The mixture was diluted with DCM (30 mL), washed with NaHCO_3_ (15 mL×2) and with H_2_O (15 mL). The organic layer was collected, dried over Na_2_SO_4_, filtered, and then evaporated under reduced pressure. The crude was purified by flash chromatography on silica gel (DCM) to afford helicene **8** (17 mg, 58 % yield) as a white solid. M.p. 220 °C (dec). ^1^H NMR (400 MHz, CD_2_Cl_2_) δ: 2.21 (s, 3H), 2.28 (s, 3H), 3.72 (s, 3H), 5.51 (s, 1H), 6.69‐6.73 (m, 2H), 6.79 (bs, 2H), 6.93–7.02 (m, 3H) ppm. ^13^C NMR (100 MHz, (CD_3_)_2_CO)) δ: 20.3, 20.5, 56.6, 106.1, 114.3, 118.3, 120.5, 126.38, 126.40, 126.66, 126.68, 126.8, 128.9, 129.2, 134.8, 135.0, 135.6, 138.4, 141.5, 145.2, 148.8, ppm. IR (ATR solid) 1/λ: 1246, 1445, 1487, 2852, 2919, 3419 cm^−1^. ESI‐MS negative mode, *m/z*=378 [M−H]^−^. Elem. Anal. for C_21_H_17_NO_2_S_2_: Calcd. C 66.47, H 4.52, N 3.69; found C 66.37, H 4.50, N 3.73.

**3‐hydroxy‐2‐methoxy‐4,7,11‐trimethylbenzo[1,4]benzothiazino[2,3,4‐kl]phenothiazine (9)**: To a solution of 10‐(4‐hydroxy‐5‐methoxy‐3‐methyl‐2‐N‐thiophthalimidephenyl)‐3,7‐dimethyl‐phenothiazine (190 mg, 0,17 mmol) in dry DCM (5 mL), AlCl_3_ was added (34 mg, 0,25 mmol). The purple reaction mixture was stirred at room temperature under a nitrogen atmosphere for 4 hours. The solution was diluted in DCM (60 mL), washed with a saturated solution of NaHCO_3_ (2×20 mL) and H_2_O (20 mL). Then, the solution was dried over MgSO_4_, filtered, and evaporated under reduced pressure. The crude was purified by flash chromatography on silica gel (DCM/Petroleum Ether: 1/1) to obtain **9** (40 mg, 49 % yield) as a grey solid. M.p. 177–180 °C. ^1^H NMR (400 MHz, (CD_3_)_2_CO)) δ: 2.21 (bs, 3H), 2.25 (bs, 3H), 2.26 (bs, 3H), 3.68 (s, 3H), 6.64 (s, 1H), 6.84‐6.85 (m, 1H), 6.87–6.88 (m, 1H), 6.96–7.02 (m, 2H), 7.04 (bs, 1H), ppm. ^13^C NMR (100 MHz, (CD3)2CO)) δ: 12.7, 20.3, 20.5, 56.6, 103.5, 119.4, 120.7, 122.1, 126.17, 126.21, 126.68, 126.71, 126.9, 128.8, 129.1, 134.4, 134.7, 135.5, 138.6, 141.8, 142.9, 147.5, ppm. IR (ATR solid) 1/λ: 3422, 2941, 1474, 1467, 1309, 1256, cm^−1^. ESI‐MS negative mode, *m/z*=392 [M−H]^−^ Elem. Anal. for C_22_H_19_NO_2_S_2_: calcd. C 67.15, H 4.87, N 3.56; found C 66.95, H 4.77, N 3.66.

## Conflict of interest

The authors declare no conflict of interest.

## Supporting information

As a service to our authors and readers, this journal provides supporting information supplied by the authors. Such materials are peer reviewed and may be re‐organized for online delivery, but are not copy‐edited or typeset. Technical support issues arising from supporting information (other than missing files) should be addressed to the authors.

Supplementary
