# Supplementary material for: SET and HAT/PCET acid‐mediated oxidation processes in helical shaped fused bis‐phenothiazines
Source: Chemphyschem. 2021 Jun 17;22(14):1446–54. doi: 10.1002/cphc.202100387 (PMC8361695; doi:10.1002/cphc.202100387)
Supplement: Supplementary file 1 — Supplementary [file CPHC-22-1446-s001.pdf]

ChemPhysChem

Supporting Information

SET and HAT/PCET acid-mediated oxidation processes in helical shaped fused bis-phenothiazines

Riccardo Amorati,* Luca Valgimigli, Andrea Baschieri, Yafang Guo, Fabio Mollica, Stefano Menichetti, Michela Lupi, and Caterina Viglianisi*

Supplementary Information

A Graphical summary of radical species generated, and technique used *Page 1*

*Synthesis of the precursors of helicenes **8** and **9*** *Page 1-2*

Cartesian coordinates of the optimized structures *Page 3-37*

^{1}H and ^{13}C spectra of new compounds *Page. 38-46*

Calculation of the splitting constants *Page 47-49*

Supplementary Information references *Page 49*

Table S1 Graphical summary of radical species generated, and technique used.

Generation technique	*Radical intermediate formed*	*Substrate*
TFA, under air	$Hel^{•+}$	**1-9**
UV light 10% PhCl, under N_2	$Hel^{•+}$	**1-9**
AcOH, $ROO^•$, under air	$Hel^{•+}$	**1-9**
$TBPN^{•+}$	$Hel^{•+}$	**1-4**
$ROO^•$, under air	Hel-$O^•$	**5-9**
$^tBuOO^tBu$, UV light, under N_2	Hel-$O^•$	**5-9**

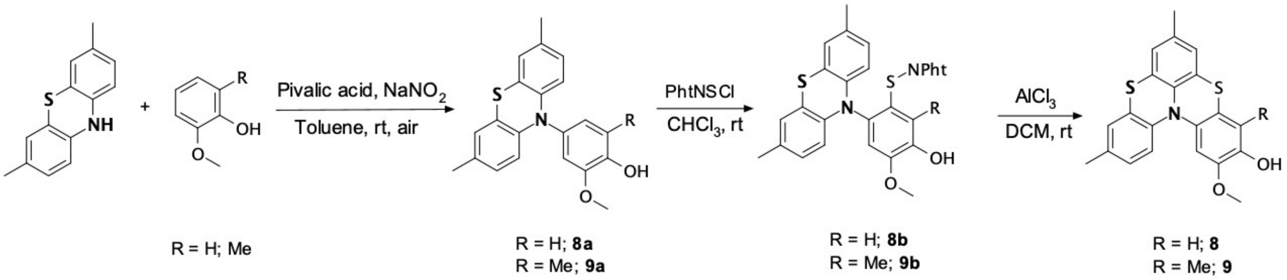

Figure 1SI. Synthetic procedure for the preparation of helicenes **8** and **9**

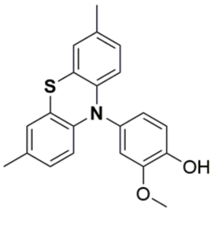

10-(4-hydroxy-3-methoxyphenyl)-3,7-dimethyl-phenothiazine:[1SI] To a solution of 3,7-dimethyl-10H-phenothiazine (300 mg, 1.31 mmol) and guaiacol (487 mg, 3.93 mmol) in 21 mL of dry Toluene, Pivalic acid (4.6 g, 45 mmol) was added. Then, $NaNO_2$ (7 mg, 0.10 mmol) was added under vigorous stirring. The reaction mixture was stirred at room temperature under air atmosphere for 18 h. Then the mixture was diluted with DCM (150 mL), washed with a saturated solution of Na_2CO_3 (60 mL × 4), then with water (50 mL). The organic layer was collected, dried over Na_2SO_4, filtered and evaporated under reduced pressure. The crude was purified by flash chromatography column on silica gel (Petroleum Ether/AcOEt: 5/1) to afford **8a** (306 mg, 67% yield) as a yellowish solid. M.p. 49-55 °C. ^{1}H NMR (400 MHz, $CDCl_3$) δ: 2.18 (s, 6H), 3.88 (s, 3H), 5.78 (bs, 1H), 6.13 (d, 2H, *J*=8.4 Hz), 6.64 (dd, 2H, *J*=2.1 Hz, *J*=8.4 Hz), 6.82 (d, 2H, *J*=1.8 Hz), 6.86 (d, 1H, *J*=2.2 Hz), 6.90 (dd, 1H, *J*=2.3 Hz, *J*= 8.3 Hz), 7.12 (d, 1H, *J*=8.3 Hz), ppm. ^{13}C NMR (100 MHz, $CDCl_3$) δ: 20.2, 56.2, 113.2, 115.4, 115.7, 119.4, 124.4, 127.1, 127.4, 131.6, 133.3, 142.4, 145.4, 148.3, ppm. IR (ATR solid) 1/λ: 3505, 2918, 2851, 1500, 1471, 1240 cm^{-1}. Elem. Anal. for $C_{21}H_{19}NO_2S$: Calcd. C 72.18, H 5.48, N 4.01; found C 72.27, H 5.46, N 4.02.

10-(4-hydroxy-3-methoxy-5-methylphenyl)-3,7-dimethyl-phenothiazine:[1SI] To a solution of 3,7-dimethyl-10H-phenothiazine (83 mg, 0.36 mmol) and 2-methoxy-6-methylphenol (200 mg, 1.44 mmol) in 5.6 mL of dry Toluene, Pivalic acid (1.25 g, 12 mmol) was added. Then, $NaNO_2$ (2 mg, 0.03 mmol) was added under

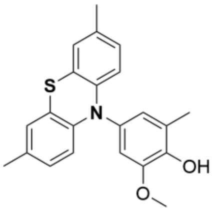

vigorous stirring. The reaction mixture was stirred at room temperature under an air atmosphere for 72 h. Then the mixture is diluted with DCM (80 mL), washed with a saturated solution of Na_2CO_3 (50 mL × 2) and water (50 mL). The organic layer was collected, dried over Na_2SO_4, filtered and evaporated under reduced pressure. The crude was purified by flash chromatography column on silica gel (Petroleum Ether/AcOEt: 10/1) to afford **9a** (93 mg, 71% yield) as a colorless oil. ^{1}H NMR (400 MHz, $CDCl_3$) δ: 2.17 (s, 6H), 2.32 (s, 3H), 3.86 (s, 3H), 5.81 (s, 1H), 6.14 (d, 2H, *J*=8.4 Hz), 6.63 (dd, 2H, *J*=2.0 Hz, *J*=8.4 Hz), 6.71 (d, 1H, *J*=2.2 Hz), 6.78 (d, 1H, *J*=2.0 Hz), 6.80 (d, 2H, *J*=2.2 Hz), ppm. ^{13}C NMR (100 MHz, $CDCl_3$) δ: 15.7, 20.3, 56.3, 110.7, 115.4, 119.2, 125.5, 125.8, 127.0, 127.3, 131.5, 132.3, 142.4, 143.5, 147.8, ppm. IR (ATR solid) 1/λ: 3512, 2919, 1471, 1240 cm^{-1}. Elem. Anal. for $C_{22}H_{21}NO_2S$: Calcd. C 72.70, H 5.82, N 3.85; found C 72.57, H 5.76, N 3.78.

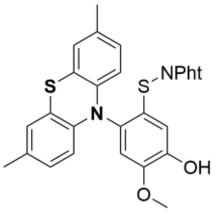

10-(4-hydroxy-5-methoxy-2-N-thiophthalimide-phenyl)-3,7-dimethyl-phenothiazine: To a solution of 10-(4-hydroxy-3-methoxyphenyl)-3,7-dimethyl-phenothiazine (60 mg, 0.17 mmol) in dry $CHCl_3$ (1 mL) a solution of PhtNSCl (47 mg, 0.20 mmol) in dry $CHCl_3$ (1 mL) was added. The mixture was stirred for 1h at room temperature, then was diluted with DCM (50 mL), washed with a saturated solution of Na_2CO_3 (30mL×2), then with water (30 mL). The organic layer was dried over Na_2SO_4, filtered and evaporated under reduced pressure. The crude was purified by flash chromatography on silica gel (DCM) to obtain **8b** (53 mg, 56% yield) as an orange solid. M.p. 160-164 °C. ^{1}H NMR (400 MHz, $CDCl_3$) δ: 2.10, (s, 6H), 3.82 (s, 3H), 5.88 (s, 1H), 6.02 (d, 2H, *J*=8.3 Hz), 6.57 (dd, 2H, *J*=8.4 Hz, *J*= 2.0 Hz), 6.70 (d, 2H, *J*= 1.7 Hz), 6.82 (s, 1H), 6.93 (s, 1H), 7.71-7.84 (m, 4H), ppm. ^{13}C NMR (100 MHz, $CDCl_3$) δ: 20.2, 56.5, 114.0, 114.2, 115.2, 119.0, 123.9, 126.9, 127.4, 129.8, 129.9, 131.9, 132.0, 134.5, 139.9, 146.3, 147.8, 167.4, ppm. IR (ATR solid) 1/λ: 3518, 2917, 1730, 1710, 1478, 1279, 1259 cm^{-1}. Elem. Anal. for $C_{29}H_{22}N_2O_4S_2$: Calcd. C 66.14, H 4.21, N 5.32; found C 66.24, H 4.19, N 5.30.

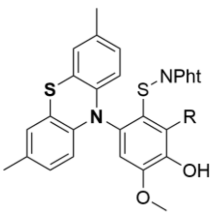

10-(4-hydroxy-5-methoxy-3-methyl-2-N-thiophthalimidephenyl)-3,7-dimethyl-phenothiazine: To a solution of 10-(4-hydroxy-3-methoxy-5-methylphenyl)-3,7-dimethyl-phenothiazine (56 mg, 0.15 mmol) in dry $CHCl_3$ (1 mL) a solution of PhtNSCl (36 mg, 0.17 mmol) in dry $CHCl_3$ (0.5 mL) was added. The mixture was stirred for 3 h, diluted with DCM (50 mL), washed with a saturated solution of Na_2CO_3 (30mL×2), then with water (30 mL). The organic layer was dried over Na_2SO_4, filtered and evaporated under reduced pressure. The crude was purified by flash chromatography on silica gel (DCM) to obtain **9b** (40 mg, 49% yield) as an orange solid. M.p. 220 °C (dec). ^{1}H NMR (400 MHz, $CDCl_3$) δ: 1.87 (s, 6H), 2.84 (s, 3H), 3.80 (s, 3H), 5.46 (d, 2H, J=8.4 Hz), 5.96 (s, 1H), 6.11 (dd, 2H, J=1.9 Hz, J=8.4 Hz), 6.45 (d, 2H, J=1.8 Hz), 6.59 (s, 1H), 7.53-7.60 (m, 4H), ppm. ^{13}C NMR (100 MHz, $CDCl_3$) δ: 14.5, 20.0, 56.3, 111.5, 114.3, 117.5, 123.1, 126.3, 126.8, 130.91, 130.92, 132.1, 133.4 (2C), 135.2, 139.4, 143.9, 149.5, 166.9, ppm. IR (ATR solid) 1/λ: 3517, 2919, 1729, 1709, 1476, 1278, 1248 cm^{-1}. Elem. Anal. for $C_{30}H_{24}N_4O_2S_2$: Calcd. C 66.65, H 4.47, N 5.18; found C 66.55, H 4.44, N 5.11.

Cartesian coordinates of the optimized structures, at the indicated level of theory.

CH_3OO^-

b3lyp/6-31+g(d,p) scrf=(solvent=acetonitrile)

C,-0.003065246,-0.005529136,-0.0050842651\O,-0.0075385363,0.0135986647,1.3981488763\

H,1.0230276792,0.002950662,-0.4090907644\H,-0.5395057089,0.8692111766,-0.4090902759\

H,-0.5115241029,-0.9 226709867,-0.3336889075\O,0.6758262935,1.2190332187,1.8589256171\\

$CH_3OO^{\bullet}$

b3lyp/6-31+g(d,p) scrf=(solvent=acetonitrile)

C, 0.0031331146,0.0056513649,-0.0464274108\O,0.02926 56431,0.0527878647,1.4095613109\

H,1.0300416016,-0.0002748863,-0.4148227946\H,-0.5459575931,0.8734507655,-0.414822462\

H,-0.512068982,-0.92365 4069,-0.2892877536\O,0.6328066017,1.141435226,1.8559193906\\

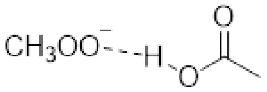

b3lyp/6-31+g(d,p) scrf=(solvent=acetonitrile)

C,-0.0003086091,-0.0014891448,0.001495539\O,0.0050777886, -0.0008341384,1.4239580562\

O,1.3970082268,0.0020236555,1.8544986743\H,0.5185856339,-0.8825499514,-0.3938932065\

H,0.4552619376,0.9116125623,-0.3997499993\H,-1.0582945052,-0.040129476,-0.2774235508\

H, 1.5330384136,0.9770417677,2.130372365\O,1.8754186316,2.4662218472,2.4588023466\

C,1.5823050502,3.0550412068,3.5593263759\O,1.870619 2151,4.2484928136,3.8260104264\

C,0.8354936314,2.2219114955,4.6074616793\H,-0.074025181,1.7944042102,4.1727979661\

H,1.4630532108,1.3830353622,4.9287266109\H,0.5720619689,2.8233462489,5.4797727392\\

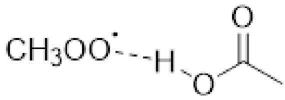

b3lyp/6-31+g(d,p) scrf=(solvent=acetonitrile)

C, 0.1557205178,0.1970416485,-0.0652600462\O,0.3668060817,-0.2171091869,1.3173556598\

O,0.8253009956,0.7751305244,2.0583927417\O,1.3772018921,-0.2540086342,4.5863511409\

C,0.4319192741,-0.0978758666,5.5314898364\C,-0.8490543821,0.5804785875,5.1037367478\

O,0.6437108758,-0.5068235265,6.6608503245\H,1.1113848457,0.5212778908,-0.4787844331\

H,-0.5800297735,1.0020268687,-0.0766459362\H,-0.2170909826,-0.6953366593,-0.5664693646\

H,1.1083284668,0.1161895334,3.7118758833\H,-1.3606527956,-0.0278019859,4.3505590586\

H,-1.501540734,0.7082840638,5.9668320159\H,-0.6379607751,1.5562826107,4.6555494699\\

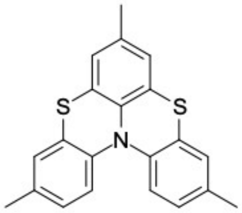

1

b3lyp/6-31+g(d,p) scrf=(solvent=acetonitrile)

C,0.0769581893,0.0763252188,-0.0531853539\C,0.0213400629,-0.0225377592,1.3457453967\

C,1.2280703469,-0.0904416184,2.0583558185\C,2.4555472611,-0.063210773,1.3852284467\

C,2.5149550109,-0.0084360205,-0.0122085721\C,1.3016490755,0.0636723593,-0.7171004988\

S,1.1917109486,-0.2607805868,3.8376795744\C,-0.2627447336,0.71898693,4.1790079124\

C,-1.3210923594,0.7134393271,3.2573315746\N,-1.2192090828,-0.0023480259,2.0373787761\

C,-2.4843341098,1.4293050579,3.5762616216\C,-2.5957242294,2.1065465102,4.7950135799\

C,-1.5272994323,2.1403167363,5.7007312849\C,-0.3555017708,1.4459814083,5.3693430018\

C,-2.3578214932,-0.6885807785,1.5371825097\C,-3.6375301363,-0.1297635952,1.6746684397\

S,-3.8142314502,1.5011413386,2.3856575785\C,-4.7692448509,-0.8218338845,1.2267054835\

C,-4.6554947236,-2.0660776667,0.5955396707\C,-3.3706462899,-2.6196521194,0.4660117345\

C,-2.2431444947,-1.9554412703,0.9440105299\C,-1.6236841886,2.92579952,6.9884427618\

C,-5.8726384318,-2.7877240812,0.0652204349\C,3.8391635884,-0.0319905456,-0.7397706756\

H,-3.5205480353,2.6260083288,5.0292860962\H,0.4889529256,1.4529970266,6.0528130345\

H,-1.263696451,-2.4134510644,0.8531025984\H,-3.2502982842,-3.5945393993,0.0009564367\

H,-5.7497424365,-0.3736474777,1.36343931\H,3.3749319943,-0.0977632954,1.963558443\

H,1.3138586246,0.1300853571,-1.8018116243\H,-0.8463064243,0.1555970412,-0.6177216154\

H,-5.8390396322,-3.854792894,0.3085855608\H,-6.7950155405,-2.3721226211,0.4803594766\

H,-5.9343287413,-2.7067097097,-1.0270126902\H,-1.0646497713,2.4395053169,7.7935202708\

H,-1.2094768774,3.9340889047,6.8650566266\H,-2.6632561199,3.034801822,7.3102762107\

H,3.8593661921,0.7025672964,-1.5515668834\H,4.0249375964,-1.0155149651,-1.1886532406\

H,4.6700623759,0.1849665059,-0.0627461243\\

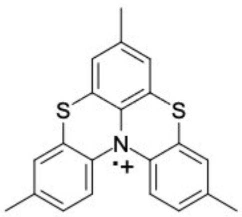

1•+

b3lyp/6-31+g(d,p) scrf=(solvent=acetonitrile)

C,-0.073636503,-0.0696529147,0.0590788396\C,-0.0188878704,0.0286659625,1.4651705505\
C,1.2416477682,0.0935073167,2.092824154\C,2.4110172808,-0.012583824,1.3278708104\
C,2.358762973,-0.1528927039,-0.0594735566\C,1.0911196087,-0.1586241501,-0.6814843698\
S,1.3852395284,0.2645114041,3.8398241573\C,-0.1374497081,1.0882685673,4.1839026795\
C,-1.2834031923,0.8866664364,3.3755350743\N,-1.21689174,0.0961756425,2.2178634641\
C,-2.4969406584,1.5011375886,3.7674670184\C,-2.5509838701,2.282667925,4.9221055051\
C,-1.4160262976,2.4858883063,5.7160549214\C,-0.2139498326,1.8767103137,5.330410272\
C,-2.3460699891,-0.6416490593,1.7876017609\C,-3.6544817884,-0.1532775797,1.9811147477\
S,-3.9471171445,1.3939943493,2.7688084445\C,-4.752599288,-0.8870441184,1.5121970309\
C,-4.5833993381,-2.1178522935,0.8773365104\C,-3.2715586981,-2.6225607915,0.7409277016\
C,-2.1753366868,-1.9054783009,1.1843517211\C,-1.4748672487,3.3568999384,6.9440919073\
C,-5.7627207948,-2.9090161853,0.3758993674\C,3.6150313412,-0.2650171231,-0.8826420186\
H,-3.4944024652,2.7379983413,5.2061981019\H,0.6775551657,2.0180176904,5.9336005931\
H,-1.1800549445,-2.3219016098,1.0871340974\H,-3.1167332653,-3.5990322628,0.2924886267\
H,-5.752105216,-0.4915688561,1.6646626233\H,3.3728144457,0.0327672941,1.8294920396\
H,1.0247458195,-0.2194672344,-1.7633675106\H,-1.0343002989,-0.0558359818,-0.4413661402\
H,-5.7873514073,-3.9034606274,0.8349725791\H,-6.7071297696,-2.4055229437,0.5937885268\
H,-5.6944088142,-3.0571493979,-0.7078220981\H,-0.865306622,2.9428878035,7.752234628\
H,-1.0862155148,4.3576984994,6.7200192118\H,-2.5008398808,3.470584745,7.3021001961\
H,3.6548088091,0.5241042253,-1.6415027221\H,3.6430759772,-1.2232384708,-1.4138511317\
H,4.5106792046,-0.191684295,-0.261867894\\

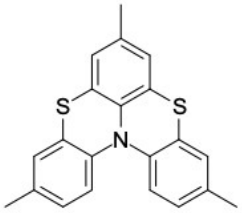

1

b3lyp/6-31+g(d,p)

C,-0.00867 25788,-0.0335142122,0.028339946\C,-0.0082120229,-0.0161362417,1.4311821094\

C,1.2256909054,0.0100135611,2.0988152045\C,2.4236995797,0.0157781157,1.3753404051\

C,2.4287979524,-0.0462205826,-0.0222018736\C,1.1892214814,-0.0682723044,-0.6810264131\

S,1.2634844718,-0.0133858235,3.8838214598\C,-0.2060058914,0.9488421266,4.2006092269\

C,-1.2985810711,0.8383465315,3.3279667233\N,-1.2210610681,0.0272867032,2.1671830092\

C,-2.4711988544,1.5441573547,3.6331557423\C,-2.556227096,2.3149679456,4.7964632415\

C,-1.4554479069,2.4544414521,5.650662057\C,-0.2764782428,1.7700594933,5.3293986455\

C,-2.3556494008,-0.7274551435,1.7703551945\C,-3.6455090791,-0.1941861311,1.9153559863\

S,-3.8467109687,1.481736841,2.4977583937\C,-4.7697813243,-0.952952003,1.5706863401\

C,-4.6421336755,-2.2393510893,1.0359243502\C,-3.3478154077,-2.7653435562,0.8980986837\

C,-2.2246046945,-2.0338980647,1.2760356748\C,-1.527738053,3.3428446793,6.871565096\

C,-5.8563643978,-3.0343338118,0.6146270791\C,3.7251563534,-0.0934261047,-0.7975067984\

H,-3.4894075303,2.8216561259,5.0263142195\H,0.5952500178,1.8598105007,5.9717280893\

H,-1.2347201931,-2.4689225821,1.1857574371\H,-3.2156496522,-3.7716785346,0.5087678264\

H,-5.7574329469,-0.523145608,1.7157996815\H,3.3642590445,0.060204045,1.9181132426\

H,1.1586633801,-0.0916084472,-1.7674326248\H,-0.9559683811,-0.0251171504,-0.5006919072\

H,-5.7700518027,-4.0836330753,0.9162953999\H,-6.771427033,-2.6306594784,1.0577748762\

H,-5.9828533497,-3.0187556744,-0.4752484773\H,-0.8866278485,2.9685528256,7.6756310591\

H,-1.1989453238,4.3633212447,6.6379205921\H,-2.5498535235,3.4080310073,7.2566959076\

H,3.6859158334,0.5548401069,-1.6794101227\H,3.940053721,-1.1098685573,-1.150610926\

H,4.5706574625,0.2266274233,-0.1816245903\\

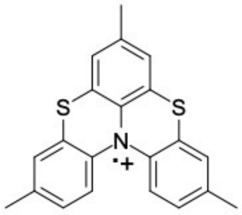

1•+

b3lyp/6-31+g(d,p)

C,-0.077488754,-0.0969292801,0.0811871069\C,-0.0273078493,0.0045171659,1.4849093143\

C,1.2337572104,0.084592484,2.1156566041\C,2.402560845,-0.0061391919,1.3536340728\

C,2.3567441338,-0.1484952284,-0.0372495113\C,1.0920183493,-0.1728270217,-0.6585424006\

S,1.3688553765,0.2499395557,3.8654300256\C,-0.1554921916,1.0750902595,4.1998957021\

C,-1.2997216106,0.8623096985,3.3919557684\N,-1.2273619808,0.064234854,2.2382756415\

C,-2.518113653,1.4717253358,3.7794247407\C,-2.5772747044,2.2595652949,4.9290979096\

C,-1.4442354898,2.4756271298,5.7239626119\C,-0.23889144,1.8704699918,5.3416694294\

C,-2.3521512938,-0.6846599445,1.8131144773\C,-3.6641015869,-0.2038653363,2.0070370501\

S,-3.964882552,1.3508334434,2.7770890843\C,-4.7582727361,-0.9495958921,1.5477915397\

C,-4.5848168181,-2.1849236641,0.9225282706\C,-3.2686950599,-2.6800411861,0.7845800261\

C,-2.1757171456,-1.9518367334,1.2175971691\C,-1.5116411361,3.3558202713,6.9447407914\

C,-5.7606030719,-2.9900304096,0.4364148154\C,3.627882252,-0.2366863496,-0.841390664\

H,-3.5247225304,2.7088672012,5.2108429078\H,0.6519612706,2.0213655015,5.9439354134\

H,-1.1773550717,-2.3617960311,1.1198942907\H,-3.1093382826,-3.6600768784,0.3448597478\

H,-5.760367476,-0.5609989996,1.7030963694\H,3.3635685201,0.0552163958,1.8566805531\

H,1.0284277606,-0.2359650025,-1.740304754\H,-1.0373584498,-0.096011017,-0.4219093652\

H,-5.7856998595,-3.9737225094,0.9182636264\H,-6.7081302011,-2.4868002577,0.6403554409\

H,-5.6923409412,-3.162567122,-0.6436861851\H,-0.8189885673,3.0176253968,7.71991913\

H,-1.2399878938,4.3870398439,6.6873868397\H,-2.5194419867,3.3768097243,7.3670391203\

H,3.4202760824,-0.3336936349,-1.9091688838\H,4.2273119851,-1.099892315,-0.5316515167\

H,4.2468574327,0.6555583643,-0.6959731435\\

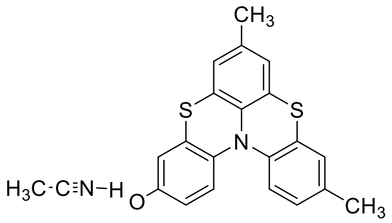

5

b3lyp/6-31+g(d,p) scrf=(solvent=acetonitrile)

C,-0.1660695895,0.0604501492,-0.0241026274\C,-0.1510832431,-0.0304200488,1.3744135782\

C,1.0902457205,-0.1501084952,2.0203227999\C,2.2804903621,-0.1876230426,1.2894606936\

C,2.2458558441,-0.1433197471,-0.1089382503\C,1.0121239483,-0.0169275194,-0.7654246423

S,1.1455485627,-0.2905330514,3.8010109323\C,-0.2511069352,0.7479487615,4.2065753464\

C,-1.3613838217,0.771442105,3.3485003668\N,-1.3544781928,0.0473937318,2.131118703\

C,-2.4842294624,1.5186734974,3.7348392646\C,-2.5021955407,2.2066395644,4.9519762345\

C,-1.3784418567,2.2176512362,5.7895064103\C,-0.2504938709,1.4855072235,5.3945665056\

C,-2.5331582079,-0.6281683842,1.7208939043\C,-3.7907457285,-0.0432852118,1.9388630334

S,-3.8842203085,1.6045908442,2.6279683779\C,-4.9635014796,-0.7205151218,1.5848517915\

C,-4.9171740438,-1.9760590207,0.966839376\C,-3.654724514,-2.553774956,0.752833419\

C,-2.4836459971,-1.9041019154,1.138084452\C,-1.3737284399,3.0180111909,7.0716543218\

C,-6.1819028187,-2.6810427929,0.5341675784\O,3.4350630122,-0.2127339407,-0.772065705\

H,-3.396680121,2.752165104,5.2390765442\H,0.632626251,1.4701405264,6.0271071384\

H,-1.5215575393,-2.3809894078,0.9824624449\H,-3.5853272979,-3.5369421356,0.2947663916\

H,-5.9235511479,-0.2517101422,1.7838768511\H,3.2371471517,-0.2604332919,1.7968078796\

H,0.974757314,0.0382747548,-1.8486670931\H,-1.1141146348,0.1781855942,-0.5387003768\

H,3.3103545964,-0.156831345,-1.7436671317\H,-6.1274744133,-3.7552213265,0.7386825531\

H,-7.0588461394,-2.2794336868,1.0497327244\H,-6.3499013407,-2.5644452963,-0.5437349811\

H,-0.7207721919,2.5628792339,7.8221221262\H,-1.0108396001,4.038334148,6.8961779587\

H,-2.3795144745,3.096578221,7.4946027556\N,3.3065988869,-0.0591535705,-3.6188481206\

C,3.3409602136,0.0304488367,-4.7748476578\C,3.3846114118,0.1433324507,-6.2267768588\

H,2.3693723077,0.1136767187,-6.6305210231\H,3.8560329314,1.0878468883,-6.5099151695\

H,3.9614161005,-0.6855294139,-6.6447947002

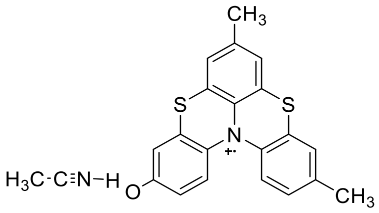

5•+

b3lyp/6-31+g(d,p) scrf=(solvent=acetonitrile)

C,0.005528003,0.038697388,0.0023416483\C,0.0055593259,0.0312043523,1.41503527\

C,1.2496288416,0.0410734136,2.0936501462\C,2.4421848839,-0.0043221226,1.3774041229\

C,2.4242314311,-0.0390604929,-0.0238896633\C,1.1878266304,0.0039354737,-0.7093302022\

S,1.3230101382,0.030820343,3.8547070291\C,-0.2124363301,0.8234779872,4.2226676651\

C,-1.3201539185,0.720137954,3.3519508764\N,-1.2118107628,0.0443910914,2.1218468847\

C,-2.5413605803,1.3159310512,3.7432347121\C,-2.6418750383,1.9756726775,4.9677761261\

C,-1.5431082666,2.0776403548,5.8314993942\C,-0.3319370553,1.4941206822,5.4405489194\

C,-2.3328651744,-0.6449143089,1.590932194\C,-3.6400816145,-0.1520537052,1.7678187085\

S,-3.9401766821,1.3388295322,2.6623416707\C,-4.7264433859,-0.8345152512,1.2053650885\

C,-4.5460883873,-2.018238468,0.4871117754\C,-3.2373268264,-2.5313195426,0.3666123223\

C,-2.1515661722,-1.8646043922,0.9076343809\C,-1.6571073403,2.8227664187,7.1367565647\

C,-5.7148495283,-2.749034838,-0.120960989\O,3.6044030002,-0.0822481599,-0.6593630369\

H,-3.5920763194,2.4169802092,5.2518353283\H,0.5335904313,1.5625180216,6.0920948397\

H,-1.1575890255,-2.2875426115,0.8225184009\H,-3.0749518598,-3.4744644787,-0.1459927893\

H,-5.7269197468,-0.4376715154,1.3480198503\H,3.3964433271,0.0006496919,1.892685623\

H,1.1695819441,0.027745903,-1.7931244138\ H,-0.9362967394,0.095831555,-0.5297040399\

H,3.5067263771,-0.0945798607,-1.6436247959\H,-5.7506640762,-3.7867494474,0.2278979162\

H,-6.6633424739,-2.2684164872,0.1289885774\H,-5.6242157605,-2.7796650678,-1.2128729269\

H,-0.9129123603,2.4771348584,7.8585181011\H,-1.4950564266,3.8959935469,6.9790087301\

H,-2.6516884503,2.7026899524,7.5749953465\N,3.5127277091,-0.1422232304,-3.4365765532\

C,3.556111222,-0.2017191868,-4.5936213001\C,3.6105225417,-0.2772484689,-6.0469739439\

H,3.035533165,0.5446548656,-6.4810100182\H,4.6486195324,-0.2055421982,-6.3811740563\

H,3.1892531046,-1.2281651752,-6.3829079244

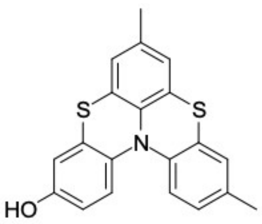

5

b3lyp/6-31+g(d,p)

C,-0.0115384406,0.1534451049,-0.0213546899\C,-0.0310888442,0.0352516983,1.3739013804\

C,1.1943017265,-0.0819077768,2.0530151151\C,2.4034030102,-0.0888240715,1.3540548892\

C,2.3999660742,-0.0179749378,-0.0403780641\C,1.1887333851,0.1044601069,-0.729562402\

S,1.2033357561,-0.2651361835,3.8276314707\C,-0.2158515536,0.7459510252,4.2169271153\

C,-1.3020297005,0.7783880107,3.3300940362\N,-1.2528135298,0.0795438395,2.0984728153\

C,-2.4419761935,1.5080117875,3.6963224597\C,-2.5004853052,2.1674127479,4.9276455998\

C,-1.4023377004,2.1695216301,5.7966682407\C,-0.2563866969,1.4581979832,5.4187972271\

C,-2.4150279839,-0.5882250844,1.6337532197\C,-3.6809319501,-0.0125846105,1.8248525855\

S,-3.807835422,1.613930211,2.5522490534\C,-4.8363088652,-0.6864272324,1.41382282\

C,-4.7638491981,-1.9244783105,0.7660386573\C,-3.4935787309,-2.4927524136,0.5812903831\

C,-2.3400844278,-1.8491826284,1.0231808604\C,-1.4415692867,2.9413685118,7.0958011224\

C,-6.0105657068,-2.6228506303,0.2742076229\O,3.6165064493,-0.0597909475,-0.6734075256\

H,-3.409767457,2.6957092625,5.2005339394\H,0.611082477,1.4402352549,6.0728177338\

H,-1.3703725109,-2.3186181725,0.894418089\H,-3.404759869,-3.4652050385,0.1035328281\

H,-5.8044164756,-0.2273933292,1.5964027268\H,3.3488544389,-0.1560804961,1.8816619668\

H,1.1786623993,0.1808709985,-1.8142294571\H,-0.9478637806,0.270810909,-0.5565164355\

H,3.4945929893,0.0237675398,-1.6279980009\H,-5.9639276404,-3.7007515478,0.4621086219\

H,-6.9067746982,-2.233684739,0.7662661243\H,-6.1424718441,-2.4865579147,-0.8066350384\

H,-0.8349163259,2.4560578174,7.8665960406\H,-1.0512752104,3.9583074838,6.9633334915\

H,-2.4638433958,3.0295464134,7.4758303713\\

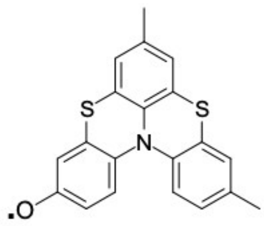

5(-H)•

b3lyp/6-31+g(d,p)

C,-0.0549312306,0.0377468518,-0.029690998\C,-0.0326722324,0.0330386365,1.3922107099\
C,1.228966651,0.01500222,2.0598017861\C,2.4033700014,0.0049679304,1.3444840133\
C,2.4106392539,-0.0116340361,-0.1093582151\C,1.1075922486,0.0130256754,-0.7570256372\
S,1.2749599278,-0.1546448242,3.836077358\C,-0.1863800192,0.7708803328,4.2458539827\
C,-1.2795680389,0.7712741999,3.3674637709\N,-1.2221284929,0.0800443819,2.1241385189\
C,-2.4324747596,1.4867284996,3.7254664144\C,-2.5020611528,2.1469642562,4.9555319168\
C,-1.4104296052,2.1564489918,5.8322818992\C,-0.2501566616,1.4707946249,5.4541830821\
C,-2.397042581,-0.5761194584,1.6524918488\C,-3.6583362879,0.0125907476,1.8213935461\
S,-3.7959604154,1.614401936,2.5853737961\C,-4.8086910713,-0.6480617903,1.3742487198\
C,-4.7316766925,-1.8902284407,0.7366283027\C,-3.4647343231,-2.4814785637,0.5971947234\
C,-2.3176648613,-1.8464918465,1.0616054102\C,-1.4700821495,2.9178389177,7.1359982324\
C,-5.9712248869,-2.5802288706,0.2185450279\O,3.4795428421,-0.0340359091,-0.7647936593\
H,-3.4170985938,2.667542341,5.2230052932\H,0.6182167195,1.4734798183,6.1068158178\
H,-1.3506059448,-2.328818154,0.9669463304\H,-3.3759058556,-3.4611649893,0.1356029703\
H,-5.7767447171,-0.1802175892,1.5308958974\H,3.365834634,-0.0119727044,1.8457094142\
H,1.0921766905,0.0372187735,-1.8418742364\H,-1.0148833704,0.0855419832,-0.5328953132\
H,-5.9810026689,-3.6399433228,0.4945571668\H,-6.8807175103,-2.1192541205,0.6135153747\
H,-6.0212284431,-2.529170778,-0.8760113387\H,-0.8462236683,2.4470878448,7.9015263857\
H,-1.1112366338,3.946384069,7.0061413424\H,-2.4935022645,2.9729466254,7.5185149798\\

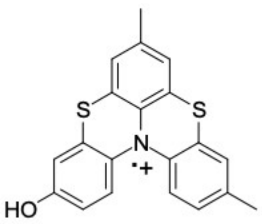

5•+

b3lyp/6-31+g(d,p)

C,-0.101711584,0.0464368068,0.0186026066\C,-0.0579813582,0.0322719842,1.4284613921\

C,1.2040569542,0.0027724877,2.0696399135\C,2.3737638141,-0.0756447505,1.3174593496\

C,2.3083546497,-0.1040702888,-0.079663386\C,1.0598144862,-0.0217425059,-0.7288424197\

S,1.3281231874,-0.0016622434,3.8282718457\C,-0.1580312499,0.8698943866,4.2255758599\

C,-1.3025385749,0.7842877349,3.3967950403\N,-1.2565730032,0.0855597792,2.1764026641\

C,-2.49052122,1.4219445032,3.8230181347\C,-2.5218061327,2.1153403548,5.0350130831\

C,-1.389406444,2.2048793953,5.8518442852\C,-0.2108022537,1.5721533813,5.4270341781\

C,-2.4133967184,-0.5718740831,1.6875557724\C,-3.7011259638,-0.0430187078,1.9125728258\

S,-3.9316734288,1.4582443774,2.8051630724\C,-4.8263710339,-0.6962257698,1.3917344849\

C,-4.7083422897,-1.8858843076,0.6716954709\C,-3.4173251008,-2.4331434452,0.5011045174\

C,-2.2937807108,-1.7959040816,0.996076178\C,-1.4210243752,2.9711762119,7.1487798248\

C,-5.918507859,-2.591488662,0.1192830284\O,3.4800351987,-0.1800875172,-0.7422633375\

H,-3.4476433873,2.5894631428,5.3458479759\H,0.6809909529,1.6284174645,6.0442906226\

H,-1.3158638491,-2.2470602951,0.874251601\H,-3.3024372613,-3.3829506394,-0.012625561\

H,-5.8096670863,-0.2725726812,1.5733671017\H,3.3454020531,-0.0970153798,1.7989011669\

H,1.0063076796,0.0081075548,-1.8132400522\H,-1.0570103237,0.1323216737,-0.4854936157\

H,3.3535606413,-0.1667870994,-1.7019129599\H,-5.995596758,-3.6073812993,0.5223114889\

H,-6.8417190393,-2.0595309653,0.3587756053\H,-5.8509292528,-2.682040938,-0.9707846442\

H,-0.9805605717,2.3876476735,7.9632096057\H,-0.8419802768,3.8982002104,7.0622654028\

H,-2.441292548,3.2393288082,7.4316688717\\

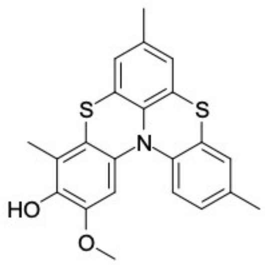

9

b3lyp/6-31+g(d,p)

C,-0.0567344134,0.0297093495,-0.016485841\C,-0.0317964665,0.011950751,1.38807583\
C,1.1965133026,0.0032740898,2.0593317018\C,2.4202149476,-0.0088349642,1.353577947\
C,2.3712998494,-0.0443323793,-0.0416433945\C,1.1383742701,-0.0205140068,-0.7213508708\
S,1.200989959,-0.0217640445,3.849916455\C,-0.2503479862,0.9710577082,4.1505583556\
C,-1.3374429127,0.8755852874,3.271019432\N,-1.2543936615,0.0625580034,2.1148727691\
C,-2.507979945,1.5858938872,3.571226877\C,-2.5935016027,2.3563765915,4.7345239697\
C,-1.4948320287,2.4872991977,5.5935687066\C,-0.3199894441,1.7919134201,5.2799250325\
C,-2.3836639831,-0.6973876696,1.7200841281\C,-3.6755484277,-0.1655710743,1.861450889\
S,-3.8790689354,1.5159913268,2.4298857281\C,-4.7982891583,-0.9284207093,1.5211985002\
C,-4.6685136024,-2.2171375844,0.9919382236\C,-3.372906444,-2.7401274743,0.8540453164\
C,-2.2505262223,-2.00501587,1.2281824651\C,-1.5662277088,3.3769116757,6.8137713684\
C,-5.8817017903,-3.0170291737,0.5765027632\O,3.5360730648,-0.0947850077,-0.7613634809\
H,-3.5243840292,2.8685457125,4.961661321\H,0.547386468,1.8711731592,5.929484325\
H,-1.2594779354,-2.4379345453,1.140195674\H,-3.2390891102,-3.7483408826,0.4698427825\
H,-5.7867634054,-0.4999008382,1.6648667991\C,3.753172899,-0.0134495082,2.060410614\
O,1.2566626026,-0.0362713101,-2.0918462251\H,-1.0105199892,0.073816852,-0.5263758196\
H,3.3016350812,-0.0810715377,-1.7021277599\H,-5.788951329,-4.0663472741,0.8762160345\
H,-6.7958319198,-2.6182810034,1.0260784075\H,-6.0158661981,-3.0008135354,-0.512546422\
H,-0.923764927,3.0038550238,7.6173178641\H,-1.2386598192,4.3975501142,6.578992525\
H,-2.5879048008,3.4416937376,7.2003328876\C,0.0690736864,0.0083313167,-2.8772189195\
H,0.3968196308,-0.0146454091,-3.9169982228\H,-0.4921983364,0.9313393279,-2.6894454099\
H,-0.5699722062,-0.8591937436,-2.6742926994\H,3.7674467989,0.7270862788,2.8661912998\
H,4.5604936267,0.2059862859,1.3608208307\H,3.9618997387,-0.9903744864,2.5145430616\\

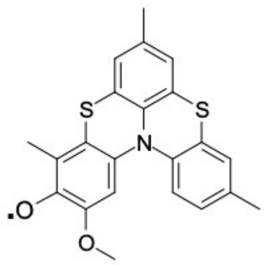

9(-H)•

b3lyp/6-31+g(d,p)

C,-0.0654640369,-0.0417452521,-0.0172534654\C,-0.0173687432,0.0397685679,1.3927600872\

C,1.2398202826,0.1004917235,2.0594820902\C,2.4327649092,0.0606716541,1.3562514845\

C,2.4205172819,-0.0538570681,-0.1027130672\C,1.1009881408,-0.0900934862,-0.7547112412\

S,1.2691912039,0.0782982137,3.8489215365\C,-0.2224484496,0.9849909356,4.1799401129\

C,-1.30718918,0.8796682368,3.3005267469\N,-1.2149242523,0.0893340955,2.1229730596\

C,-2.487170391,1.578761285,3.5970436085\C,-2.5889205175,2.3299168805,4.7706809236\

C,-1.5022757029,2.4488770111,5.6472029576\C,-0.3168111465,1.77727825,5.3288900435\

C,-2.3582293164,-0.6545525866,1.7107192789\C,-3.6436429292,-0.1068642819,1.8290677199\

S,-3.8450977229,1.554392277,2.4412197437\C,-4.7663668229,-0.8535323501,1.4516836375\

C,-4.6383549248,-2.1438767437,0.9283327193\C,-3.347220284,-2.6901195667,0.8345715837\

C,-2.2271355369,-1.9682601804,1.2347795768\C,-1.5990171937,3.3097690187,6.8855046832\

C,-5.8488914587,-2.9306869835,0.4838028427\O,3.472213351,-0.1093043157,-0.7745868284\

H,-3.5239231043,2.8360245163,4.9937384006\H,0.5469138467,1.8618331512,5.9822503228\

H,-1.2402497504,-2.4150899185,1.1778512743\H,-3.2176019671,-3.7031534705,0.462847942\

H,-5.7529108487,-0.4140268796,1.5711883047\C,3.7756596647,0.0899285956,2.0308460631\

O,1.1563210632,-0.1508350197,-2.0985490889\H,-1.0345911159,-0.0421290578,-0.4986180725\

H,-5.8160079479,-3.9581880058,0.8615582585\H,-6.7762809756,-2.4708838936,0.8364981049\

H,-5.9025475803,-2.9886693828,-0.6103145103\H,-0.894713853,2.9811260808,7.6552051999\

H,-1.3702277558,4.3576967539,6.6550164087\H,-2.6060502524,3.2810681077,7.3128597631\

C,-0.0610826213,-0.1799458217,-2.8366856915\H,0.2322416095,-0.2373119183,-3.8850335368\

H,-0.6477523201,0.7315257559,-2.6687072914\H,-0.6630767326,-1.0585313029,-2.5743353383\

H,3.8012273605,0.8389231977,2.8293247623\H,4.549310776,0.3069192128,1.2929463686\

H,4.0165799035,-0.8796564868,2.4870743294\\

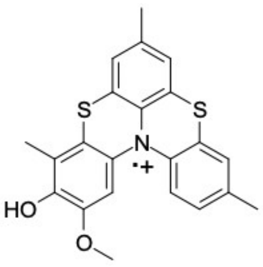

9•+

b3lyp/6-31+g(d,p)

C,-0.0435875672,-0.0831751966,0.0253542719\C,-0.0043786625,0.0678627091,1.4300101256\
C,1.2475915772,0.1511892451,2.0931623893\C,2.4595174693,0.0162061395,1.3866010255\
C,2.3916919193,-0.1607652728,0.0016714473\C,1.1381118062,-0.1883722076,-0.6794217852\
S,1.3334099824,0.3362018915,3.8428115237\C,-0.2121007456,1.1230303528,4.1591089214\
C,-1.3176371059,0.9362098051,3.3060600806\N,-1.2112786917,0.1450168015,2.1445606806\
C,-2.5389741325,1.5680795571,3.6361446993\C,-2.640708339,2.3301354953,4.7971055482\
C,-1.5435217304,2.5063715637,5.6563554516\C,-0.3316010131,1.8997168701,5.3159934374\
C,-2.3421099778,-0.6060183168,1.707375957\C,-3.6438709811,-0.0893158186,1.830498577\
S,-3.9220758544,1.5102318746,2.5304378943\C,-4.7387644653,-0.8398901476,1.382439548\
C,-4.571953483,-2.1120440226,0.8305640349\C,-3.265375974,-2.639040094,0.7639769092\
C,-2.1711649958,-1.9072565785,1.1969192011\C,-1.6814632247,3.3300751488,6.9112841728\
C,-5.751578858,-2.9197489481,0.3539541226\O,3.5266923658,-0.2807489693,-0.7087027864\
H,-3.5903016333,2.7996299663,5.0366891664\H,0.537428079,2.0359127568,5.9523156365\
H,-1.1783194357,-2.3411385897,1.1594213236\H,-3.1120236893,-3.6457353561,0.3862716399\
H,-5.7369984412,-0.4255455656,1.4883542795\C,3.7967334814,0.0643740768,2.0816842614\
O,1.2622882607,-0.3079892953,-2.0226490045\H,-0.9977446968,-0.0886081202,-0.4829645504\
H,3.3090063176,-0.3600570222,-1.6531587042\H,-5.8148748472,-3.8729873264,0.8902720404\
H,-6.6926618058,-2.3851429717,0.5006679686\H,-5.6580331089,-3.1553365079,-0.7121676128\
H,-0.7205585761,3.4591599217,7.4141935171\H,-2.0866321472,4.3225187523,6.6882685032\
H,-2.3689769725,2.8491447095,7.6164525958\C,0.0851497272,-0.3238187663,-2.8441382338\
H,0.4435932349,-0.4260483007,-3.8671519081\H,-0.469100494,0.6141226495,-2.7367835026\
H,-0.5508607844,-1.1768390315,-2.5864696406\H,3.8863253513,0.968296783,2.6938159731\
H,4.6098140995,0.0567370466,1.3566790846\H,3.9315665667,-0.7980393987,2.7452983053\\

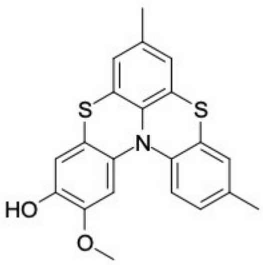

8

b3lyp/6-31+g(d,p)

C,-0.0183700844,0.0819507923,-0.0125312765\C,-0.0320812731,-0.0194773862,1.3907139955\
C,1.1803722531,-0.1199155552,2.0814260436\C,2.3964008927,-0.1275594839,1.3840164399\
C,2.406073675,-0.0775515839,-0.0031226932\C,1.1869531937,0.0281546191,-0.70375991\
S,1.1738219495,-0.270911092,3.8605432616\C,-0.2396678849,0.7605988048,4.2132582298\
C,-1.3179256484,0.7758710632,3.315648432\N,-1.2610928291,0.0449667835,2.1021015155\
C,-2.4587665191,1.5174586923,3.6524557214\C,-2.5274739973,2.2075081464,4.8664244766\
C,-1.4375570166,2.2270480404,5.7455199257\C,-0.2905409858,1.5025106686,5.3967115842\
C,-2.4195639377,-0.6334331806,1.6467986503\C,-3.6864063876,-0.0509577007,1.8122325614\
S,-3.8151251477,1.5950689896,2.4939508169\C,-4.8394121934,-0.7339163425,1.4097589975\
C,-4.7637894631,-1.9887314918,0.7950564802\C,-3.4928856566,-2.5630077058,0.6344564195\
C,-2.3416980017,-1.9096891809,1.0687078627\C,-1.4864695008,3.0306682223,7.0249599103\
C,-6.0080645238,-2.6987765414,0.3136302296\O,3.5926714181,-0.1169336418,-0.6779479244\
H,-3.4376954072,2.7448397196,5.1176663526\H,0.5701307333,1.4973921139,6.0598770272\
H,-1.371505428,-2.3837157437,0.9618448628\H,-3.4020690304,-3.5484036175,0.1842313895\
H,-5.8083381053,-0.2693121306,1.5730026643\H,3.3406740693,-0.1822859716,1.9152076943\
O,1.3300660615,0.0919808057,-2.0690553688\H,-0.958343302,0.1939187867,-0.5381217934\
H,3.4011291001,-0.048405513,-1.6260189935\H,-5.9628400051,-3.7717040521,0.5283865147\
H,-6.9067728807,-2.2974992789,0.7911845615\H,-6.1346049709,-2.5893901466,-0.7709592867\
H,-0.8927227135,2.5598827873,7.8146038302\H,-1.0867890271,4.0411644288,6.8726049536\
H,-2.5125029635,3.1356106901,7.390357323\C,0.1601208207,0.2290849576,-2.870467053\
H,0.5070029602,0.2572481655,-3.9038179628\H,-0.3704473326,1.1594745247,-2.6356843785\
H,-0.5138118982,-0.6246756215,-2.7323566072\\

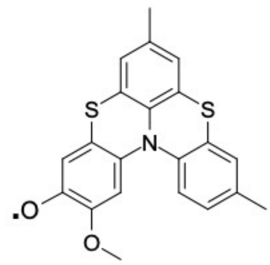

8(-H)•

b3lyp/6-31+g(d,p)

C,-0.0289135814,0.0333612269,-0.0190558048\C,-0.0132460636,0.0307426685,1.3954560024\

C,1.2350013942,0.011010839,2.0837106338\C,2.417396457,-0.007718985,1.3810428018\

C,2.4616660174,-0.0360078581,-0.065934321\C,1.149555431,-0.0042992062,-0.7431135404\

S,1.2562143011,-0.1440404876,3.8603438334\C,-0.2064566437,0.7910568905,4.2423704315\

C,-1.2862209541,0.7861360643,3.3477661635\N,-1.2147906701,0.078778769,2.1149487153\

C,-2.4421403175,1.5070190708,3.6834055637\C,-2.529445066,2.1774498083,4.9065322809\

C,-1.4498223641,2.1938798999,5.7985172255\C,-0.285802707,1.5029225056,5.4432444607\

C,-2.3828466328,-0.5875987229,1.6422718498\C,-3.6463407249,0.0022067894,1.788718934\

S,-3.7880057225,1.6207825669,2.5189662886\C,-4.7934443236,-0.6691850623,1.3485115793\

C,-4.7109027441,-1.9234615477,0.7359552591\C,-3.441775439,-2.514362633,0.6157104395\

C,-2.2984667738,-1.8688890225,1.076089681\C,-1.5265541307,2.9685662182,7.0937126251\

C,-5.9465320738,-2.6266139839,0.2254196734\O,3.5335876273,-0.0728236441,-0.704456983\

H,-3.4475780083,2.7015388902,5.1561490988\H,0.5733736415,1.5103427975,6.1079910824\

H,-1.3299332165,-2.3511799739,0.9973470646\H,-3.3486423999,-3.5033759728,0.1749061637\

H,-5.7629899412,-0.1995699641,1.4903319451\H,3.3742497757,-0.0222189429,1.8925136591\

O,1.2241900555,0.0105769674,-2.0856938307\H,-0.9873551632,0.0887073576,-0.5188541221\

H,-5.9581693028,-3.6793644019,0.5271409337\H,-6.8588882613,-2.1568756078,0.6032454551\

H,-5.9901728764,-2.6024421464,-0.8703942356\H,-0.9054448775,2.5108477638,7.8693511772\

H,-1.1752932041,3.9988337388,6.9566605184\H,-2.5536076042,3.0190278837,7.4673022437\

C,0.0198066745,0.0519472467,-2.8452888384\H,0.3311345925,0.0453147909,-3.8898911753\

H,-0.5477280055,0.9670047059,-2.6366711582\H,-0.6066733533,-0.8250231674,-2.6419562046\\

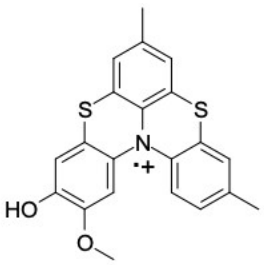

8•+

b3lyp/6-31+g(d,p)

C,-0.0686081765,0.0102834489,0.0040037901\C,-0.0313562811,0.0544061007,1.4179823022\
C,1.221669259,0.0434683131,2.0808393832\C,2.405453474,-0.0651415181,1.3434514751\
C,2.3628504007,-0.1393624519,-0.0419018689\C,1.1060895486,-0.0823389157,-0.7187877038\
S,1.3351300604,0.074574863,3.8337494821\C,-0.172833508,0.9014544944,4.2245912551\
C,-1.2923130695,0.8258527007,3.369066066\N,-1.2305564462,0.1258512779,2.1464368982\
C,-2.4843475839,1.4784689887,3.7620130251\C,-2.546567532,2.1502939819,4.9800986515\
C,-1.437198361,2.2148263912,5.8389860427\C,-0.2533540162,1.5886457611,5.439480838\
C,-2.3949873392,-0.5381166826,1.6610548886\C,-3.6728365362,0.0210241146,1.8397448888\
S,-3.8781323521,1.5661841064,2.6724334273\C,-4.8016503653,-0.6420623671,1.3406293211\
C,-4.6929905715,-1.8693039793,0.6829530246\C,-3.4103796669,-2.4435911789,0.5596546387\
C,-2.2830808924,-1.7977417116,1.0411497462\C,-1.5324981222,2.9407422037,7.1564522656\
C,-5.9088594395,-2.5839407815,0.1523414771\O,3.504937436,-0.2316057207,-0.7374677509\
H,-3.474551057,2.6371062141,5.2653832057\H,0.6250011886,1.6401155767,6.0754881869\
H,-1.3103751799,-2.2693720142,0.958449769\H,-3.302862731,-3.4208602955,0.0981752781\
H,-5.7805034805,-0.1962148406,1.4903553063\H,3.3712439223,-0.0736569122,1.8369116654\
O,1.2211496231,-0.106703716,-2.0662890496\H,-1.0226204151,0.0740517871,-0.5013113339\
H,3.3095056617,-0.2411498625,-1.6899912851\H,-6.010471553,-3.57428914,0.6097413435\
H,-6.8251527389,-2.0234524258,0.3502304307\H,-5.8308681342,-2.7354454931,-0.9301592649\
H,-0.5590769942,3.0123299762,7.6465517485\H,-1.9227266787,3.9545364741,7.0204235727\
H,-2.2145456172,2.4182530502,7.8371083194\C,0.0416261033,-0.0248247564,-2.8808599981\
H,0.3939746959,-0.0632885511,-3.9103001465\H,-0.4817061804,0.9200501602,-2.7024262621\
H,-0.6205373424,-0.8739078299,-2.6829415692\\

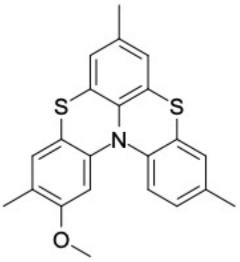

3

b3lyp/6-31+g(d,p)

C,0.0715652247,-0.0004837859,0.0381247601\C,0.072927118,0.080953394,1.4362259282\

C,1.3026373092,-0.1056791529,-0.6210456929\C,2.5007755756,-0.1533220224,0.0974671176\

C,2.5015480154,-0.0364769093,1.4948364341\C,1.2712932932,0.0951826049,2.1561385872\

H,-0.8670871909,0.1485462644,1.9768120436\H,1.3343543431,-0.1686219931,-1.7052598399\

C,-1.2229789387,0.0528370078,-0.7406815819\H,-2.0520552874,-0.3692635121,-0.1645377702\

H,-1.1464541359,-0.5030813848,-1.6801477479\H,-1.4908418701,1.08687332,-0.9918990579\

S,1.2572522148,0.326592945,3.9251167005\S,4.0469385094,-0.4139805176,-0.7564776369\

N,3.7199189212,-0.0538608765,2.2224687381\C,5.1095624305,0.5545636101,0.3021462513\

C,4.8588802351,0.5970160825,1.6823827316\C,6.2057898259,1.2343195857,-0.2410278596\

C,5.7295492004,1.3366875461,2.4970918038\C,6.8401912861,1.9760262004,1.9516406269\

C,7.101458059,1.9363792311,0.5726799764\H,6.3617129583,1.2040126232,-1.3162210527\

H,5.5296117427,1.4022459521,3.5615584\H,7.5023699216,2.5353015904,2.6078592212\

C,8.3126433957,2.6256975568,-0.0120326426\H,8.2110142326,2.7631228176,-1.09250831\

H,9.2252951847,2.0417340845,0.1617721819\H,8.4672032778,3.6112012733,0.4400448309\

C,2.6723399743,-0.6824465083,4.3368669864\C,3.7696106589,-0.7264841187,3.4714841048\

C,2.7084474241,-1.3985304285,5.5407911144\C,3.8277103653,-2.1292868265,5.93374195\

C,4.8933494732,-1.4921736478,3.8257425863\C,4.928353478,-2.1638183094,5.0483751694\

C,3.8753926981,-2.8752729033,7.2424884343\H,2.9433277595,-2.7406182139,7.7980111576\

H,4.7045223397,-2.5279199342,7.8692996248\H,4.0312911802,-3.9483534214,7.0843639529\

O,5.99518643,-2.9110620109,5.4705080839\C,7.1375543971,-3.0116204316,4.6290059817\

H,1.8406175157,-1.3710119933,6.1942206337\H,5.7229444219,-1.5456171926,3.1329440686\

H,7.8476636397,-3.6410482479,5.1668660758\H,7.5877112717,-2.0279613395,4.4462210559\

H,6.887169073,-3.4824051411,3.6701700306\\

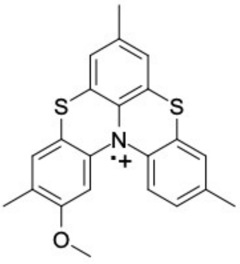

3•+

b3lyp/6-31+g(d,p)

C,0.0034749095,0.0819725098,0.0010646843\C,-0.0035412067,0.0197642091,1.3958144734\
C,1.2434136195,0.0489681589,-0.6602051856\C,2.4407728774,-0.0386877028,0.0430750092\
C,2.4357432856,-0.0797513415,1.4588126232\C,1.188782714,-0.0612507053,2.1235367737\
H,-0.948055865,0.0247136209,1.9311226807\H,1.272805801,0.0876689067,-1.7451990926\
C,-1.2779882543,0.1765805031,-0.7863431661\H,-2.1530077843,0.1602687482,-0.1330615492\
H,-1.3651859416,-0.6547613011,-1.4941300022\H,-1.3077278824,1.1037569979,-1.3694906548\
S,1.0865416431,-0.0623966327,3.8804368256\S,3.9475769819,-0.2298808157,-0.867638517\
N,3.6456859806,-0.1360032631,2.1756180872\C,5.0549808281,0.5435451383,0.2714605884\
C,4.8019163725,0.5155547941,1.6543381357\C,6.2053244182,1.1769789275,-0.2168751875\
C,5.6814213883,1.1933215236,2.520711753\C,6.8101963332,1.8220024657,2.0208828096\
C,7.1064431939,1.8102286619,0.6415523539\H,6.3823596705,1.1894466833,-1.2882406214\
H,5.4629749129,1.2342059675,3.5816682748\H,7.4672473391,2.3502924198,2.7055351589\
C,8.3413780989,2.494420594,0.1151844965\H,8.4058633945,2.4301828102,-0.9732238533\
H,9.2465944279,2.0419681806,0.5357486618\H,8.3508108624,3.5535907463,0.3949699036\
C,2.6044375272,-0.8410115547,4.2820613802\C,3.7193565397,-0.8041803987,3.4138891738\
C,2.7229817685,-1.4711066465,5.5332567159\C,3.8939088472,-2.093369324,5.936699733\
C,4.9101024694,-1.4558288398,3.7942366696\C,4.9965453086,-2.0934613989,5.0265309416\
C,4.015158297,-2.7828201868,7.2648112566\H,3.0913229522,-2.6924441465,7.8394345651\
H,4.8373630047,-2.3577959798,7.850500749\H,4.2454554311,-3.8455329627,7.1330143722\
O,6.0840384702,-2.7629270951,5.4575418521\C,7.2442093713,-2.851696925,4.6223507457\
H,1.8626427001,-1.4868503273,6.1956414128\H,5.7424240958,-1.469658726,3.1047128204\
H,7.9678454498,-3.4336301251,5.1913042375\H,7.6529616142,-1.8566474897,4.4151932385\
H,7.0107109863,-3.3688458116,3.6853228242\\

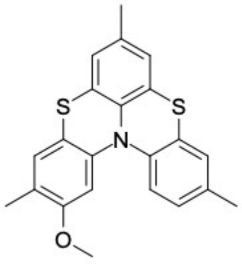

3

b3lyp/6-31+g(d,p) scrf=(solvent=acetonitrile)

C,-0.0051112383,0.0014765324,0.0011801218\C,-0.0048718696,0.0034878991,1.4025875694\

C,1.2296374953,-0.0005987768,-0.6616128275\C,2.4302045772,-0.0226383865,0.0548916238\

C,2.4294902844,0.0146407785,1.4574678922\C,1.1937115798,0.0402839645,2.1221015404\

H,-0.9467447982,-0.0073933859,1.9437238874\H,1.2606541056,-0.004623595,-1.7475177185\

C,-1.3017972723,0.0286961844,-0.7749036728\H,-2.1074572412,-0.4604024162,-0.2196646042\

H,-1.1973175698,-0.4729288885,-1.7414067872\H,-1.6188081929,1.0600985885,-0.9729722949\

S,1.1739764801,0.1645507604,3.9038036112\S,3.9876527551,-0.1513116802,-0.8114001171\

N,3.6483505546,0.0215523861,2.1844190703\C,5.0028446015,0.8101428308,0.3024009632\

C,4.7518577074,0.7616107778,1.6819499041\C,6.0611136478,1.5752716817,-0.2027867615\

C,5.5834513643,1.5001124922,2.5381702441\C,6.6575425448,2.2276298615,2.0303152406\

C,6.9192072471,2.2784327342,0.6507524779\H,6.2208602915,1.610125707,-1.2771333022\

H,5.3874138561,1.4935567269,3.6053719492\H,7.2916364579,2.7810156116,2.7179678782\

C,8.0917287304,3.0619162827,0.1079224851\H,7.9985892171,3.2233916903,-0.9695815979\

H,9.0356853418,2.5314982139,0.2837938563\H,8.1751980163,4.0395818755,0.5939090702\

C,2.6424880982,-0.7927872504,4.2556827092\C,3.7388988991,-0.7235457879,3.3900188833\

C,2.7180042803,-1.5778804337,5.4143154036\C,3.8766563724,-2.2701781471,5.7628226399\

C,4.9037803963,-1.4462697878,3.6987054638\C,4.9777977825,-2.1893371067,4.8787659784\

C,3.9625683616,-3.0890058372,7.0257850303\H,3.0244722072,-3.0355013135,7.5841748676\

H,4.77074513,-2.7358661261,7.6764544092\H,4.172319967,-4.1421015831,6.8064233582\

O,6 .085115777,-2.898436314,5.2536559077\C,7.2400846923,-2.8719695638,4.4113767218\

H,1.8533181707,-1.6350346033,6.0698211266\H,5.7367531549,-1.4112607204,3.008959906\

H,7.9850583553,-3.490034227,4.9124873376\H,7.6250998176,-1.8522883541,4.2999513763\

H,7.0193175813,-3.2936408434,3.4244244803\\

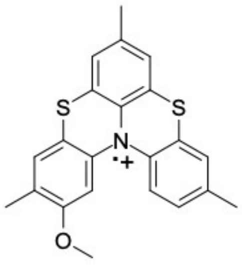

3•+

b3lyp/6-31+g(d,p) scrf=(solvent=acetonitrile)

C,-0.0762638572,0.0825438527,-0.0336838666\C,-0.0786380706,-0.0666826105,1.3536574078\
C,1.1616010902,0.1546963515,-0.6957620047\C,2.3624662523,0.0866255805,0.0027512979\
C,2.363107689,-0.0414074821,1.4131781753\C,1.1184509473,-0.1303542533,2.076238064\
H,-1.0207265503,-0.1437475279,1.8870723684\H,1.1857407292,0.2584980226,-1.7762287848\
C,-1.3628654757,0.1640130674,-0.8144399436\H,-2.2306063849,0.0092079343,-0.1695772355\
H,-1.3851088993,-0.5868522207,-1.6111348808\H,-1.4628480659,1.14563517,-1.2913259568\
S,1.0166580286,-0.2529336152,3.8277136661\S,3.8761935164,0.0315407874,-0.9164852733\
N,3.5748299984,-0.0766194678,2.1250872868\C,4.9448863693,0.7899656727,0.2697556113\
C,4.6930937053,0.6665391918,1.6468822973\C,6.0604312541,1.5098827008,-0.1764571682\
C,5.5337794241,1.3364328948,2.556075018\C,6.6299531678,2.0502034993,2.0991701636\
C,6.9271183257,2.1341181728,0.7234127307\H,6.2390803186,1.5934855873,-1.2441144723\
H,5.313940019,1.3010465854,3.616522423\H,7.2605533138,2.5651451868,2.8175683872\
C,8.1322999866,2.9011712634,0.2433773419\H,8.1676902072,2.951514472,-0.8472933686\
H,9.0564152083,2.4264295422,0.5928973447\H,8.1271157677,3.9234651224,0.6364650838\
C,2.5794683517,-0.9576290215,4.1866482236\C,3.6877980029,-0.8086492225,3.3219852096\
C,2.7388442601,-1.6468931771,5.4009803025\C,3.9470363168,-2.2189453785,5.7673897184\
C,4.915222358,-1.4095067954,3.6639630414\C,5.0438127962,-2.1040527138,4.8599448672\
C,4.1098846113,-2.9690857229,7.0583845585\H,3.1801658081,-2.9637457934,7.6306413971\
H,4.9021770167,-2.5240898645,7.6701756519\H,4.402384313,-4.0082697173,6.8723000595\
O,6.1767746069,-2.7277283651,5.2516658087\C,7.3363908245,-2.6765884044,4.407110122\
H,1.8846277762,-1.7480762714,6.0631105497\H,5.7434184692,-1.3371618241,2.9737609349\
H,8.1055189151,-3.2376701791,4.9358189309\H,7.6660907953,-1.6430549332,4.2618406993\
H,7.1332144805,-3.1483776451,3.4405335133\\

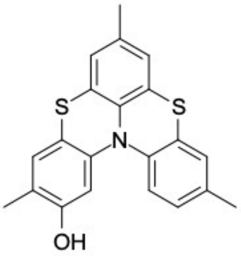

6

b3lyp/6-31+g(d,p)

C,0.1342014859,-0.0511762449,0.0717109137\C,0.1353781772,0.2371428368,1.4422413938\

C,1.3617753292,-0.2999799287,-0.5544351473\C,2.5557079464,-0.28496663,0.1725846347\

C,2.5575424387,0.0396392679,1.536587411\C,1.331702614,0.3131924828,2.1615002967\

H,-0.8026352845,0.4189927435,1.9594201675\H,1.3934457724,-0.5255011759,-1.6167775366\

C,-1.155662306,-0.0658693549,-0.7164057749\H,-1.9986888733,-0.3827596511,-0.0947651805\

H,-1.0924156963,-0.7449173356,-1.5721199404\H,-1.3921716822,0.9323546155,-1.1058274224\

S,1.3224257552,0.8029915718,3.8769721938\S,4.0913666008,-0.7312267736,-0.6211808591\

N,3.7730182284,0.085524213,2.2686100078\C,5.1912591312,0.3400593026,0.2897785164\

C,4.9396353461,0.6004066048,1.6454929449\C,6.3164692924,0.884728506,-0.33981396\

C,5.837671871,1.4189998378,2.3470834981\C,6.9754457196,1.9227314139,1.7215295847\

C,7.2384758364,1.664330062,0.3667037719\H,6.4736480942,0.686450071,-1.3967927999\

H,5.63 76392274,1.6533755901,3.387495856\H,7.6585641834,2.5472226453,2.291753 4112\

C,8.478772204,2.2077392435,-0.3043026789\H,8.3878624873,2.1801342238,-1.3940463701\

H,9.3665283668,1.6229704782,-0.0322896911\H,8.6701209591,3.2446050388,-0.008066651\

C,2.6954773753,-0.1883131703,4.4449273962\C,3.7946565651,-0.3973892547,3.6015262052\

C,2.7014211946,-0.7173453016,5.7406876306\C,3.7942610074,-1.4204742165,6.2513558132\

C,4.8846994521,-1.1381839104,4.0814623222\C,4.8890651899,-1.618866343,5.3898036724\

C,3.8152014919,-1.9656811053,7.6563100075\H,2.8873975363,-1.72142207 57,8.1805587252\

H,4.6539023853,-1.5554447599,8.2301894468\H,3.93632548 07,-3.0548048894,7.659157958\

O,5.9515910867,-2.3287642403,5.8948570544\H,6.6411964998,-2.4121200381,5.2234521984\

H,1.8325392669,-0.5622670374,6.3745806422\H,5.7278868946,-1.3326129726,3.4244087147\\

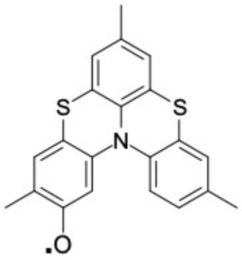

6(-H)•

b3lyp/6-31+g(d,p)

C,0.1141269492,0.0267937231,0.0309887902\C,0.1141882703,0.2261254248,1.41 3636383\

C,1.343252527,-0.2101277851,-0.6023094511\C,2.532605577,-0.2656754754,0.1251090084\

C,2.5375046084,-0.0257294146,1.5110513161\C,1.3111030187,0.2209454391,2.1406488218\

H,-0.8226903742,0.3915431102,1.9380361575\H,1.3761072974,-0.3730347491,-1.6761196859\

C,-1.1684672751,0.0652119513,-0.7672059907\H,-2.0407045166,0.1551483741,-0.1140270704\

H,-1.2904641305,-0.8425535836,-1.3683226223\H,-1.1792266611,0.9168407503,-1.4576129049\

S,1.2514515386,0.5359487926,3.8902304758\S,4.054240765,-0.7169529772,-0.6869368843\

N,3.7506389775,-0.0389962273,2.2397481167\C,5.169754804,0.2968486231,0.267660827\

C,4.9213119471,0.5043285317,1.6306775938\C,6.293144269,0.8658110012,-0.344354366\

C,5.8109968687,1.3082003084,2.358382751\C,6.9430667323,1.8411036689,1.7494955336\

C,7.2104354861,1.6252712605,0.387641544\H,6.4495696942,0.7046624085,-1.4075375245\

H,5.6189177405,1.5005529172,3.4083655495\H,7.6230641954,2.4483328072,2.3411242886\

C,8.4507399016,2.1930963282,-0.2615326376\H,8.3770698896,2.1703465346,-1.3525388548\

H,9.342044216,1.6200417512,0.0223475312\H,8.6209722165,3.2307769876,0.0445279624\

C,2.6669443311,-0.3529173721,4.42 66535446\C,3.7884602238,-0.5519777367,3.5593441361\

C,2.7054888681,-0.8 049364613,5.7643760901\C,3.8082678903,-1.4486804581,6.2867207346\

C,4.8 906203761,-1.2188884353,4.0467611183\C,4.9694328807,-1.6836410566,5.41 58578101\

C,3.8681079155,-1.9366293048,7.7023741273\H,2.9442524256,-1.7 11149016,8.2418315866\

H,4.7115594742,-1.4802596847,8.2324464399\H,4.04 4659787,-3.0175733844,7.7307628004\

O,5.9855162298,-2.2800377414,5.8463 845442\H,1.8300833193,-0.6421551397,6.388478946\

H,5.7551561593,-1.4014 671622,3.4188030738\\

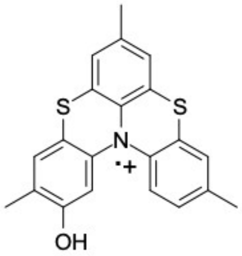

6•+

b3lyp/6-31+g(d,p)

C,0.0733400091,0.0050873281,0.0249109123\C,0.0626552567,0.1549328496, 1.4134584555\

C,1.3125566385,-0.1602340436,-0.6170302973\C,2.5061210255 ,-0.1722153479,0.098079164\

C,2.4978493453,-0.0008776943,1.5045169364\C ,1.2502631129,0.152620345,2.1523388976\

H,-0.8822946571,0.2670472484,1. 9360874556\H,1.3444651527,-0.2861057805,-1.6953076925\

C,-1.2038024743, 0.0172485274,-0.7746322273\H,-2.0816587249,0.0852497466,-0.1285723667\

H,-1.2954278648,-0.8896129889,-1.3816598983\H,-1.2224046547,0.87093822 28,-1.4615700909\

S,1.1449960568,0.4304737567,3.8871491512\S,4.00660117 45,-0.5365130989,-0.7669852162\

N,3.703342109,0.0172064329,2.2292036494 \C,5.1384116532,0.3542892564,0.2544527039\

C,4.8828913081,0.5403168813, 1.6253683431\C,6.3145558905,0.8618356983,-0.3132763395\

C,5.7892348657, 1.302471303,2.3889007491\C,6.9424212573,1.805133133,1.8096880079\

C,7.2 394017689,1.5783847483,0.4487867633\H,6.4937513951,0.7084894718,-1.373 3119371\

H,5.5715214483,1.5090119387,3.4304873161\H,7.6197596582,2.4017 649641,2.4135885727\

C,8.5011468009,2.1284334836,-0.1634664763\H,8.5702 068442,1.8924905593,-1.2275904787\

H,9.3866656682,1.7167815913,0.3338281558\H,8.5465252914,3.2174357875,-0.0522927906\

C,2.635875365,-0.3285828414,4.4141765266\C,3.7514271607,-0.4585031283,3.5557693604\

C,2.730482151,-0.7697673923,5.7446258128\C,3.8805216075,-1.3639500305,6.2463085066\

C,4.9151411169,-1.086285457,4.0401541063\C,4.9762237701,-1.53201207 52,5.3528018517\

C,3.9814507465,-1.8535641849,7.6620868177\H,3.06132004 03,-1.6527971531,8.2141117771\

H,4.8161747696,-1.3710325088,8.181968545 9\H,4.1800030781,-2.9304971392,7.688687775\

O,6.0609847637,-2.163369448 7,5.8638259156\H,6.7640919338,-2.26000051,5.206288155\

H,1.8686458408,-0.6589437452,6.3958776437\H,5.7572669526,-1.2392743663,3.3740861605\\

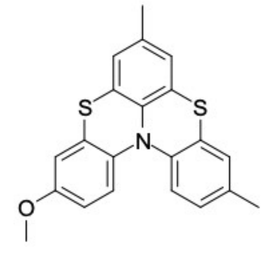

2

b3lyp/6-31+g(d,p) scrf=(solvent=acetonitrile)

C,0.067621836,-0.0072615474,0.0304137591\C,0.0638354756,-0.0786734329,1.4300455472\

C,1.2612653956,-0.1298387034,2.1503500893\C,2.4980022864,-0.0494874486,1.4930304404\

C,2.5018123242,0.0624709025,0.0943313163\C,1.304064238,0.0530410873,-0.6269240574\

C,-1.2259557965,-0.02061587,-0.7511309122\H,-0.8795275546,-0.1117410956,1.9677218733\

H,1.337386661,0.1151206994,-1.7110502458\S,4.0569935946,0.2822160156,-0.7587626768\

N,3.7117475297,-0.0681655236,2.2239400236\C,4.8395022615,-0.7439369163,1.6813798677\

C,5.0998064151,-0.7040119568,0.3062646353\C,6.1815030369,-1.4022774653,-0.2507760082\

C,7.0423299485,-2.1214167666,0.5844041645\C,5.6887783359,-1.5103539709,2.4979557561\

C,6.7871240457,-2.1724983926,1.9650878293\O,8.138677768,-2.8125466157,0.1501326626\

H,7.447610143,-2.7498836091,2.6035814947\H,6.3359135819,-1.3645887746,-1.3220222639\

S,1.2380265297,-0.3344074817,3.9250225735\C,3.7875010943,0.624850776,3.4604373826\

C,2.687727867,0.630258028,4.3327313425\C,2.733148658,1.3495639666,5.5326956278\

C,4.9298333643,1.3553315623,3.8228280097\C,3.885285215,2.0459025574,5.9174580556\

C,4.9805195669,2.0357743475,5.0378430085\H,5.7805005099,1.3816131834,3.1497604988\

H,5.8822692838,2.5843462098,5.2969476179\H,1.8595076688,1.3524041282,6.1790358317\

C,3.9524129807,2.7757161493,7.2390079197\H,2.9540512806,2.9474503114,7.6510568082\

H,4.4493987035,3.7454522895,7.1323556192\H,4.5214812167,2.1997302315,7.9792458403\

H,-2.0433348897,0.4214577391,-0.1740562738\H,-1.5205112464,-1.0464086045,-1.0050755398\

H,-1.1293163734,0.5339085302,-1.6891086156\H,5.4887992177,-1.5732017974,3.5624322539\

C,8.4448084063,-2.8002994089,-1.2468082949\H,9.3465672082,-3.4023105765,-1.3572399974\

H,8.6394330102,-1.7812808227,-1.5996112981\H,7.6334044268,-3.2460587307,-1.8330858724

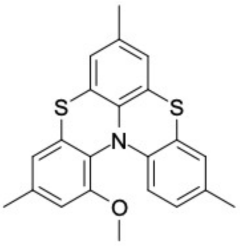

4

b3lyp/6-31+g(d,p)

C,0.1760152683,-0.1313410654,0.1821175307\C,0.2333254756,-0.4161836186,1.5520403811\
C,1.4507752065,-0.3975950476,2.2397707459\C,2.6355012679,-0.0433172422,1.5800616699\
C,2.5704785762,0.3061738413,0.2239871375\C,1.3616363861,0.2279628238,-0.472850477\
C,-1.1286898798,-0.2233599869,-0.5759091534\H,-0.6754264041,-0.6629684348,2.0938675519\
H,1.3440091091,0.4726375026,-1.5313900128\S,4.0336352709,0.9358492796,-0.582234847\
N,3.8656560317,-0.0062675331,2.2835382779\C,5.0573264095,-0.3776761451,1.6113554084\
C,5.265508988,-0.0344333081,0.2713950543\C,6.4217726416,-0.4297186308,-0.4148474743\
C,7.4148313099,-1.1587398439,0.2381156195\C,6.0303014118,-1.1911639459,2.2477242382\
C,7.2031648359,-1.5396405659,1.573611161\O,5.7366930934,-1.5830123421,3.5177171885\
C,8.6931930482,-1.5460899793,-0.4691165498\H,7.9542751132,-2.1408859731,2.0731230612\
H,6.537362324,-0.1603826512,-1.4603878046\S,1.528169218,-0.8191466546,3.9719112533\
C,3.881467468,0.5693454737,3.5802254906\C,2.8206888164,0.313736763,4.4624926078\
C,2.7960957353,0.9031531234,5.7299964516\C,4.9105250493,1.4219679731,3.997334695\
C,3.8419533497,1.7247758297,6.1682233773\C,4.8968788835,1.9731873459,5.2773985718\
H,5.7239048683,1.6444308925,3.3137489565\H,5.7124991495,2.625143855,5.5803749865\
H,1.9505866372,0.7078651299,6.3846026504\C,3.8374994521,2.3147877384,7.5600282536\
H,8.6216576768,-1.3653703425,-1.5452587063\H,8.9274296523,-2.6060227141,-0.3199782292\
H,9.5457736525,-0.9680941119,-0.0915449916\H,2.8212034625,2.39 7041668,7.9584052892\
H,4.2796074247,3.3164442115,7.5695065336\H,4.4170 954344,1.6962498783,8.2573795692\
H,-1.9848742031,-0.0464298474,0.08207 63291\H,-1.2572678895,-1.2174845192,-1.022361499\
H,-1.1702832356,0.508 0047516,-1.3891285353\C,6.6686246777,-2.3922849822,4.2240430849\
H,6.21 53197321,-2.5786196426,5.1981223247\H,7.6257806756,-1.873720314,4.3600
161549\H,6.8372766263,-3.3471333272,3.7108478034\\

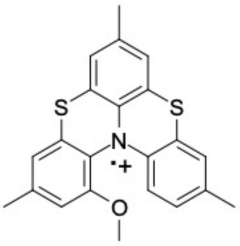

4•+

b3lyp/6-31+g(d,p)

C,0.129861814,-0.1668151399,0.1323726087\C,0.1700926176,-0.3330787907,1.5203122593\
C,1.3726064245,-0.2431507585,2.2271231163\C,2.5825006041,0.0074485463,1.5410404374\
C,2.53739635,0.2154194012,0.1417269914\C,1.3288709419,0.1193922641,-0.5419111352\
C,-1.1639258597,-0.288439129,-0.6305736074\H,-0.748794131,-0.523224545,2.066261377\
H,1.3161216585,0.2764933859,-1.6163838763\S,3.9768076412,0.7534953004,-0.7338141405\
N,3.8057715104,0.0675047368,2.2302881358\C,5.0008279653,-0.33934255,1.5866359891\
C,5.2169686787,-0.0635819905,0.2217779891\C,6.4160000688,-0.4172734149,-0.401840931\
C,7.4128014123,-1.0885128529,0.3097320974\C,5.9771037543,-1.1144500051,2.2859561316\
C,7.1681618852,-1.450497464,1.6470388754\O,5.6507087164,-1.5009798648,3.5377674346\
C,8.7200633872,-1.4563638253,-0.3434914124\H,7.9153084848,-2.0287636444,2.1769314266\
H,6.5575218358,-0.188569409,-1.4530322102\S,1.3482409723,-0.5122765385,3.9688975415\
C,3.850716075,0.5938511197,3.5387323328\C,2.7749019162,0.4083147454,4.4330303027\
C,2.8544077584,0.9153513246,5.7373263061\C,4.9694223569,1.3376508923,3.9643290994\
C,3.9711675428,1.6306887221,6.1696246216\C,5.0230094526,1.8441846149,5.2486775677\
H,5.7784612545,1.5251551324,3.2670970746\H,5.8844156625,2.432939144,5.5501420401\
H,2.0178931135,0.7624858074,6.412793576\C,4.0487841408,2.1965082659,7.5630245244\
H,8.7022251799,-1.2590946257,-1.4174800177\H,8.9532217508,-2.5154936283,-0.1929538316\
H,9.5437583677,-0.8776521368,0.0910520532\H,3.1779510067,1.9199049858,8.1613432475\
H,4.1084715099,3.2905649911,7.5357890641\H,4.9461364912,1.8388303549,8.0801634914\
H,-2.02531104,-0.3109742759,0.0406403583\H,-1.1757880504,-1.211210426,-1.2226626436\
H,-1.2924361383,0.5460750937,-1.3269370209\C,6.5510058782,-2.3422278078,4.2732071789\
H,6.0532400445,-2.5345722536,5.222627183\H,7.5034044633,-1.8327405363,4.45191
88552\H,6.7188823288,-3.2866354053,3.7458506679\\

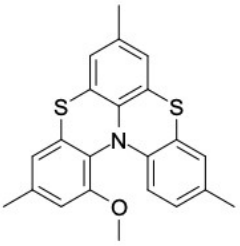

4

b3lyp/6-31+g(d,p) scrf=(solvent=acetonitrile)

C,-0.0059002946,0.0046868939,0.0054361243\C,-0.0079627508,-0.0026362146,1.4068471246\
C,1.1918961506,-0.0098894119,2.1255321675\C,2.4249904617,0.0361545025,1.4602923987\
C,2.4270893125,0.1132257343,0.0599322126\C,1.2284526275,0.0629020231,-0.6578108944\
C,-1.2997394638,-0.0608508437,-0.7731293654\H,-0.950236321,-0.0119430653,1.9471727675\
H,1.2575289005,0.095224726,-1.7433966106\S,3.975996254,0.3709521646,-0.7924877204\
N,3.6370622838,0.0447054943,2.1963442626\C,4.7804721069,-0.6027950781,1.6575790764\
C,5.0560717476,-0.5589344522,0.2867836 681\C,6.1603430377,-1.224383176,-0.2623634342\
C,7.0354580275,-1.933201 1295,0.562319952\C,5.6237984755,-1.3943674116,2.4804729359\
C,6.7505845 745,-2.016132057,1.935632516\O,5.2598452384,-1.492804058,3.7885719311\
C,8.2631135427,-2.611163799,-0.000860858\H,7.4059306101,-2.5987767717,2.572304\
H,6.3323402149,-1.1802434411,-1.3335351383\S,1.1924151641,-0.0663715391,3.9115166927\
C,3.7185572703,0.885194258,3.3375943929\C,2.6207886697,0.9710923347,4.2083328726\
C,2.6580598046,1.8202499457,5.319217396\C,4.8508922794,1.664128007,3.6079827421\
C,3.8018474056,2.5736007206,5.615842483\C,4.8941395667,2.4794194659,4.7386644245\
H,5.7004659498,1.6206436085,2.9336787874\H,5.7886909057,3.0658469557,4.9317458377\
H,1.787261423,1.8828078344,5.9665098124\C,3.8610331514,3.4467458128,6.8483198011\
H,8.2379098242,-2.6335965617,-1.0936133072\H,8.3497456653,-3.6414447288,0.3604275828\
H,9.176210756,-2.084325843,0.3015740349\H,2.8600062523,3.7379918811,7.1796644682\
H,4.4377950828,4.3581542454,6.6619523446\H,4.343431357,2.9214625187,7.682242381\
H,-2.1312971084,0.3560515124,-0.1977136791\H,-1.5573886267,-1.0985196515,-1.0188043689\
H,-1.2250966915,0.489151225,-1.7159615725\C,6.0790824333,-2.2578318199,4.6777834787\
H,5.5939796385,-2.1917482598,5.6514573459\H,7.0893730479,-1.839572938,4.7417989418\
H,6.1306205376,-3.3060396111,4.3639860806\\

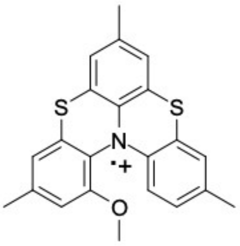

4•+

b3lyp/6-31+g(d,p) scrf=(solvent=acetonitrile)

C,-0.0593468806,-0.0398329681,-0.0507253196\C,-0.0615975469,0.08835969,1.3411200069\
C,1.1332604686,0.1634550373,2.0629122842\C,2.3746324767,0.0961469153,1.3915901172\
C,2.3762080592,0.0067392265,-0.0209350365\C,1.1755680418,-0.0681887914,-0.7202114455\
C,-1.3474786254,-0.1399196431,-0.826592514\H,-1.0043612191,0.1418363628,1.8761224329\
H,1.1973128683,-0.141018972,-1.8032002228\S,3.8877634177,0.1443195513,-0.9292683733\
N,3.5845205363,0.1323664296,2.1038294932\C,4.7190600872,-0.5560129458,1.609476235\
C,4.9927164894,-0.6023976548,0.2285665022\C,6.1452786758,-1.2262779806,-0.2550133624\
C,7.0318582051,-1.8508920988,0.625266923\C,5.5703699034,-1.2854502798,2.4961613106\
C,6.719169683,-1.8932155244,1.9955661492\O,5.1716743535,-1.3608292121,3.7831909645\
C,8.291793514,-2.5067297771,0.1213461526\H,7.3752448795,-2.4344569862,2.6660437834\
H,6.3363776454,-1.2406145871,-1.3228608881\S,1.0462042912,0.2783892275,3.8192708232\
C,3.6883046409,0.9089762764,3.2769475499\C,2.5879371128,1.0619673316,4.1465386715\
C,2.7266209457,1.8071523975,5.3257769049\C,4.8984358803,1.5615846445,3.5866755072\
C,3.9320272013,2.4297399795,5.6491234283\C,5.0126055392,2.3050321878,4.7456902009\
H,5.7333955528,1.4881646283,2.8993240311\H,5.9484353162,2.8130822315,4.957831811\
H,1.8713852444,1.9108200915,5.9864375557\C,4.0800983017,3.2405352374,6.9096191851\
H,8.3337968219,-2.5054292339,-0.9700247452\H,8.3624362969,-3.5421202571,0.4706015037\
H,9.1755895125,-1.9780107537,0.49701428\H,3.1545716742,3.2546086913,7.4894427425\
H,4.3565999365,4.2748895473,6.6764223525\H,4.8758927058,2.8303926216,7.5417237327\
H,-2.2145950556,0.0244309032,-0.1832310149\H,-1.4450714938,-1.1320676248,-1.2821311458\
H,-1.3735536581,0.593467332,-1.639021799\C,5.9743659866,-2.0872064138,4.7278153202\
H,5.4452173736,-2.0047017041,5.6759179846\H,6.9683929523,-1.6391526835,4.8170134291\
H,6.0565403828,-3.1391104471,4.4386725873\\

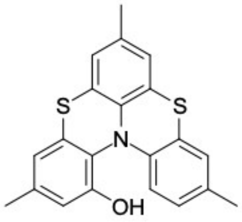

7

b3lyp/6-31+g(d,p)

C,0.0864675583,0.04308418,0.1042635526\C,0.0800943352,-0.1421029426,1.4926845953\

C,1.2791716551,-0.2636193415,2.2006893262\C,2.5099651157,-0.1616324247,1.5414596354\

C,2.5223003948,0.1027719452,0.1650298488\C,1.3212426752,0.1673168274,-0.5475885151\

C,-1.2068672605,0.0986068689,-0.676193834\H,-0.862096253,-0.1959867118,2.0305130261\

H,1.351263573,0.3398474197,-1.6199750416\S,4.0666411758,0.4658317646,-0.6541871738\

N,3.7170845508,-0.2728750076,2.2816366323\C,4.8690703346,-0.8202126046,1.6494632252\

C,5.1599087789,-0.573936728,0.3006486588\C,6.283418698,-1.1370964312,-0.3109075313\

C,7.1560549323,-1.9515135857,0.422564841\C,5.6937948911,-1.7236283956,2.3532844204\

C,6.8409609911,-2.2463797238,1.753223693\O,5.4199682134,-2.1108872637,3.6307641183\

C,8.4082642593,-2.5110366752,-0.2120524043\H,7.4613675305,-2.9176116385,2.3392709934\

H,6.4712903731,-0.9435786215,-1.3628173876\S,1.2940613839,-0.5196234335,3.9693039393\

C,3.8161170988,0.4834227857,3.4848573724\C,2.7187630301,0.490065863,4.3629114976\

C,2.7638424088,1.2353830558,5.5446182227\C,4.957428161,1.2211821444,3.8220511016\

C,3.9132483678,1.9506732175,5.903935899\C,5.0048207348,1.9281929055,5.0225265511\

H,5.8051375316,1.2301855637,3.1446204637\H,5.904392124,2.4859139056,5.2696967123\

H,1.8933342217,1.2457793915,6.1950642003\C,3.9809070515,2.7089186473,7.2095532343\

H,4.5440423703,-1.7891713004,3.8925135131\H,8.3162786217,-2.55879521,-1.301093907 7\

H,8.6258056411,-3.5187033086,0.1557404753\H,9.2786590047,-1.8838688351,0.0181703656\

H,2.9815769594,2.9459448303,7.5864404767\H,4.5306805927,3.6488583838,7.0959444472\

H,4.4938899321,2.1224815852,7.9822454316\H ,-2.0330217834,0.4543402684,-0.0532828187\

H,-1.4828290144,-0.894330153 5,-1.0527364316\H,-1.1232239429,0.7645566492,-1.5406090564\\

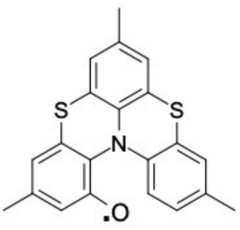

7(-H)•

b3lyp/6-31+g(d,p)

C,0.0738474229,0.0482893297,0.0185817796\C,0.0912342852,-0.0619797272, 1.411912336\

C,1.2979610838,-0.1015524663,2.1213108769\C,2.5197890145,0 .0121604665,1.4413530759\

C,2.5060316232,0.142187325,0.0434972332\C,1.2 991887978,0.1402235043,-0.6568530763\

C,-1.2280983565,0.0532999626,-0.7 482823705\H,-0.8432256053,-0.1231729049,1.9620319458\

H,1.3177523974,0. 2193063685,-1.7402190358\S,4.0454121249,0.3570874258,-0.8230481068\

N,3 .748435248,-0.0286748228,2.1595621156\C,4.8608534793,-0.6606779441,1.6 171041633\

C,5.0794804127,-0.6574076622,0.2238505175\C,6.1464709847,-1. 3424143738,-0.3379942583\

C,7.0782604419,-2.0601941631,0.4727112523\C,5 .7571073333,-1.4624305847,2.48635067\

C,6.8790987725,-2.1077783549,1.82 92688682\O,5.5432051112,-1.5968268071,3.7096830365\

C,8.2416212139,-2.7 574860978,-0.19051894\H,7.5522588269,-2.6545865341,2.4826148756\

H,6.28 55087523,-1.3151439309,-1.4151125881\S,1.26679248,-0.3468483925,3.8819 388145\

C,3.8124642548,0.6227000526,3.4253025286\C,2.7261788222,0.5767228713,4.3076138951\

C,2.7945552132,1.2348097814,5.5411003434\C,4.941082 3063,1.3752070955,3.7773031374\

C,3.9394369942,1.9388536084,5.923149502 2\C,5.0078048439,2.0070372065,5.0117045537\

H,5.7685367889,1.4454934125 ,3.0789715223\H,5.8988612716,2.5729715044,5.2698098524\

H,1.9373996909,1.1934653309,6.2075635588\C,4.0277945794,2.6135916544,7.2713577567\

H,7 .8927878267,-3.486604432,-0.9315204113\H,8.8569651183,-3.2861527225,0. 5417238074\

H,8.8817091953,-2.040957857,-0.7191436391\H,3.0528928684,2. 6506288169,7.7655361878\

H,4.4003545405,3.6396902408,7.1799686875\H,4.7 164457842,2.0755769138,7.934320268\

H,-2.0845480224,0.1616462399,-0.0773084275\H,-1.3591054884,-0.8809767621,-1.3074390955\

H,-1.260242336,0.8 737816829,-1.4730485405\\

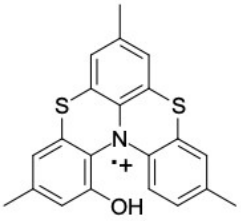

7•+

b3lyp/6-31+g(d,p)

C,0.0331028468,0.0313085748,0.014877479\C,0.0349433004,-0.01844543 79,1.417242886\

C,1.2261554539,-0.0623365374,2.1388650077\C,2.468626933 8,-0.0609378204,1.4607416164\

C,2.4696898757,0.0172775961,0.0481846253\ C,1.2642221257,0.0576373163,-0.6531532919\

C,-1.2636746765,0.0400801823 ,-0.7516096619\H,-0.9080802638,-0.0165809483,1.9554723166\

H,1.28650387 49,0.1121756458,-1.7372592568\S,3.9769133966,0.2150300551,-0.844646572 2\

N,3.6766722686,-0.1156307035,2.1768962166\C,4.8206383321,-0.73053344 37,1.6186610612\

C,5.0841637708,-0.6412213116,0.2276792487\C,6.23543860 71,-1.1981915433,-0.3245808124\

C,7.1465335309,-1.8937919216,0.48096508 97\C,5.7077352022,-1.5131301362,2.417406407\

C,6.852541242,-2.055169720 2,1.8421943759\O,5.4990866011,-1.8211352673,3.7199139693\

C,8.402467193 6,-2.4870233382,-0.1005226776\H,7.5006648952,-2.6431908075,2.4841146644\

H,6.4099396734,-1.1121630864,-1.3921369418\S,1.1467525931,-0.2141188574,3.8983281879\

C,3.7718856953,0.5381779427,3.4355704451\C,2.6717912478,0.566999703,4.3197370227\

C,2.7991222241,1.1896035673,5.5682911476\C,4.9653131329,1.192996343,3.8048121845\

C,3.9907459076,1.8084840823,5.9539000723\C,5.0670157171,1.8101816204,5.0392629436\

H,5.7976125522,1.21 77562522,3.1101151078\H,5.9905513686,2.3178706667,5.3005468098\

H,1.9440787453,1.2037007522,6.2375329141\C,4.1241491929,2.4844861792,7.2926237749\

H,4.7161263496,-1.3920570612,4.091266352\H,8.4068179562,-2.4327852974,-1.191262024\

H,8.5159202662,-3.5351178884,0.1941131565\H,9.285060403,-1.9505035511,0.2675883626\

H,3.2047307057,2.4107458195,7.8772013707\H,4.3666853931,3.5458926604,7.1699214454\

H,4.935785058,2.03417763,7.8750888252\H,-2.0419474092,0.5858954692,-0.2112399157\

H,-1.6255229616,-0.9847272654,-0.9007015957\H,-1.1436813041,0.4950817516,-1.7377559677\\

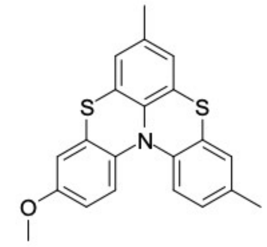

2

b3lyp/6-31+g(d,p)

C,0.0701905421,-0.0096645563,0.0309725248\C,0.0667662504,-0.0772498691,1.4298438312\
C,1.2633946615,-0.12684974,2.1507754428\C,2.4986901742,-0.0459207034,1.4917344397\
C,2.5032898406,0.0638637753,0.093396974\C,1.3053487838,0.049818526,-0.6266174833\
C,-1.2240048351,-0.0272488599,-0.7499599729\H,-0.8757273255,-0.1064081879,1.9695663219\
H,1.3403802275,0.105754452,-1.7111531172\S,4.0558306911,0.2866549365,-0.7603367338\
N,3.7127765407,-0.0623445726,2.2216894563\C,4.8400304668,-0.7371342394,1.678942933\
C,5.098009319,-0.6977462991,0.3038615126\C,6.179756718,-1.3976871925,-0.2513025361\
C,7.0412473175,-2.1150691898,0.5832433446\C,5.6899801962,-1.5029566103,2.4953089573\
C,6.78806293,-2.1646571449,1.9634032434\O,8.1377145903,-2.8085395682,0.1508276837\
H,7.4510966564,-2.7439061616,2.5970961501\H,6.3288360491,-1.3638385259,-1.3236504868\
S,1.2382864908,-0.3307491236,3.9235215031\C,3.7877917273,0.6274257572,3.4588132089\
C,2.6870735003,0.6317489979,4.3300983939\C,2.7347875496,1.3492219548,5.530238093\
C,4.9304517942,1.3553116011,3.8229810621\C,3.8872030874,2.0422089443,5.9172824679\
C,4.9822333333,2.0322775721,5.0392799579\H,5.7790946625,1.3821934479,3.1474655387\
H,5.8843880636,2.5800180467,5.299802241\H,1.8588919196,1.3544023665,6.1737396497\
C,3.9538750492,2.7687116888,7.2408042743\H,2.9541549207,2.9667700077,7.6385349611\
H,4.4740591375,3.7274606854,7.1433552355\H,4.4961009061,2.1792234521,7.9909535846\
H,-2.0409515419,0.4221074499,-0.1771325528\H,-1.5225768927,-1.0537020879,-0.9976394627\
H,-1.1291221923,0.5223366092,-1.6915027571\H,5.4864931184,-1.5648759392,3.5591923454\
C,8.4399519977,-2.8029270148,-1.2386915059\H,9.3420152884,-3.4058037584,-1.3492285264\
H,8.6353381476,-1.785721 9308,-1.601229825\H,7.6287953634,-3.2511657929,-1.8264345794\\

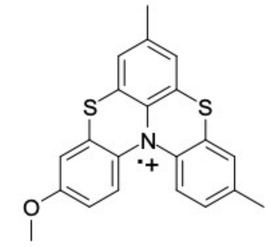

2•+

b3lyp/6-31+g(d,p)

C,-0.0013725787,0.0070582383,-0.006642539\C,0.0029355893,0.0324128163,1.3937111948\
C,1.1959548658,0.0120831961,2.1175269882\C,2.4381656414,-0.0226441186,1.4427071276\
C,2.4344721292,-0.0456435066,0.029220451\C,1.2305840604,-0.0325451255,-0.6739516625\
C,-1.2966053247,-0.0054518558,-0.7768694201\H,-0.9385589123,0.0709821768,1.9331972342\
H,1.2533498634,-0.0546359268,-1.7593740666\S,3.942332107,0.0176526278,-0.8910915813\
N,3.6490242726,-0.038952443,2.1615305804\C,4.7786691693,-0.7087911627,1.6464344761\
C,5.0222520977,-0.7720328604,0.2561442507\C,6.1610360999,-1.415015748,-0.2402507296\
C,7.0609503613,-2.0220118028,0.6450804791\C,5.6777636301,-1.3647820727,2.5215954514\
C,6.7940291938,-2.0066248803,2.035852207\O,8.1779393996,-2.666432442,0.278374186\
H,7.4744416369,-2.5269884216,2.7005048439\H,6.3203473705,-1.4507670874,-1.3108115634\
S,1.1040062186,-0.0771171079,3.8791656456\C,3.7419126232,0.6272115021,3.4113660106\
C,2.6416533212,0.6814562903,4.290278181\C,2.7690160452,1.3193495881,5.5312984713\
C,4.9356738397,1.2834198903,3.7763480355\C,3.9628676658,1.9328258895,5.9148245043\
C,5.0395733942,1.918090548,5.0015735402\H,5.7677993115,1.3044997535,3.082148064\
H,5.963677314,2.4290257704,5.2549880344\H,1.9107743221,1.349599931,6.1960258264\
C,4.0973370797,2.6216209454,7.2473547356\H,3.1679238077,2.5809878544,7.8194602021\
H,4.3722 755795,3.6741018705,7.117136431\H,4.8864576188,2.1544973657,7.84750752 01\
H,-2.1011087316,0.4730940339,-0.2127050766\H,-1.6077882647,-1.0364072519,-0.9856019338\
H,-1.1957074302,0.5080143733,-1.7367032852\H,5.4706086926,-1.3794812162,3.5850130518\
C,8.5335786408,-2.7516782415,-1.110077307\H,9.471812149,-3.303413467,-1.1337779447\
H,8.6805722835,-1.7530 839198,-1.5343301354\H,7.7693790725,-3.2981408003,-1.672334686\\

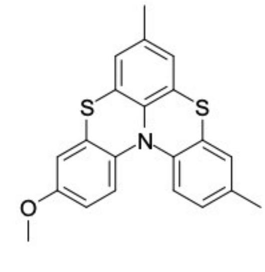

2

b3lyp/6-31+g(d,p) scrf=(solvent=acetonitrile)

C,0.067621836,-0.0072615474,0.0304137591\C,0.0638354756,-0.0786734329,1.4300455472\

C,1.2612653956,-0.1298387034,2.1503500893\C,2.4980022864,-0.0494874486,1.4930304404\

C,2.5018123242,0.0624709025,0.0943313163\C,1.304064238,0.0530410873,-0.6269240574\

C,-1.2259557965,-0.02061587,-0.7511309122\H,-0.8795275546,-0.1117410956,1.9677218733\

H,1.337386661,0.1151206994,-1.7110502458\S,4.0569935946,0.2822160156,-0.7587626768\

N,3.7117475297,-0.0681655236,2.2239400236\C,4.8395022615,-0.7439369163,1.6813798677\

C,5.0998064151,-0.7040119568,0.3062646353\C,6.1815030369,-1.4022774653,-0.2507760082\

C,7.0423299485,-2.1214167666,0.5844041645\C,5.6887783359,-1.5103539709,2.4979557561\

C,6.7871240457,-2.1724983926,1.9650878293\O,8.138677768,-2.8125466157,0.1501326626\

H,7.447610143,-2.7498836091,2.6035814947\H,6.3359135819,-1.3645887746,-1.3220222639\

S,1.2380265297,-0.3344074817,3.9250225735\C,3.7875010943,0.624850776,3.4604373826\

C,2.687727867,0.630258028,4.3327313425\C,2.733148658,1.3495639666,5.5326956278\

C,4.9298333643,1.3553315623,3.8228280097\C,3.885285215,2.0459025574,5.9174580556\

C,4.9805195669,2.0357743475,5.0378430085\H,5.7805005099,1.3816131834,3.1497604988\

H,5.8822692838,2.5843462098,5.2969476179\H,1.8595076688,1.3524041282,6.1790358317\

C,3.9524129807,2.7757161493,7.2390079197\H,2.9540512806,2.9474503114,7.6510568082\

H,4.4493987035,3.7454522895,7.1323556192\H,4.5214812167,2.1997302315,7.9792458403\

H,-2.0433348897,0.4214577391,-0.1740562738\H,-1.5205112464,-1.0464086045,-1.0050755398\

H,-1.1293163734,0.5339085302,-1.6891086156\H,5.4887992177,-1.5732017974,3.5624322539\

C,8.4448084063,-2.8002994089,-1.2468082949\H,9.3465672082,-3.4023105765,-1.3572399974\

H,8.6394330102,-1.7812808227,-1.5996112981\H,7.6334044268,-3.2460587307,-1.8330858724\\

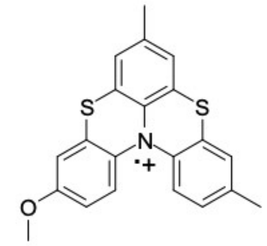

2•+

b3lyp/6-31+g(d,p) scrf=(solvent=acetonitrile)

C,-0.0015206377,0.009216339,-0.0102010093\C,0.0006384187,0.0326010089,1.3915883019\
C,1.192094563,0.0064972871,2.1144922484\C,2.4352722505,-0.0330717322,1.4401879408\
C,2.4324142258,-0.0565352813,0.02768982\C,1.2278931337,-0.0356353624,-0.6774388073\
C,-1.3006159995,0.0073352863,-0.7745138483\H,-0.941264784,0.0709663839,1.929951779\
H,1.2515154966,-0.0565559093,-1.7623963271\S,3.9373364335,-0.0130212966,-0.8955085533\
N,3.6436022059,-0.0508970311,2.1600702179\C,4.7739185294,-0.7185236459,1.6484414663\
C,5.0209634503,-0.7840294407,0.2590379004\C,6.165583041,-1.419334891,-0.2331974479\
C,7.064225424,-2.0177762117,0.656689149\C,5.6727808306,-1.3673825483,2.5278125276\
C,6.7942073735,-2.0037916444,2.0453884648\O,8.1910068746,-2.6556129949,0.2876481519\
H,7.4734537159,-2.5156471223,2.7176126465\H,6.330152582,-1.4524722939,-1.3025536798\
S,1.1001774577,-0.0833406873,3.8773655056\C,3.7373290442,0.6189690537,3.4072222435\
C,2.6398277837,0.6729535023,4.288145318\C,2.7696970387,1.3116861512,5.5280972838\
C,4.9310659595,1.2780383436,3.7652754406\C,3.9653855348,1.9252421823,5.9061462873\
C,5.0387462351,1.9133398278,4.9902091196\H,5.7601026817,1.2999013432,3.0680033633\
H,5.9637213941,2.4238052665,5.2402977149\H,1.9146083157,1.3401655587,6.196327944\
C,4.104544136,2.6094613279,7.2410470273\H,3.1687188484,2.586207455,7.8039534293\
H,4.4054522798,3.6550363108,7.1137444324\H,4.8794299192,2.1230249318,7.8445508563\
H,-2.0244011285,0.6919570699,-0.3226834352\H,-1.7482814522,-0.9936679741,-0.7652652736\
H,-1.1501544997,0.2973674021,-1.8169632483\H,5.463613432,-1.3813793783,3.5904784478\
C,8.5407780523,-2.7217500177,-1.1055376219\H,9.4850678385,-3.2624297735,-1.141101768\
H,8.6719296768,-1.7172452151,-1.5187388453\H,7.7789895499,-3.2683283774,-1.6695293699

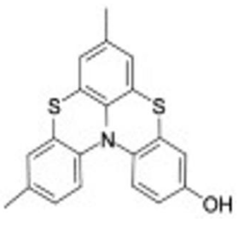
S
S
N
OH

5

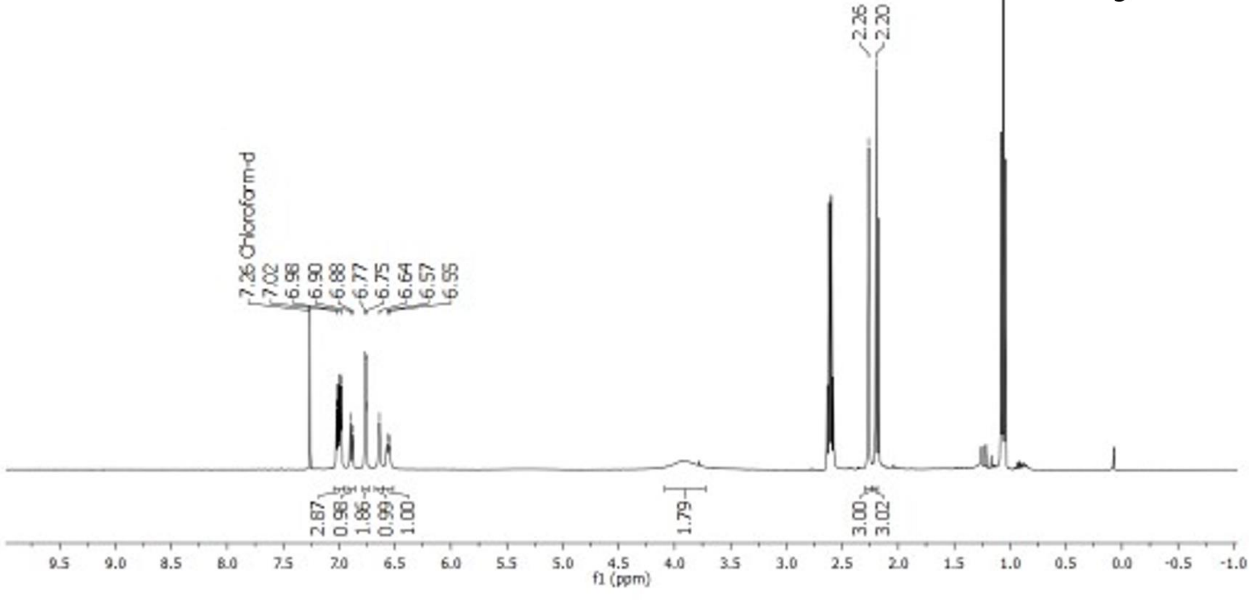
7.26 Chloroform-d
7.02
6.98
6.90
6.88
6.77
6.75
6.64
6.57
6.55
2.26
2.20
2.87
0.98
1.86
0.99
1.00
1.79
3.00
3.02
9.5 9.0 8.5 8.0 7.5 7.0 6.5 6.0 5.5 5.0 4.5 4.0 3.5 3.0 2.5 2.0 1.5 1.0 0.5 0.0 -0.5 -1.0
f1 (ppm)

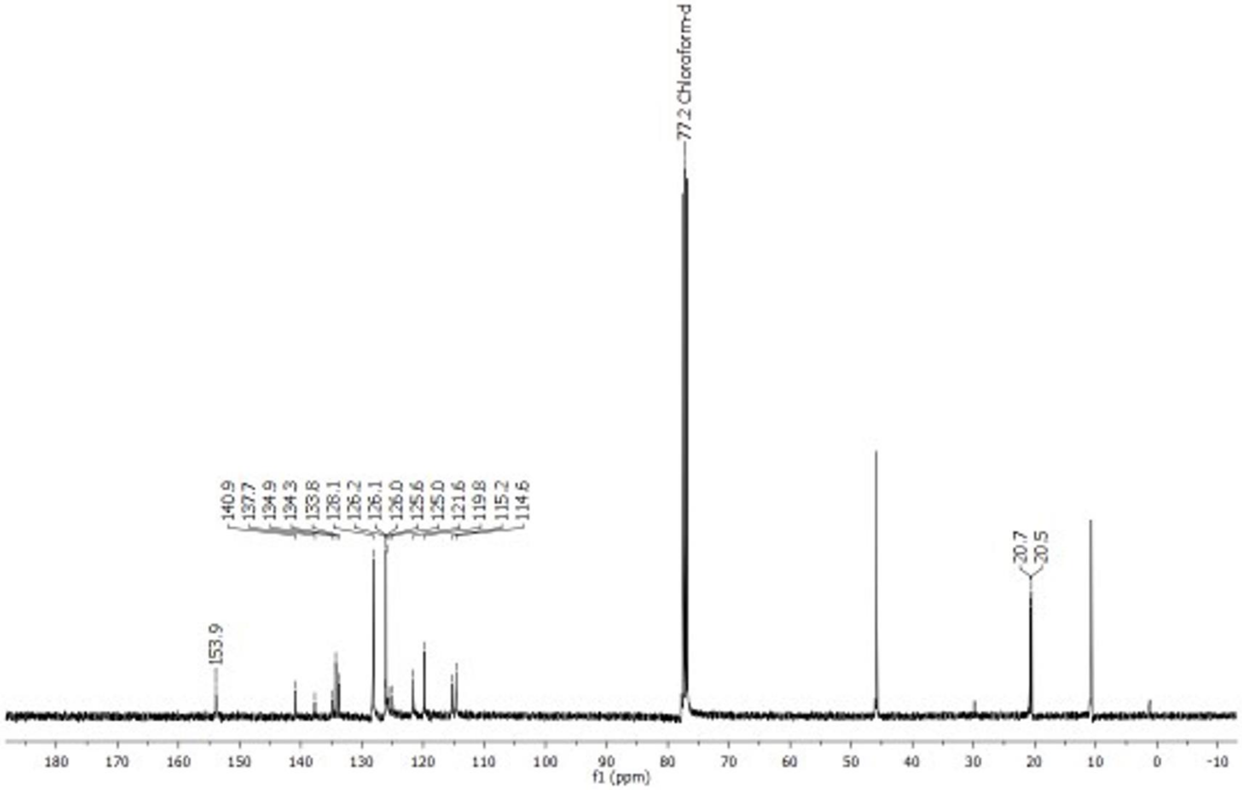
77.2 Chloroform-d
140.9
137.7
134.9
134.3
133.8
128.1
126.2
126.1
126.0
125.6
125.0
121.6
119.8
115.2
114.6
153.9
20.7
20.5
180 170 160 150 140 130 120 110 100 90 80 70 60 50 40 30 20 10 0 -10
f1 (ppm)

IM-24-colF2-200MHz-CDCl3

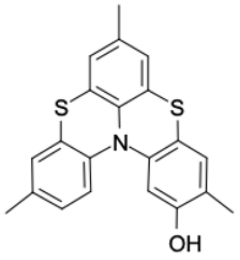

6

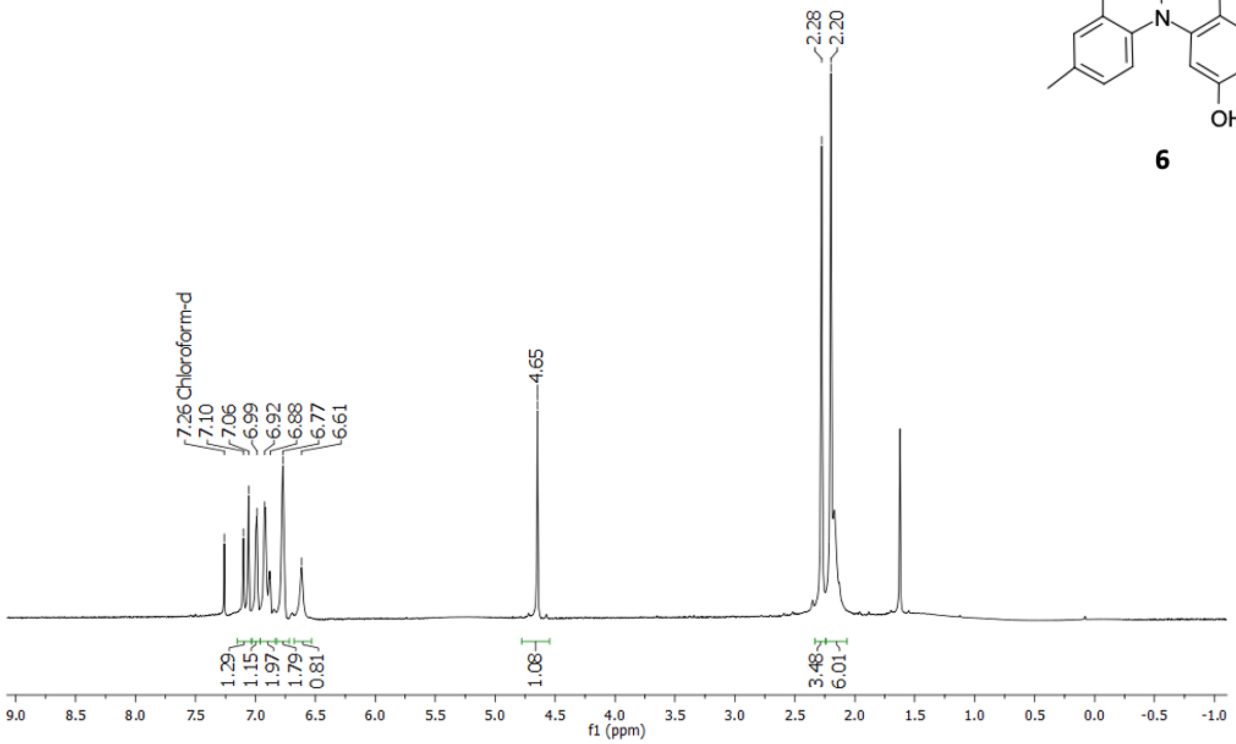

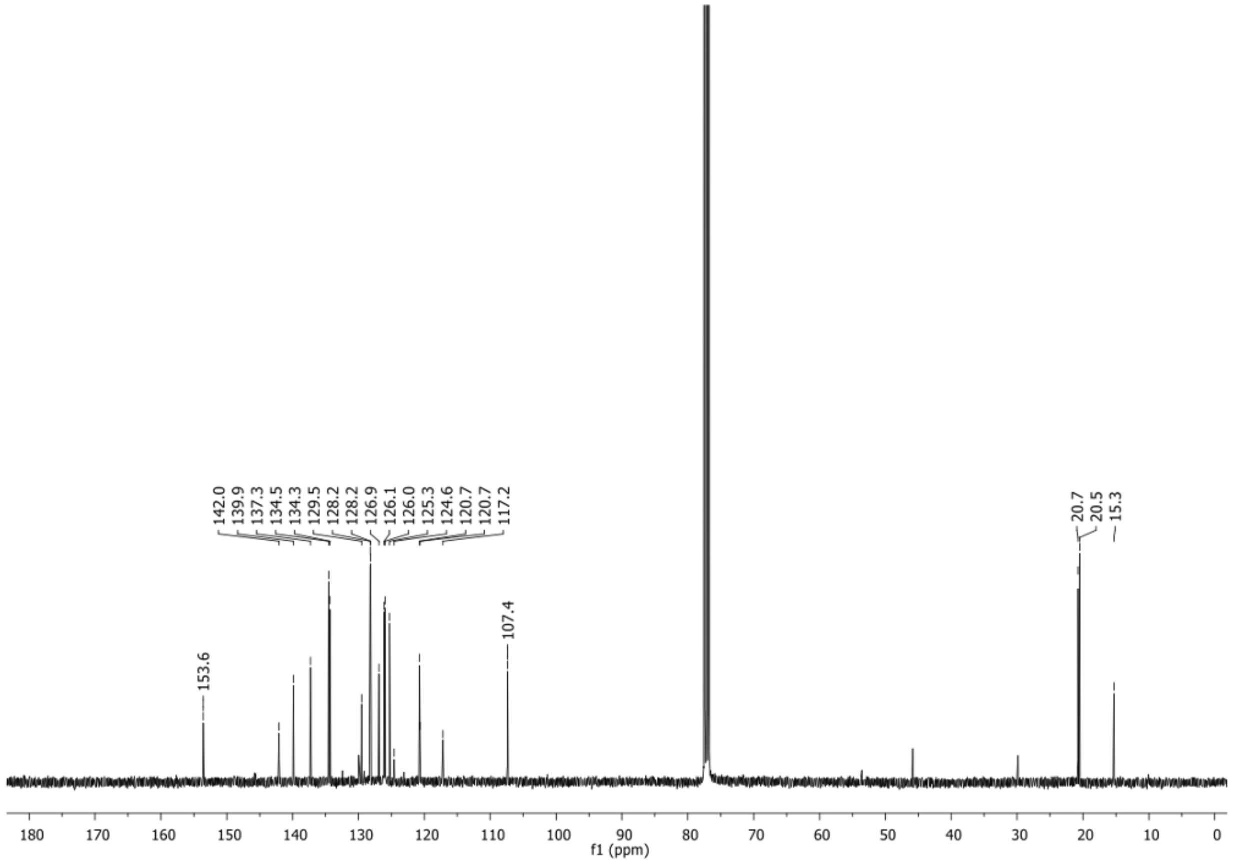

LM241-F2-CDCl3-1HNMR-400MHz

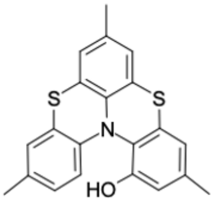

7

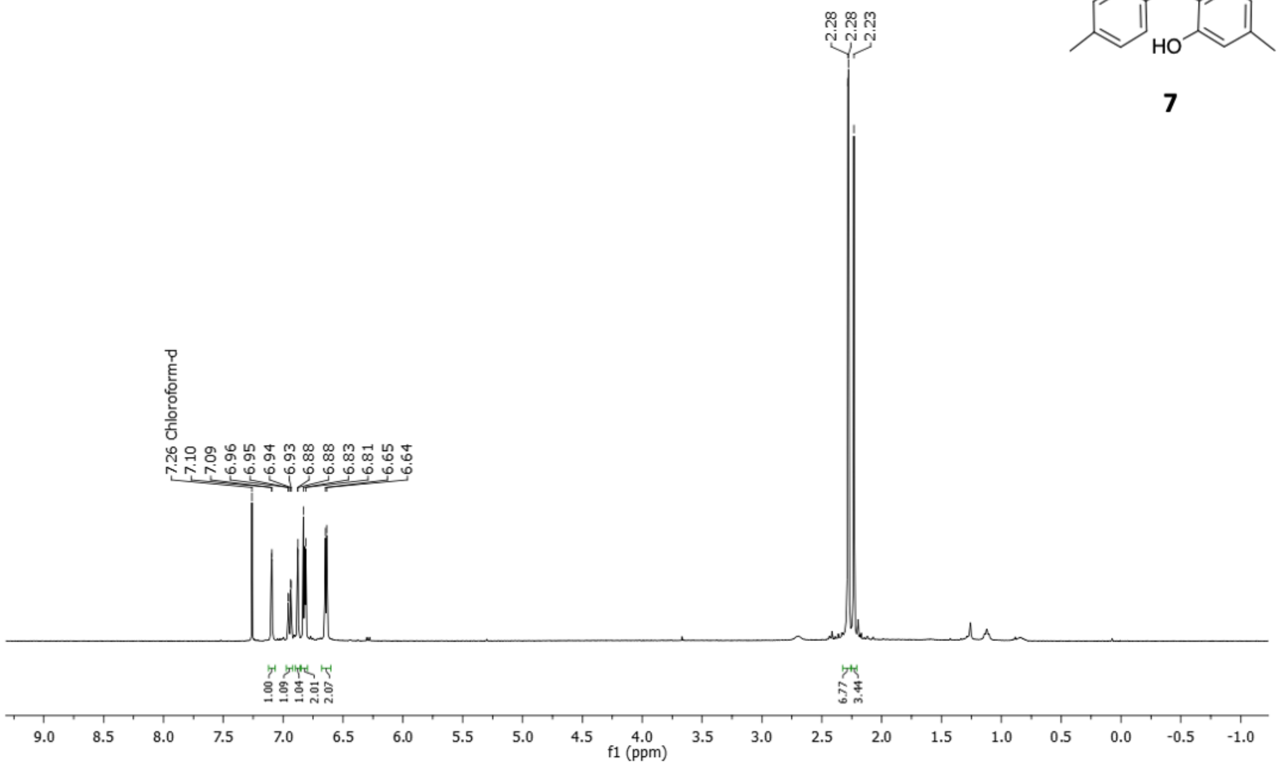

LM241-F2-CDCl3-13CNMR-100MHz

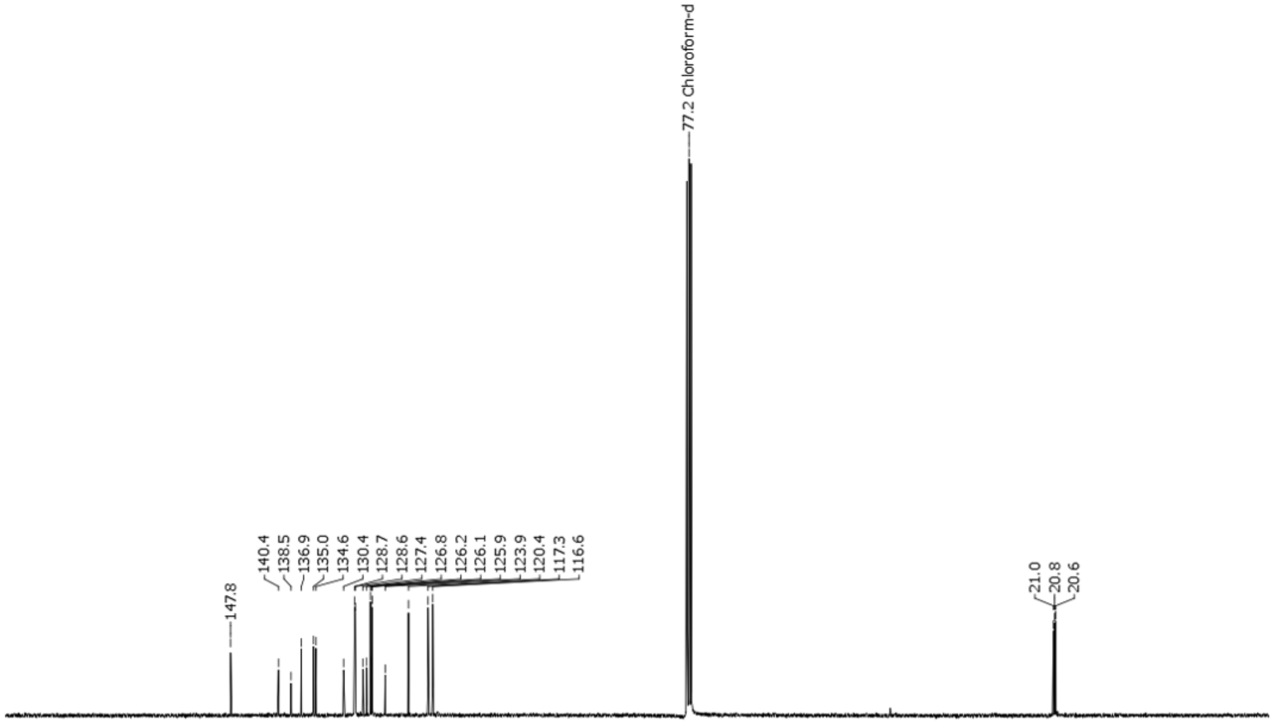

CHIG27-f2-1H-NMR_400MHz

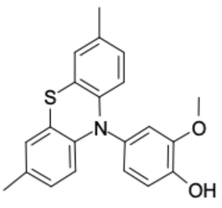

8a

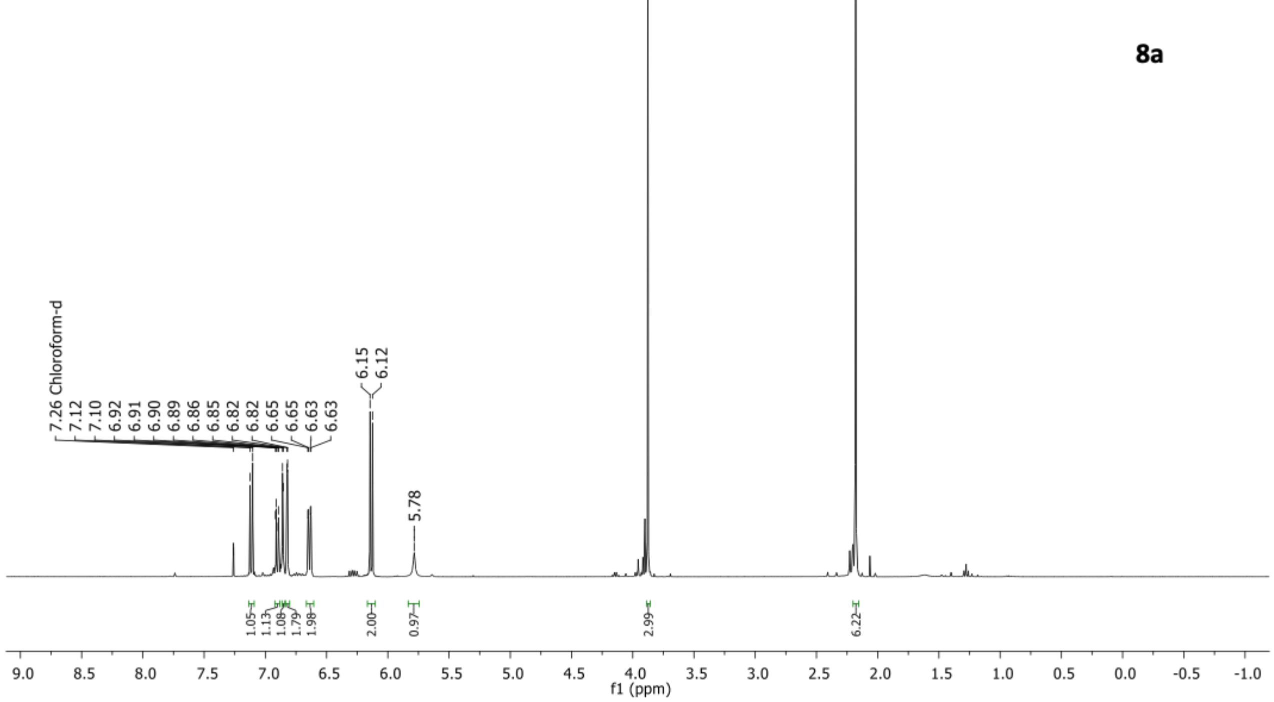

CHIG27-f2-13C-NMR_100MHz

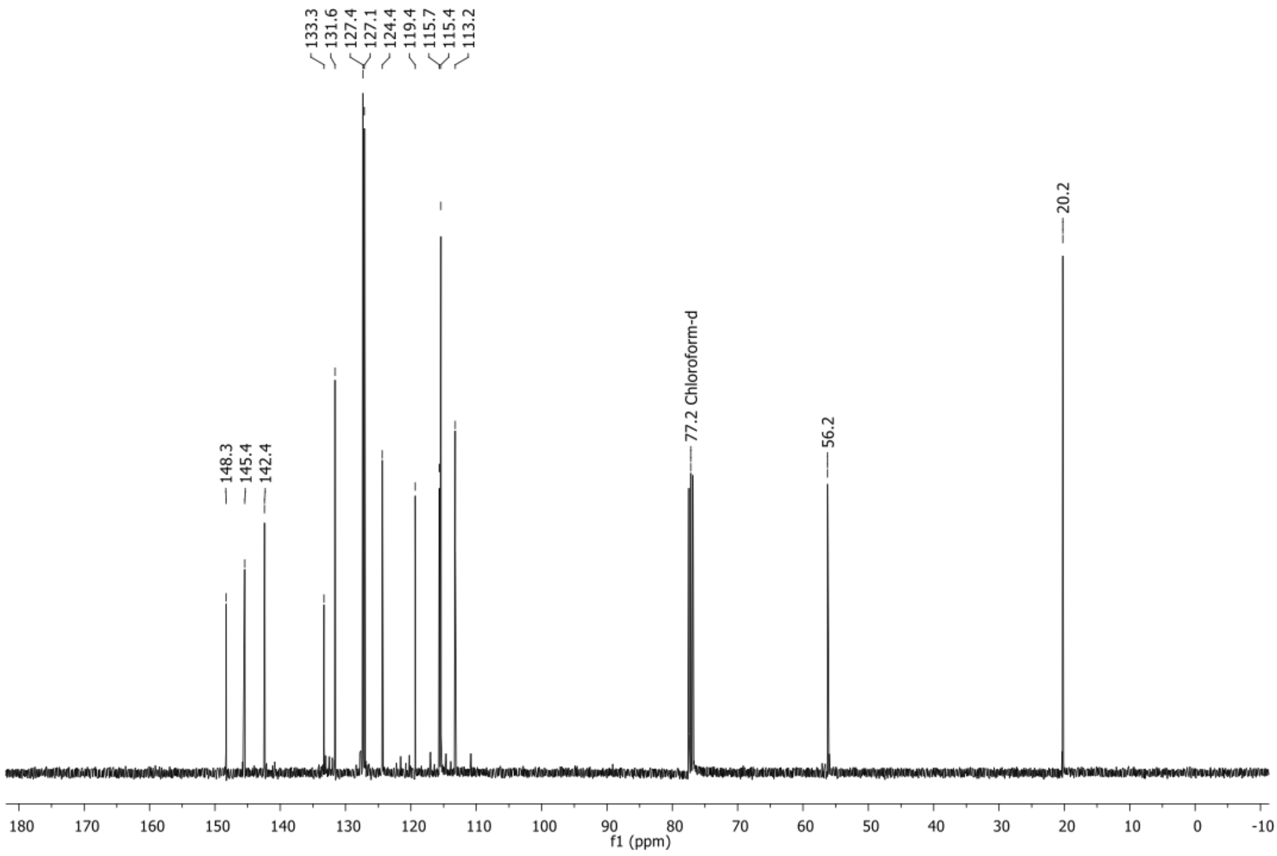

LM182-1H-NMR-400MHz

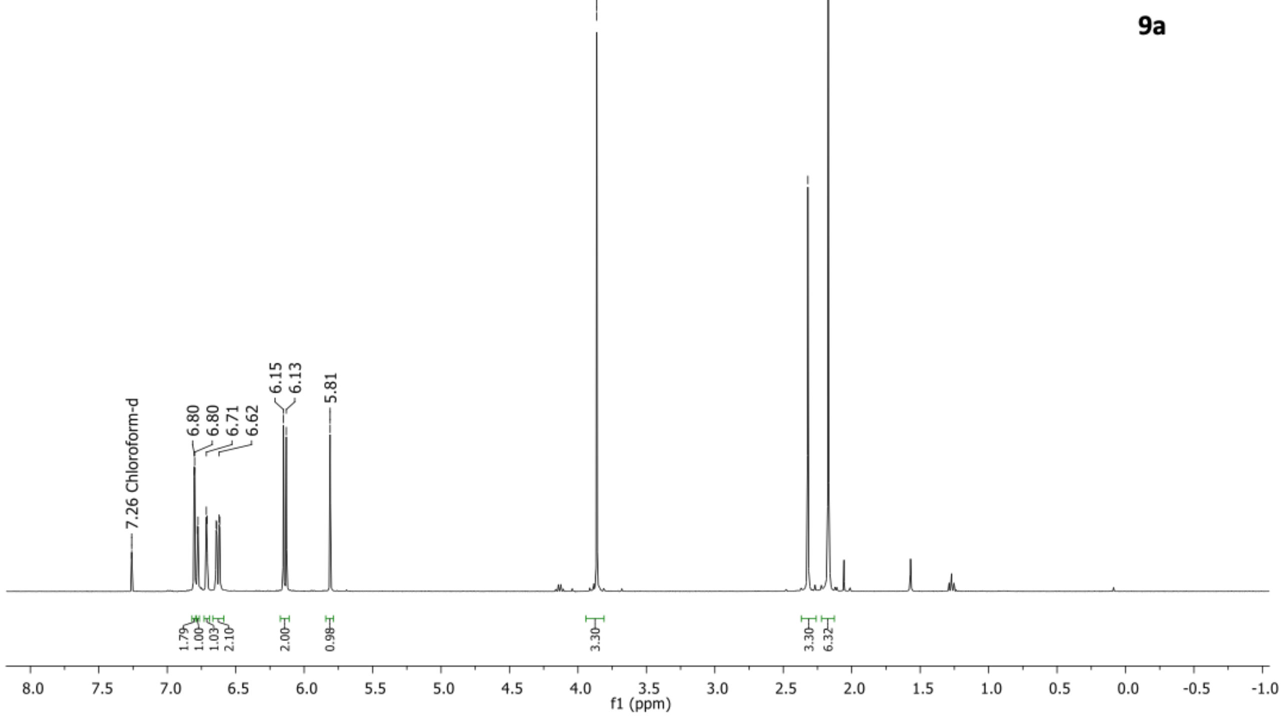

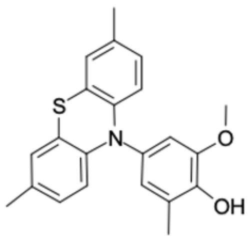

9a

LM182-13C-NMR-100MHz

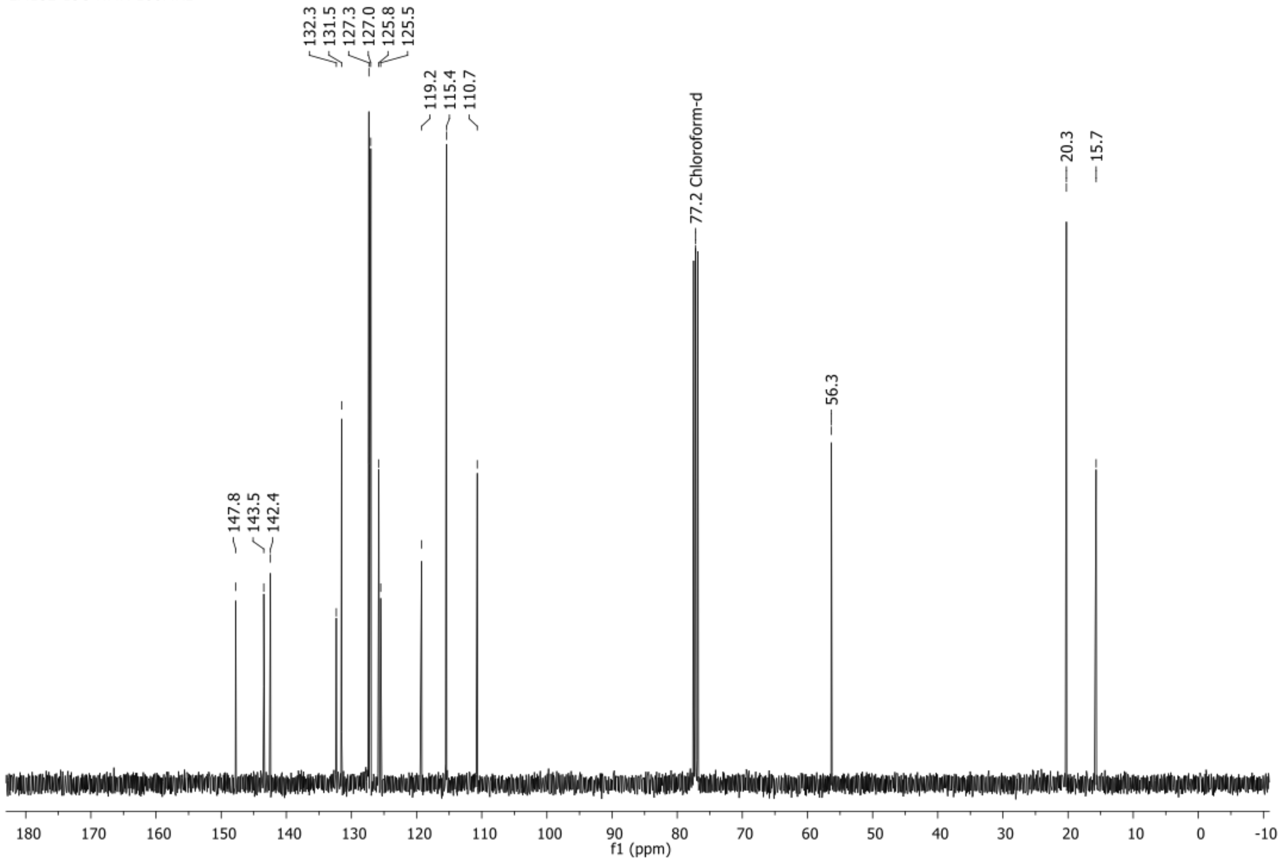

LM201-F2-CDCl3-1HNMR-400MHz

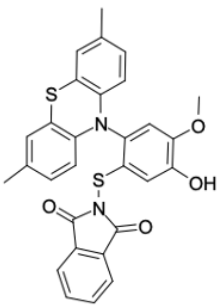

8b

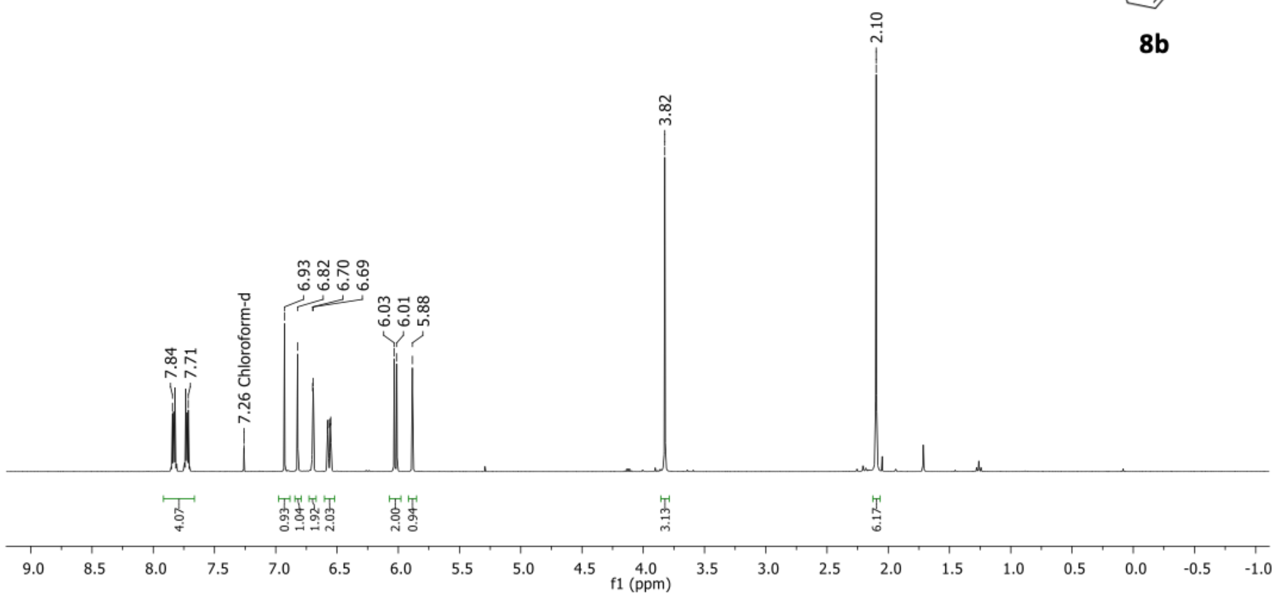

LM201-F2-CDCl3-13CNMR-100MHz

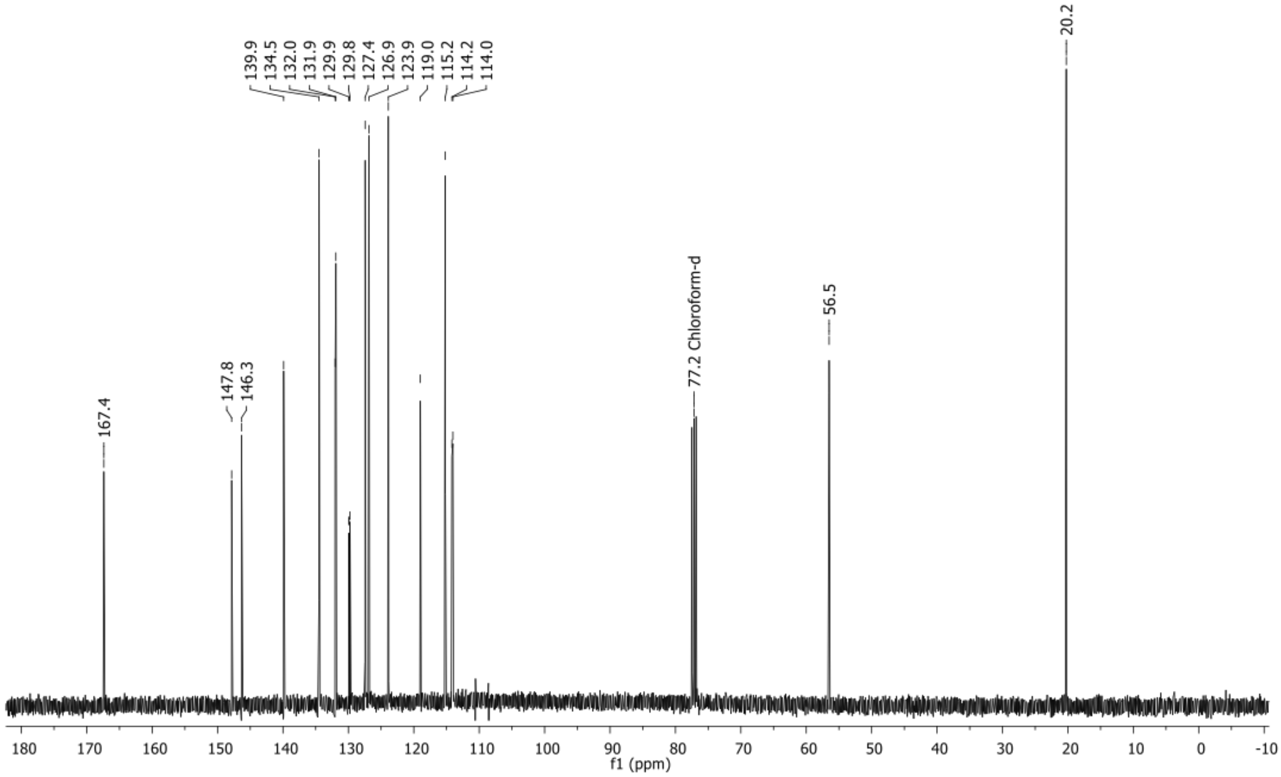

LM187-F2-CDCl3-1HNMR-400MHz

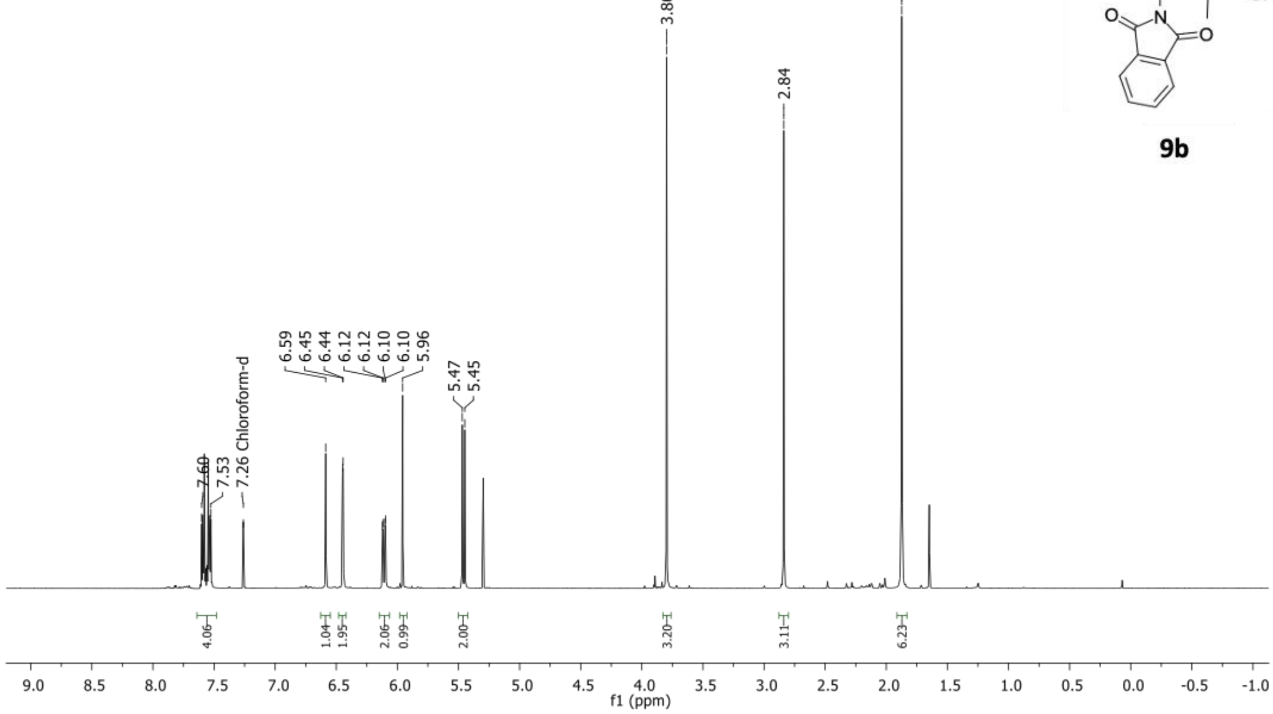

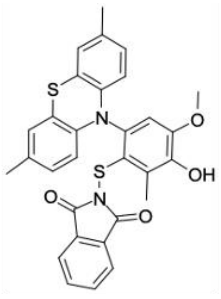

9b

LM187-F2-CDCl3-13CNMR-100MHz

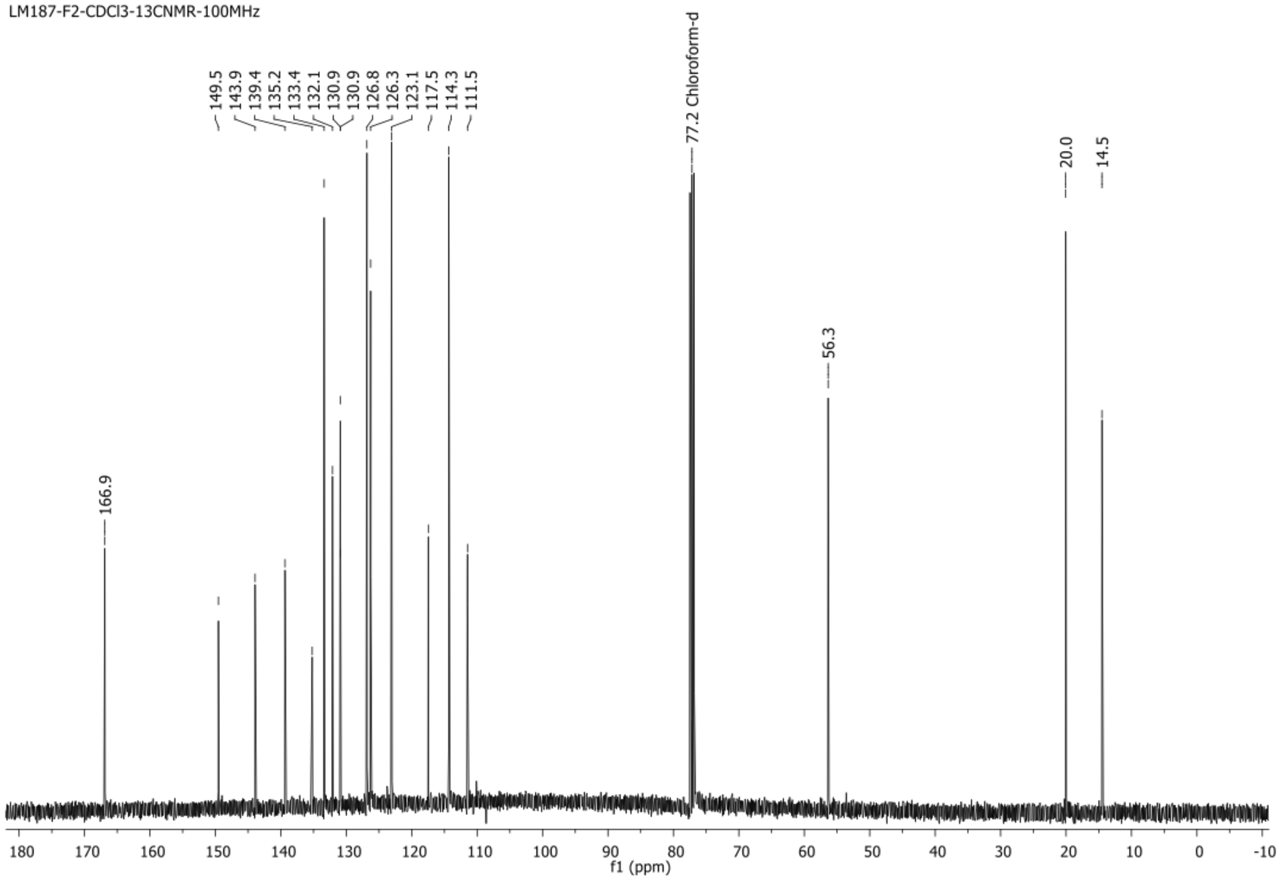

LM204-F2-CD2Cl2-1HNMR-400MHz

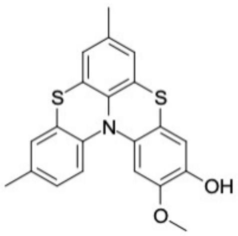

8

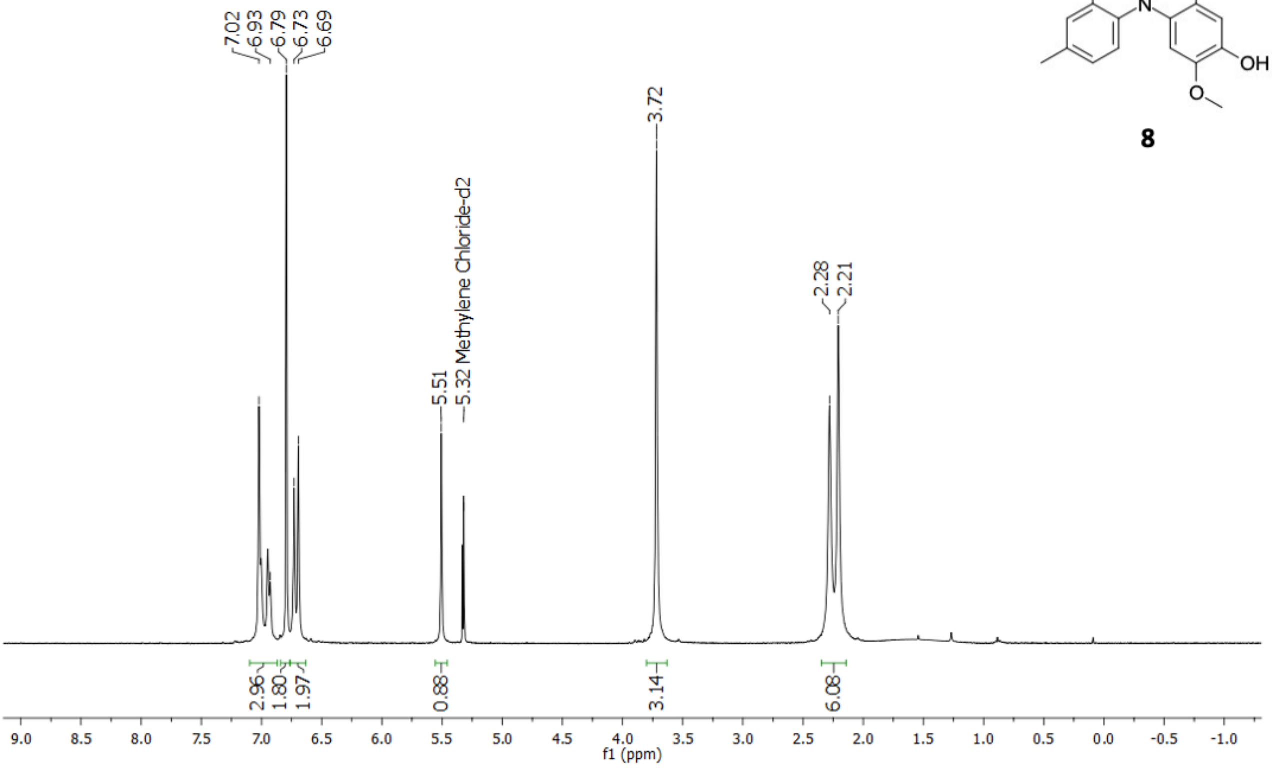

LM204-13CNMR-100MHz-acetone-ditionnito

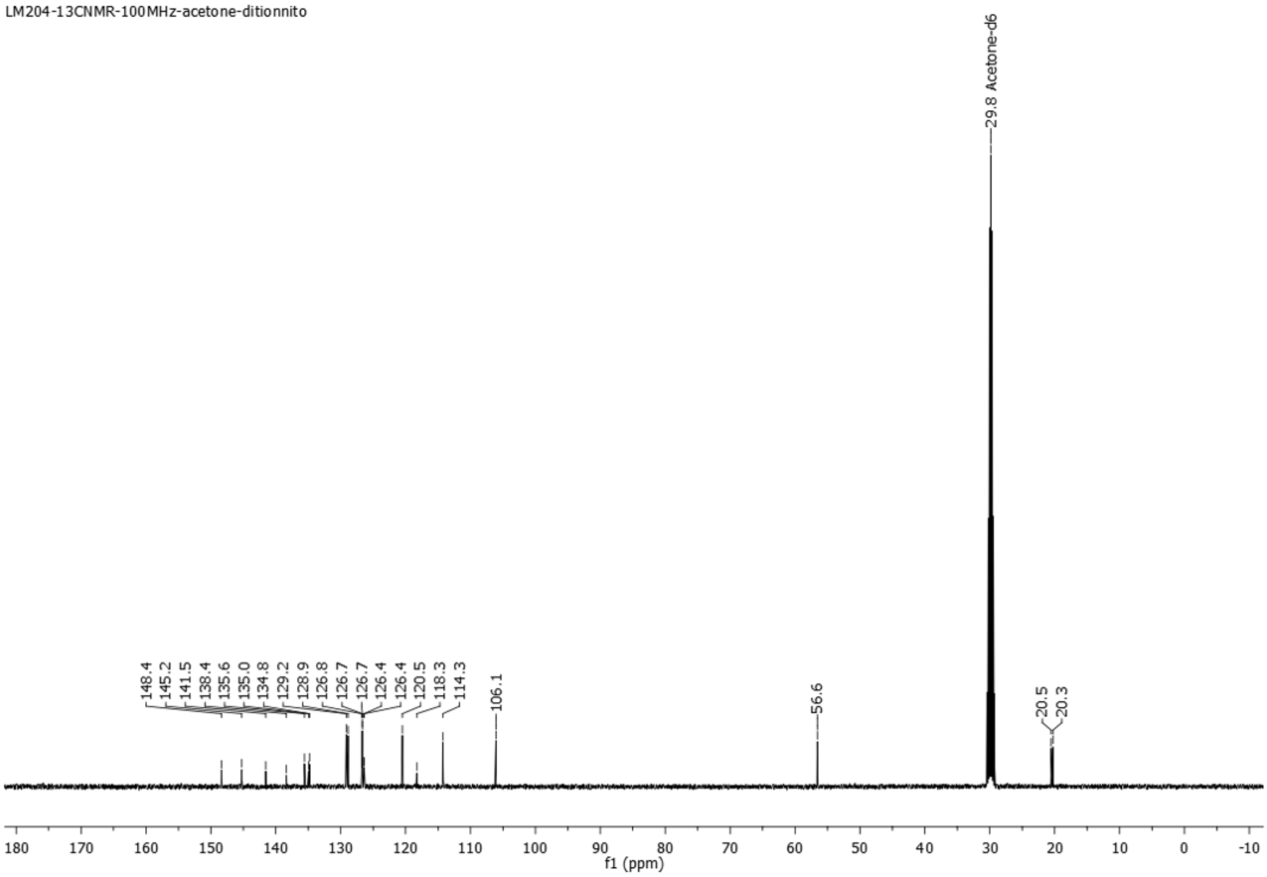

CHIG-43-f1-Acetone-d6

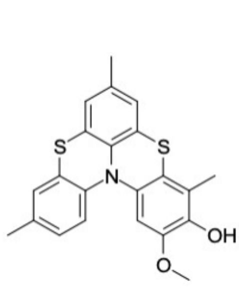

9

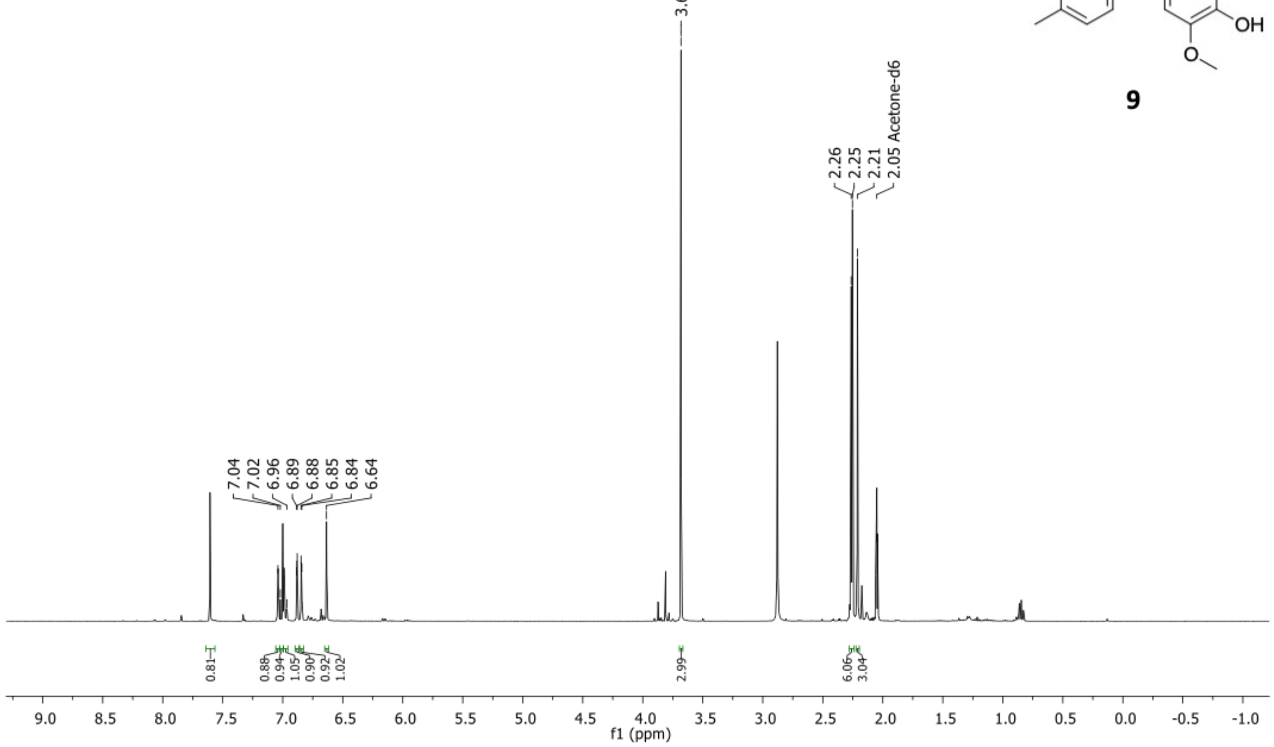

LM199-13CNMR-100MHz-Acetone-Ditionnito

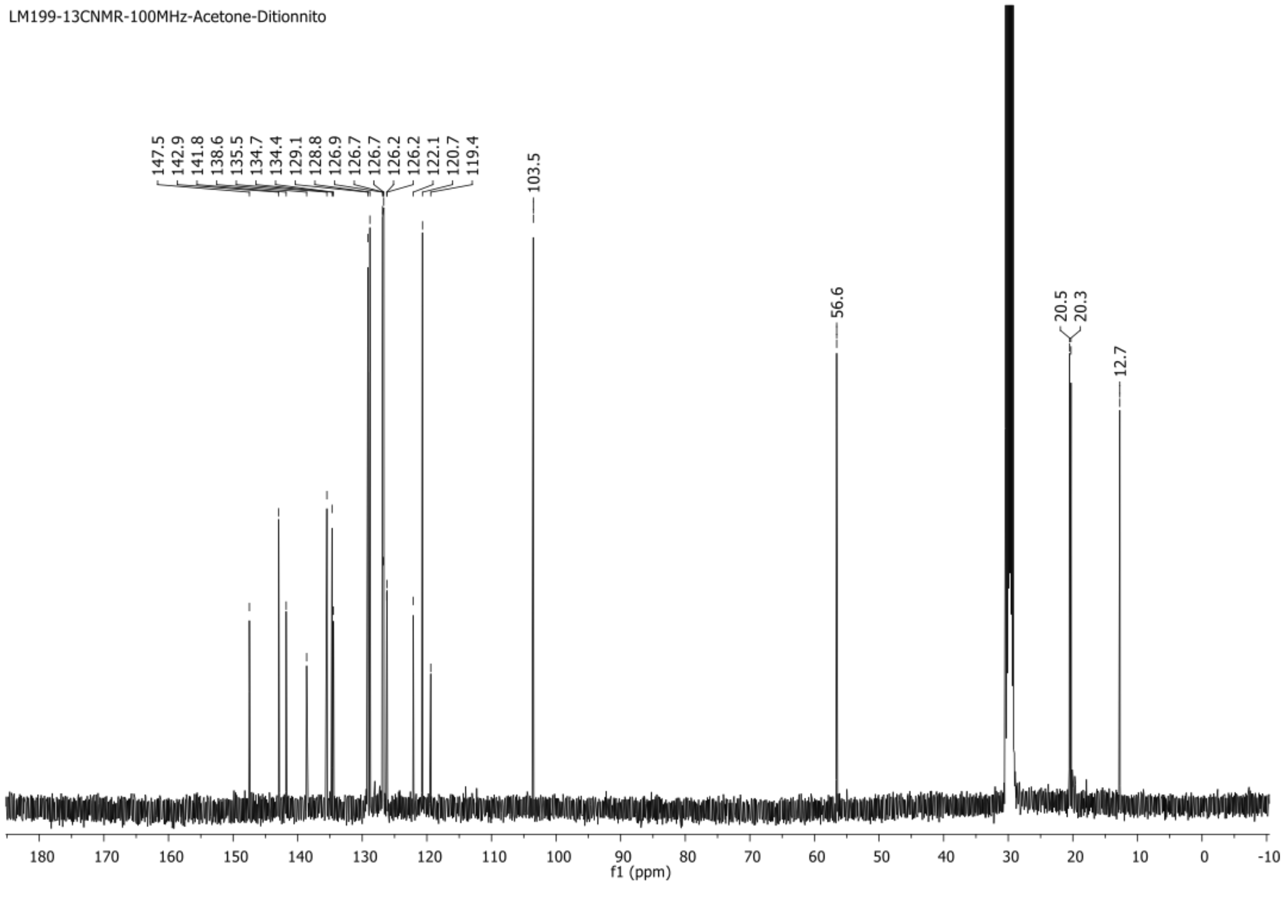

Calculation of the splitting constants

Level of theory: b3lyp/6-31+g(d,p) int=ultrafine

Neutral radicals

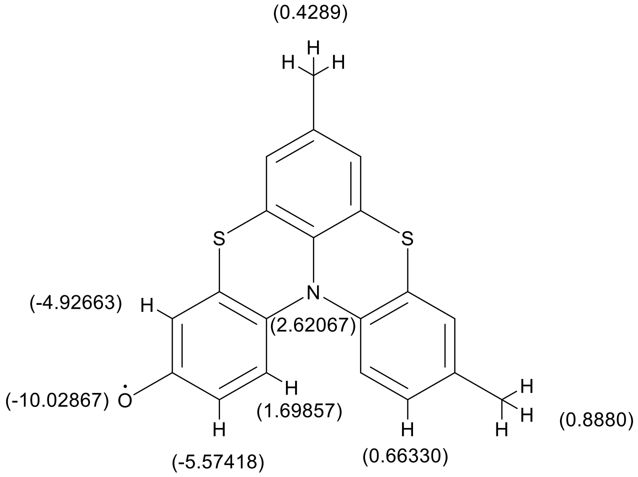

(HH_02_O)

5(-H)•

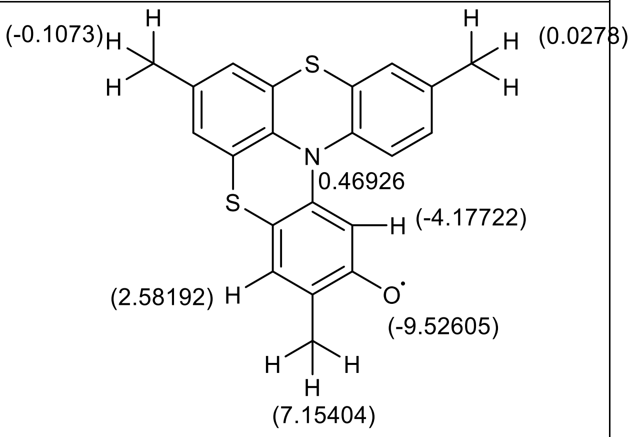

LM236_O

6(-H)•

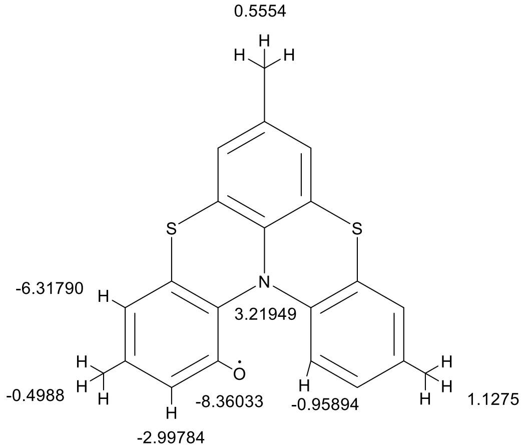

(LM241_F2_O)

7(-H)•

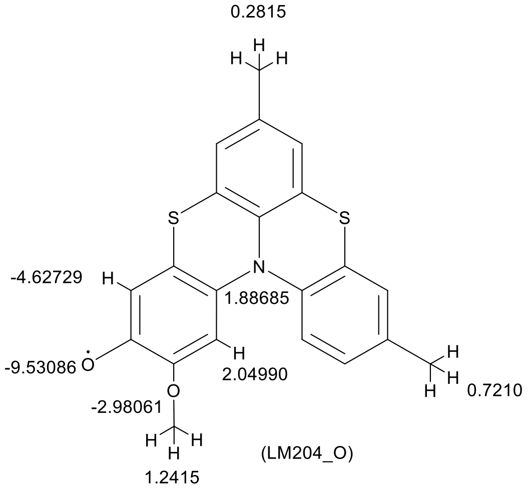

(LM204_O)

8(-H)•

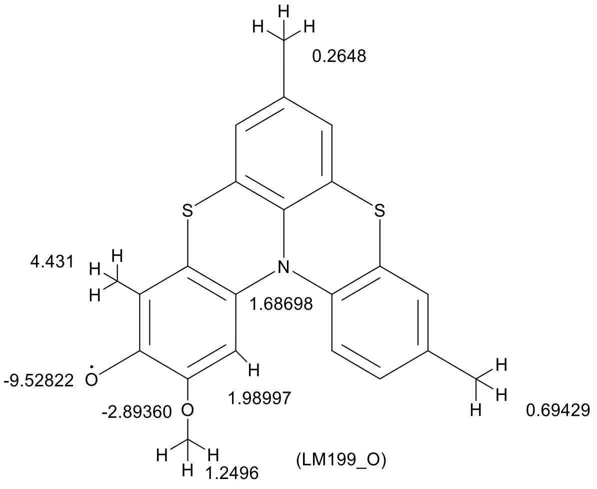

(LM199_O)

9(-H)•

Radical cations	
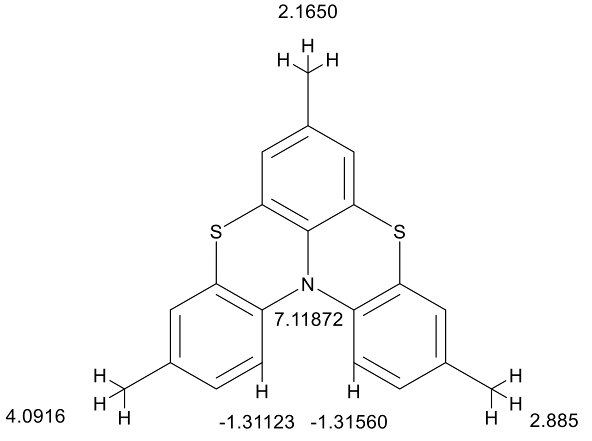<br><br>(HH_01_RC)<br>**1•+**	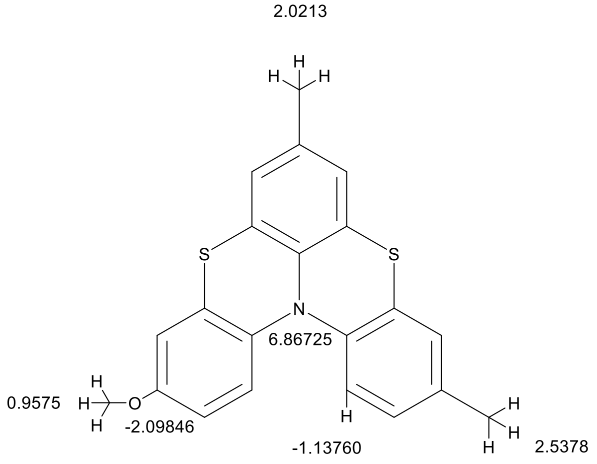<br><br>(SR64_RC)<br>**2•+**
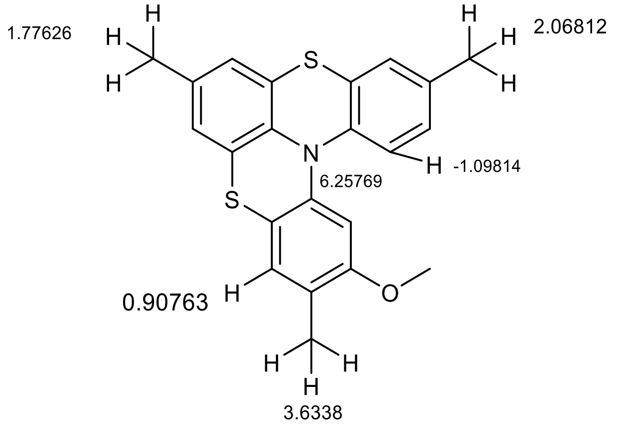<br><br>LM232_RC<br>**3•+**	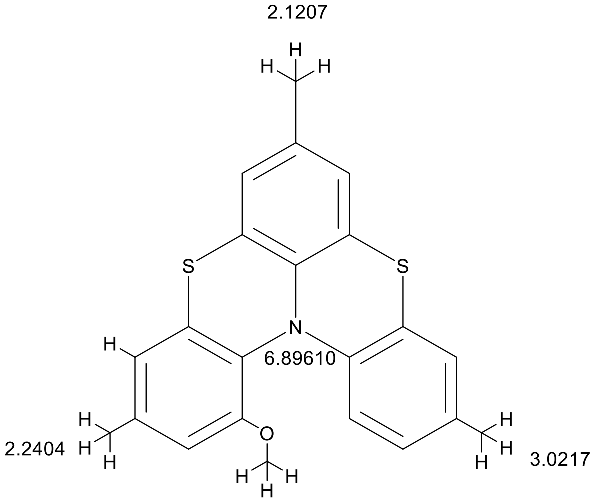<br><br>(LM239_RC)<br>**4•+**
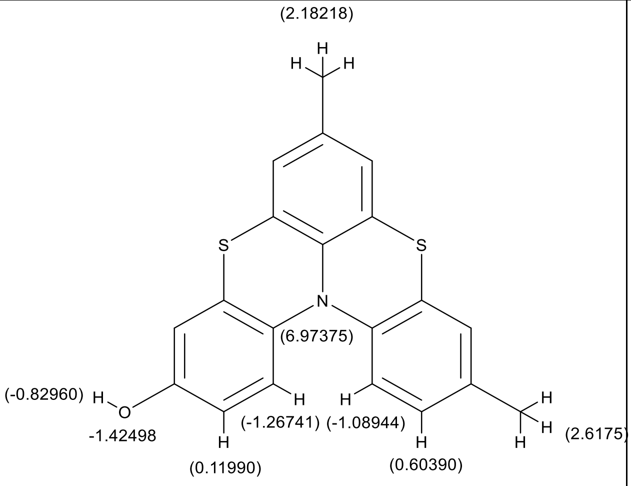<br><br>(HH_02_RC)<br>**5•+**	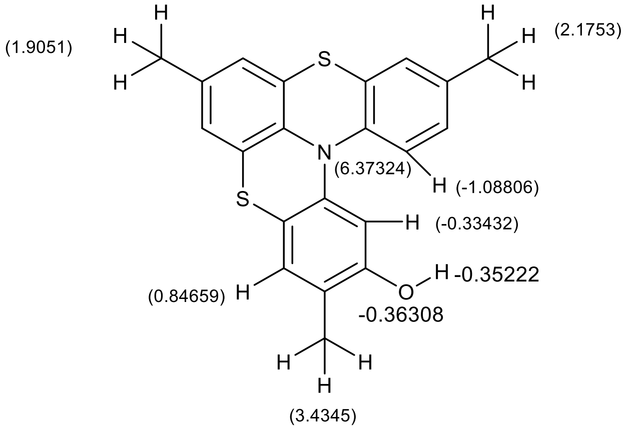<br><br>LM236_RC<br>**6•+**

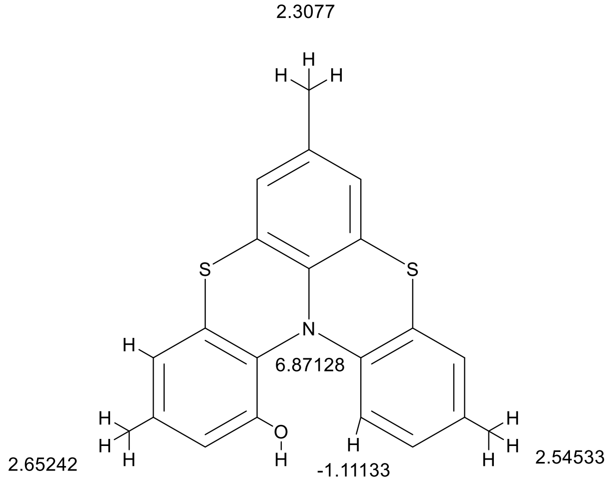

(LM241_F2_RC)

7•+

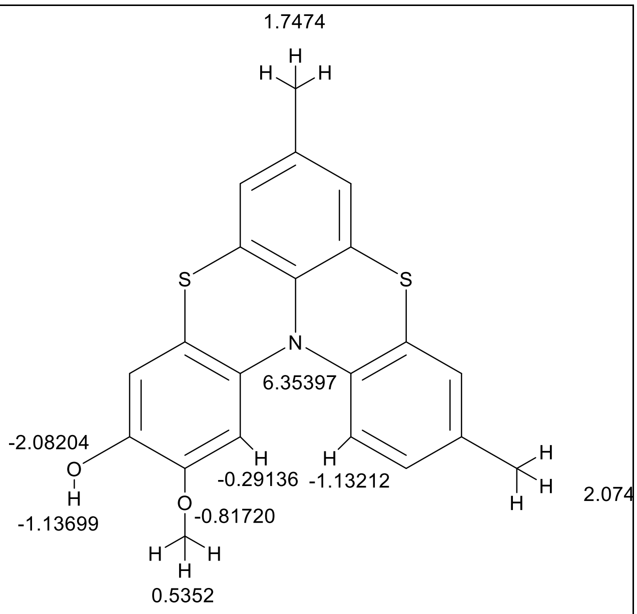

(LM204_RC)

8•+

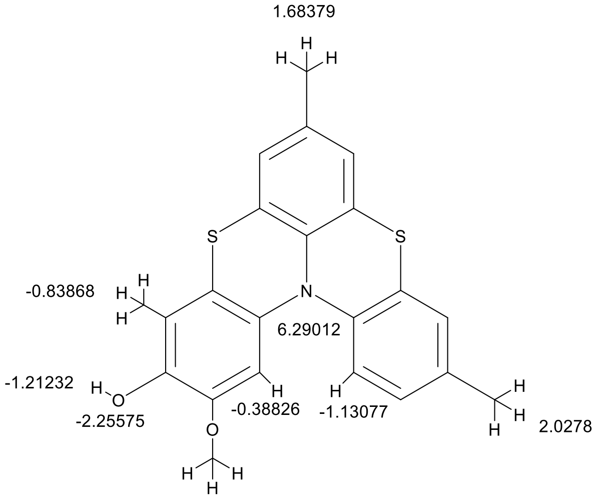

(LM199_RC)

9•+

Reference:

1SI: L. Bering, L. D’Ottavio, G. Sirvinskaite, A. P. Antonchick *Chem.Commun.* **2018**, *54*, 13022.
